# Green Synthesis of Silver Nanoparticles Using *Artemisia absinthium* and *Ziziphus vulgaris* Extracts: Comparative Evaluation of Antibacterial, Anti-Inflammatory, Wound-Healing and Anticancer Activities

**DOI:** 10.3390/ph19101630

**Published:** 2026-10-14

**Authors:** Mohammed Gamal, Asma B. Omer, Asmaa Saleh, Roula Bayram, Ibrahim A. Naguib, Mohamed Ahmed Elsayed, Safa Mousa, Hamad M. Adress Hasan

**Affiliations:** 1 Pharmaceutical Analytical Chemistry Department, Faculty of Pharmacy, Beni-Suef University, Alshaheed Shehata Ahmed Hegazy St., Beni-Suef 62574, Egypt; 2 Department of Health Science, College of Health and Rehabilitation Sciences, Princess Nourah bint Abdulrahman University, P.O. Box 84428, Riyadh 11671, Saudi Arabia; abalfaki@pnu.edu.sa; 3 Department of Pharmaceutical Sciences, College of Pharmacy, Princess Nourah bint Abdulrahman University, P.O. Box 84428, Riyadh 11671, Saudi Arabia; asali@pnu.edu.sa; 4 Pharmaceutical Science, Pharmacy Program, Batterjee Medical College, Jeddah 21442, Saudi Arabia; lab3.jed@bmc.edu.sa; 5 Department of Quality Control and Pharmaceutical Chemistry, Faculty of Pharmacy, University of Aleppo, Aleppo P.O. Box 12212, Syria; 6 Department of Pharmaceutical Chemistry, College of Pharmacy, Taif University, Taif 21944, Saudi Arabia; 7 Pharmaceutical Analytical Chemistry Department, Faculty of Pharmacy, Fayoum University, Fayoum 63514, Egypt; 8 Chemistry Department, Faculty of Science, AlQuba Campus, Derna University, Derna P.O. Box 125, Libya; 9 Chemistry Department, Faculty of Science, Omar Al-Mukhtar University, Bayda P.O. Box 991, Libya

**Keywords:** green synthesis, silver nanoparticles, *Artemisia absinthium*, *Ziziphus vulgaris*, antibacterial activity, anti-inflammatory activity, cytotoxicity, wound-healing

## Abstract

**Background:** Plant-mediated synthesis of silver nanoparticles (AgNPs) has emerged as a sustainable alternative to conventional chemical methods, offering environmentally friendly routes for producing nanomaterials with biomedical potential. This study employed aqueous extracts of *Artemisia absinthium* and *Ziziphus vulgaris* to synthesize AgNPs and evaluate their antibacterial, anti-inflammatory, anticancer, wound healing activities. **Methods:** Silver nanoparticles were synthesized by mixing aqueous plant extracts with 1 mM silver nitrate under ambient conditions. The formation of AgNPs was confirmed by UV–Visible spectroscopy, while morphology and structure were characterized using Transmission Electron Microscopy (TEM), Scanning Electron Microscopy (SEM), and Fourier-transform infrared (FTIR) spectroscopy. Moreover, the biosynthesized silver nanoparticles were further characterized by dynamic light scattering (DLS) and zeta potential measurements to determine hydrodynamic size and surface charge, and by X-ray diffraction (XRD) to confirm crystalline phase. Energy-dispersive X-ray spectroscopy (EDX) was also performed to assess elemental composition, providing a comprehensive physicochemical profile of the plant-mediated AgNPs. Antibacterial activity was tested against *Escherichia coli* and *Staphylococcus aureus* using the agar well diffusion, minimal inhibitory concentration (MIC) and minimal bactericidal concentration (MBC) methods. Anti-inflammatory activity was evaluated by hypotonicity-induced human red blood cell (HRBC) membrane stabilization assay. Cytotoxic activity was evaluated using the MTT assay against a panel of human cell lines comprising MCF-7 breast cancer cells, A549 lung cancer cells, HepG2 hepatic cancer cells, and WI-38 non-cancerous lung fibroblasts to assess both anticancer potency and selectivity toward normal cells. Scratch wound healing was also evaluated. **Results:** The biosynthesized AgNPs showed surface plasmon resonance bands at 420–450 nm and mainly spherical particles with sizes of 15–22 nm. FTIR analysis indicated hydroxyl and carbonyl groups involved in nanoparticle reduction and stabilization. DLS and zeta potential analyses revealed monomodal size distributions and strongly negative surface charges (approximately −33 to −34 mV), indicating homogeneous, electrostatically stabilized colloids. XRD patterns confirmed a face-centered cubic metallic silver phase with a dominant (111) orientation, while EDX spectra verified the presence of elemental silver alongside signals from the plant-derived capping layer. The nanoparticles displayed remarkable antibacterial activity against both bacteria, strong inhibition of erythrocyte hemolysis, and dose-dependent cytotoxicity toward MCF-7 cells with IC_50_ values of 19–21 µg mL^−1^. MIC values ranged from 15.62 to 125 µg mL^−1^, with the lowest MIC observed for *Ziziphus* stem-mediated AgNPs (15.62 µg mL^−1^). All AgNPs samples exhibited enhanced wound healing, with closure percentages reaching up to 93.4% in *Ziziphus* leaves compared to WI-38 control (77.6%). Good colloidal stability was maintained for 90 days. **Conclusions:** These findings show that *Artemisia absinthium* and *Ziziphus vulgaris* are effective sources for green synthesis of stable AgNPs with multifunctional in vitro biological activities. This pro-healing effect is consistent with their demonstrated antimicrobial activity, supporting their dual functionality as both antibacterial and wound-healing materials. However, the biological and therapeutic activities require further in vivo confirmation. The results support their biomedical and environmentally sustainable nanotechnology applications.

## 1. Introduction

Nanotechnology allows the synthesis of materials with unique optical, chemical, and biological properties that arise from nanoscale dimensions. Among noble-metal nanomaterials, silver nanoparticles (AgNPs) are mostly smart because of their high electrical conductivity, strong surface plasmon resonance, and well-documented antimicrobial, anti-inflammatory, and anticancer effects [1,2]. Traditional physical and chemical synthesis routes including thermal decomposition, photochemical reduction, and sodium-borohydride reduction can produce well-defined nanoparticles but typically require expensive equipment, high energy input, and toxic reagents that generate hazardous by-products [3,4,5]. These limitations motivate the development of greener, safer, and more economical alternatives.

Green synthesis has become a prominent environmentally friendly approach, in which organisms like plants, fungi, bacteria, and algae serve as natural reducing and stabilizing agents, removing the use of hazardous chemical reagents. [6]. Among these, plant-mediated synthesis is particularly attractive because plant tissues contain abundant metabolites, flavonoids, phenolics, alkaloids, terpenoids, polysaccharides, and proteins that simultaneously reduce Ag^+^ ions to metallic Ag^0^ and cap the nanoparticles to prevent aggregation [7]. Numerous reports demonstrate successful plant-based synthesis of AgNPs with tunable size and morphology. For example, *Azadirachta indica* (neem) leaf broth rapidly yields stable silver and gold nanoparticles under ambient conditions [8]; *Carica papaya* and *Capsicum annuum* extracts produce AgNPs with strong antibacterial activity [9,10]; and olive (*Olea europaea*) leaf extract generates uniform particles exhibiting broad antimicrobial effects [11]. *Zanthoxylum armatum* leaves produced crystalline AgNPs (15–50 nm) capable of catalyzing dye reduction [12], while Origanum vulgare acted as both a reducing and capping agent to yield face-centered cubic AgNPs with potent antibacterial and antifungal properties [13]. Other notable examples include *Ipomoea pescaprae*, *Withania somnifera*, and *Perilla frutescens*, whose extracts provided nanoparticles with remarkable anticancer and anti-inflammatory activities [14,15,16].

Medicinal plants offer a particular advantage for green synthesis because their intrinsic therapeutic phytochemicals can synergistically enhance the biological activities of the resulting nanoparticles. *Artemisia* species are rich in flavonoids and sesquiterpene lactones and are traditionally used for their antimicrobial, antioxidant, and anti-inflammatory effects [17,18]. *Ziziphus* species contain saponins, triterpenoids, and polyphenols that exhibit antibacterial, anti-inflammatory, and anticancer activities [19,20]. The plant-mediated reduction of Ag^+^ ions to metallic Ag^0^ nanoparticles by phytochemicals such as phenolics, flavonoids, terpenoids, proteins, and polysaccharides is well documented in the literature [6,7]. These biomolecules, particularly those containing hydroxyl (–OH) and carbonyl (C=O) functional groups, act as natural reducing and stabilizing agents during nanoparticle synthesis. In the present study, *Artemisia absinthium* and *Ziziphus vulgaris* extracts are presumed to follow this established reduction mechanism, while also potentially imparting plant-specific functional characteristics to the resulting nanoparticles.

Despite the extensive literature on plant-based synthesis of silver nanoparticles (AgNPs), comparative investigations employing different medicinal plants under strictly identical experimental conditions remain limited. Consequently, systematic evaluations of the resulting nanoparticles’ antibacterial, anti-inflammatory, and anticancer properties are still relatively scarce.

The present approach introduces a direct side-by-side comparison of two phytochemically distinct and widely available Libyan plants *Artemisia absinthium* and *Ziziphus vulgaris* for the green synthesis of AgNPs using the same extraction procedures and reaction conditions. This strategy enables a clear assessment of how differences in phytochemical composition influence nanoparticle formation, structural characteristics, colloidal stability, and multifunctional biological activity.

**Figure 1:**
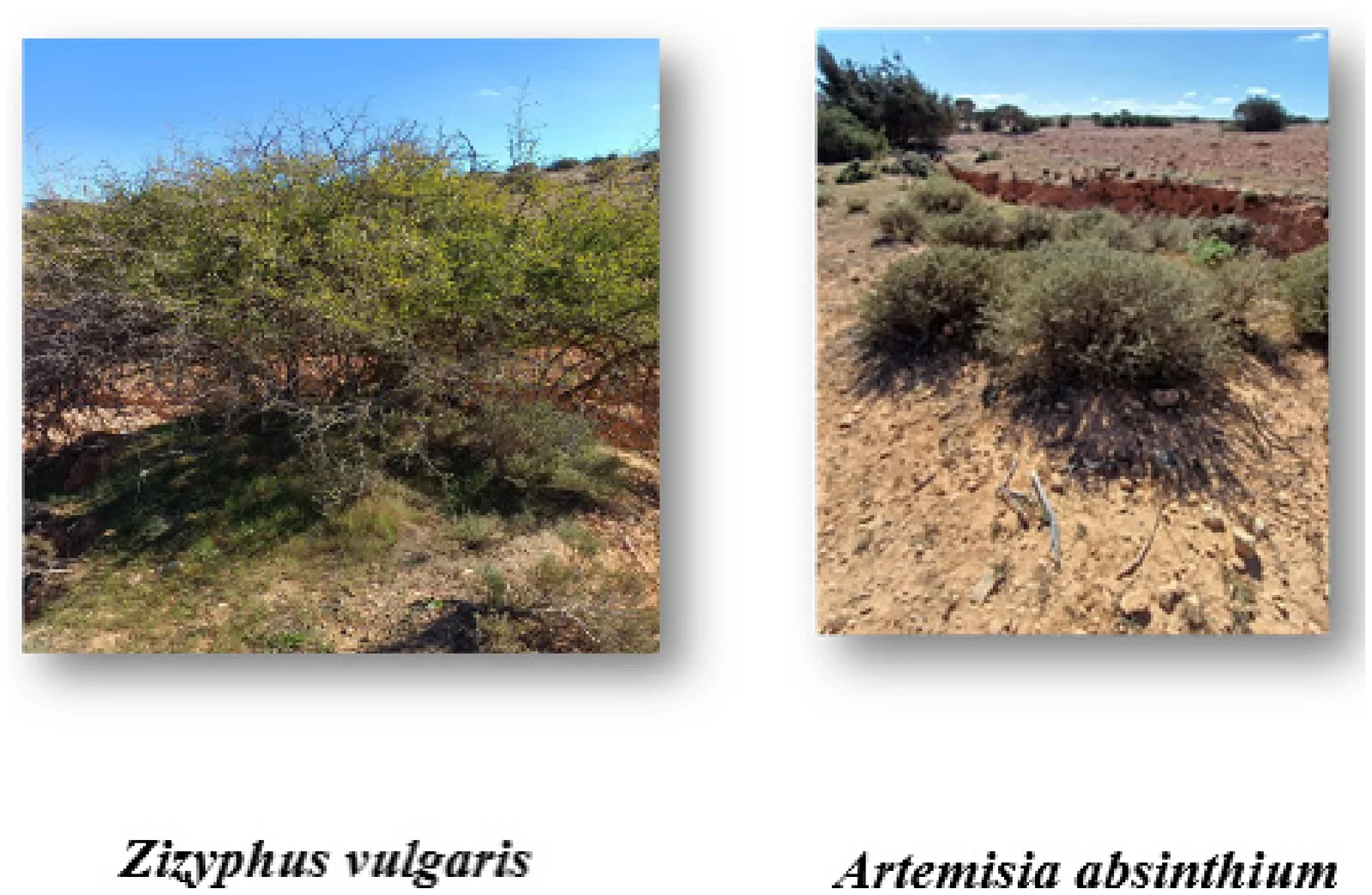
*Artemisia absinthium* and *Ziziphus vulgaris* plants.

Importantly, *Artemisia* and *Ziziphus*, (Figure 1) are abundant in Libya and often regarded as underutilized biomass that is occasionally burned as agricultural waste, contributing to atmospheric pollution. Harnessing these readily available plants as natural nano-factories not only adds pharmaceutical and medicinal values but also promotes environmental sustainability by converting potential waste into high-value nanomaterials, aligning with the principles of green chemistry and circular bioeconomy. Accordingly, the objectives of this work were to (i) synthesize AgNPs using aqueous leaf and stem extracts of *Artemisia absinthium* and *Ziziphus vulgaris* under ambient, chemical-free conditions; (ii) characterize the nanoparticles’ structural and optical properties; (iii) evaluate their antibacterial activity against *Escherichia coli* and *Staphylococcus aureus*; (iv) assess their anti-inflammatory activity using a hypotonicity-induced hemolysis model; (v) determine their cytotoxic effects on a panel of human cell lines comprising MCF-7 breast cancer cells, A549 lung cancer cells, HepG2 hepatic cancer cells, and WI-38 non-cancerous lung fibroblasts; and (vi) assess potential in vitro wound healing. By highlighting the influence of plant phytochemistry on nanoparticle properties while valorizing widely distributed Libyan flora, this work provides new insights into sustainable nanomaterial design and the development of eco-friendly therapeutic agents.

## 2. Results

Silver nanoparticles (AgNPs) were successfully synthesized using aqueous leaf and stem extracts of *Artemisia absinthium* and *Ziziphus vulgaris* through the plant-mediated reduction of silver nitrate. The formation of AgNPs was visually indicated by a color change from yellow to dark brown and confirmed by FTIR, SEM, and TEM analyses, which revealed nearly spherical particles with sizes ranging from 15 to 22 nm. Moreover, the biosynthesized AgNPs were further characterized by DLS and zeta potential measurements to determine hydrodynamic size and surface charge, and by XRD to confirm crystalline phase. EDX was also performed to assess elemental composition, providing a comprehensive physicochemical profile of the plant-mediated AgNPs. The biosynthesized AgNPs were further evaluated for their antibacterial, anti-inflammatory, wound healing, and anticancer activities, demonstrating significant inhibitory effects against pathogenic bacteria, strong red blood cell membrane stabilization, enhanced closure percentages of in vitro wounds, and dose-dependent cytotoxicity toward cancer cell lines.

### 2.1. Visual Observation of Silver Nanoparticle Formation

**Figure 2:**
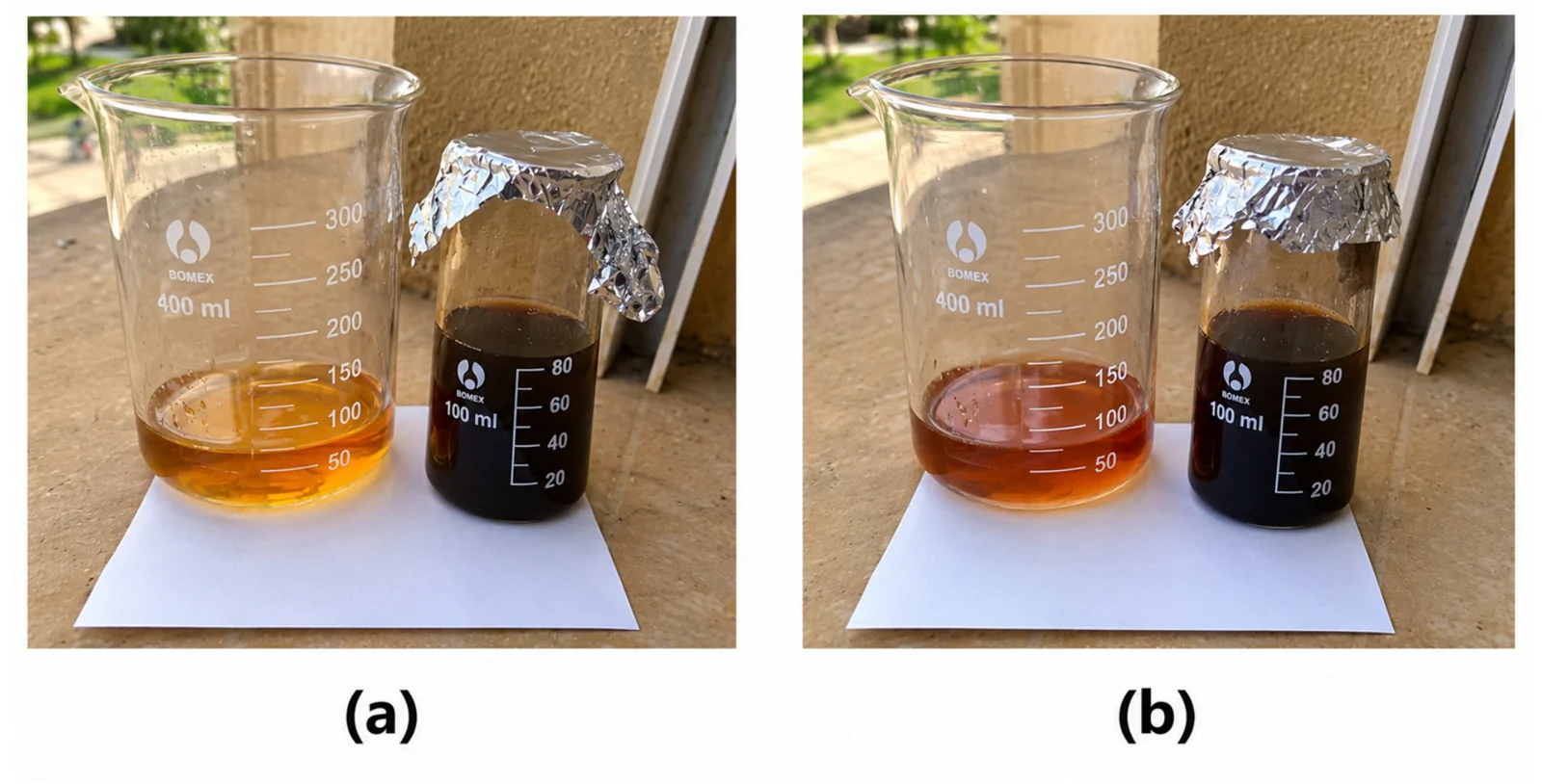
*Artemisia* extract before (**a**) and after (**b**) the addition of Ag^+^.

**Figure 3:**
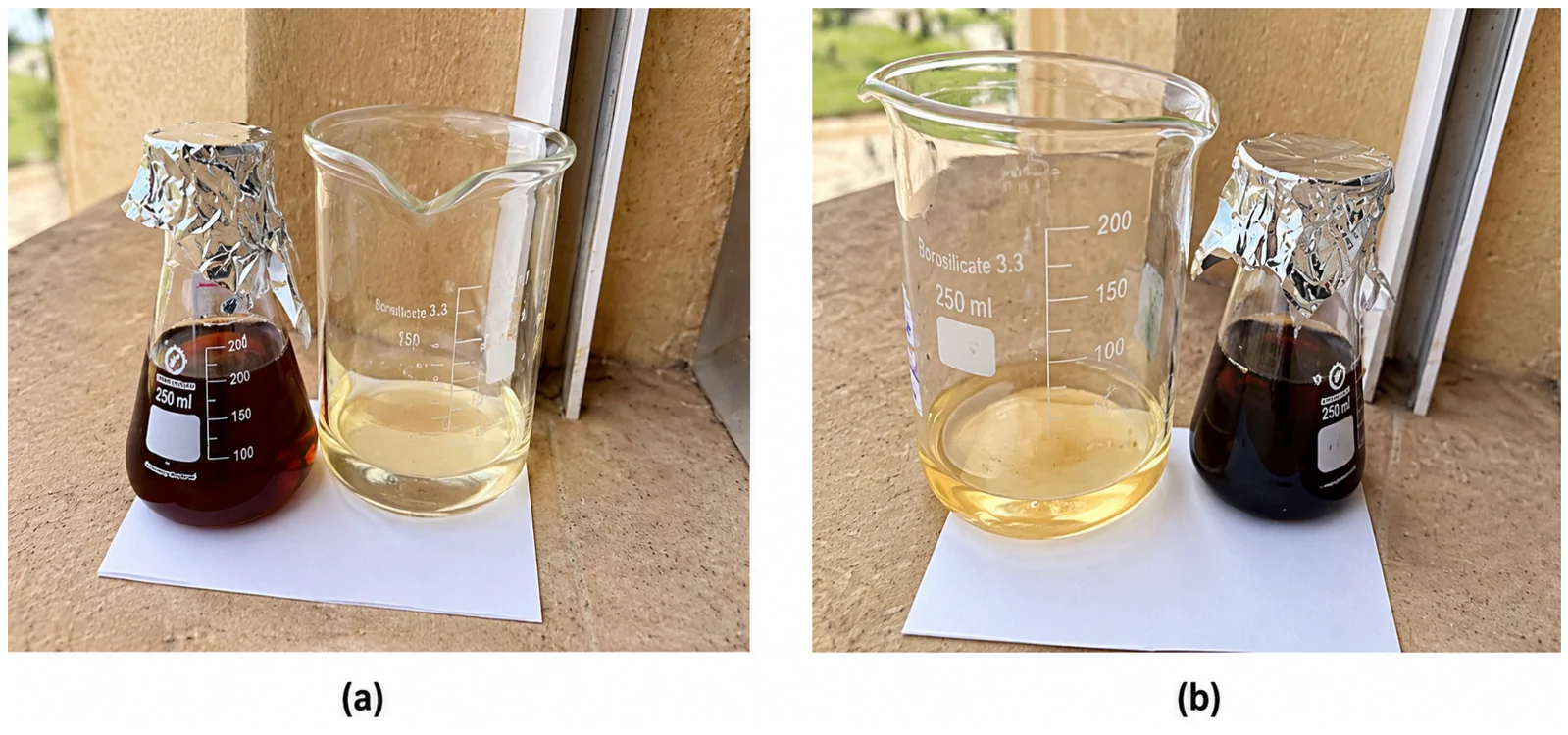
*Ziziphus* extract before (**a**) and after (**b**) the addition of Ag^+^.

The synthesis of AgNPs was first evidenced by a visible color shift after plant extracts were added to the silver nitrate solution. The mixture progressively turned from pale yellow to dark brown; a change typically linked to the reduction of Ag^+^ ions to metallic silver nanoparticles in plant extracts. This color development results from the interaction between silver ions and phytochemical constituents present in the plant extracts, including phenolics, flavonoids, terpenoids, and other reducing biomolecules (Figure 2 and Figure 3). The intensity of the brown coloration increased progressively with reaction time, suggesting the gradual reduction of silver ions and the formation of stable colloidal nanoparticles. Similar color changes were observed for both *Artemisia absinthium* and *Ziziphus vulgaris* extracts, indicating that the phytochemicals present in both plant systems effectively facilitated the reduction and stabilization of silver nanoparticles. These visual observations provided preliminary evidence of nanoparticle formation prior to further structural and morphological characterization using electron microscopy and spectroscopic techniques.

### 2.2. Transmission and Scanning Electron Microscopy

**Figure 4:**
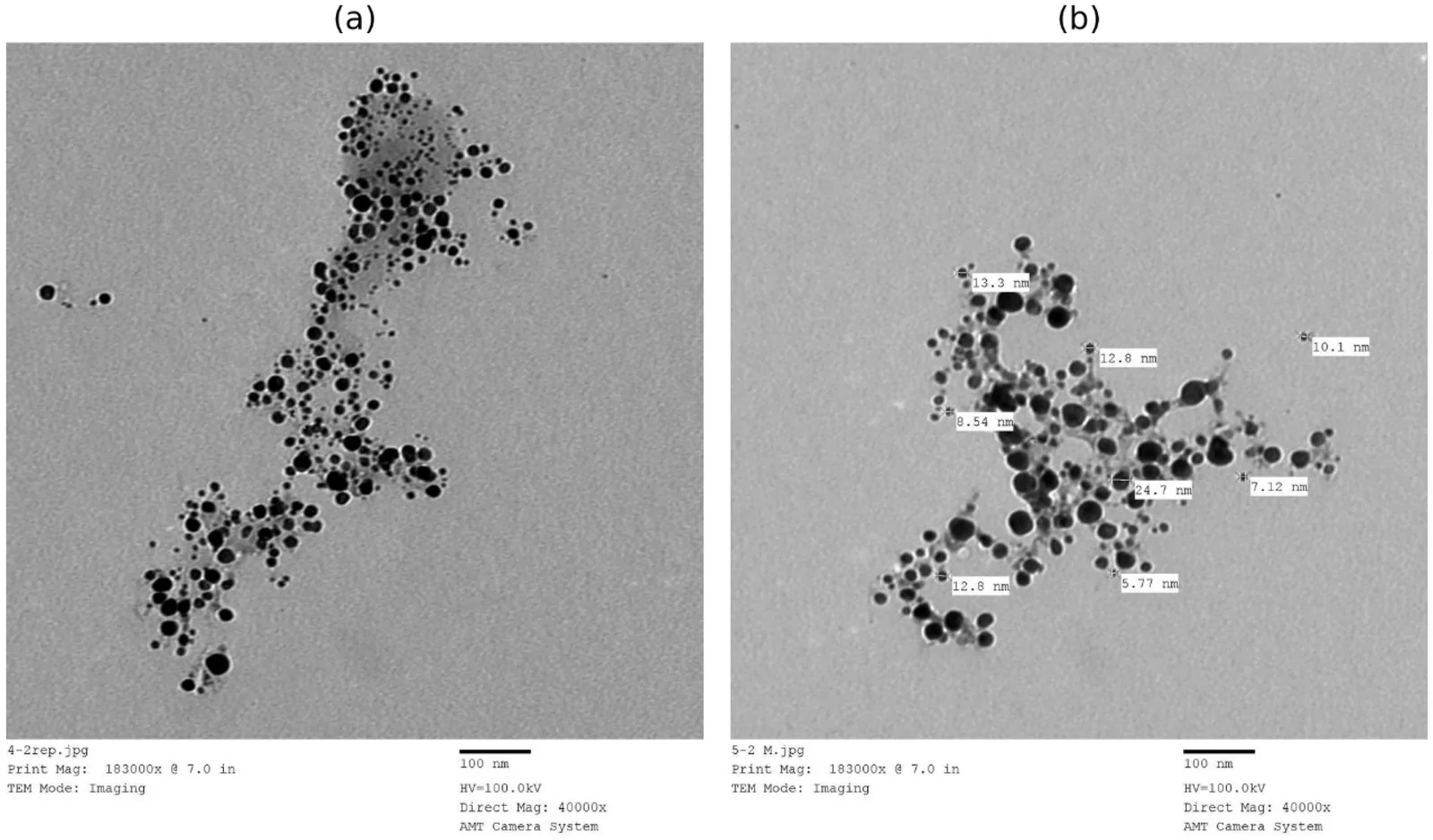
Transmission electron microscopy (TEM) images of *Artemisia* stem-derived nanoparticles: (**a**) representative micrographs illustrating plain nanoparticle morphology and aggregation; (**b**) representative micrographs showing selected particle-size measurements within the aggregates.

**Figure 5:**
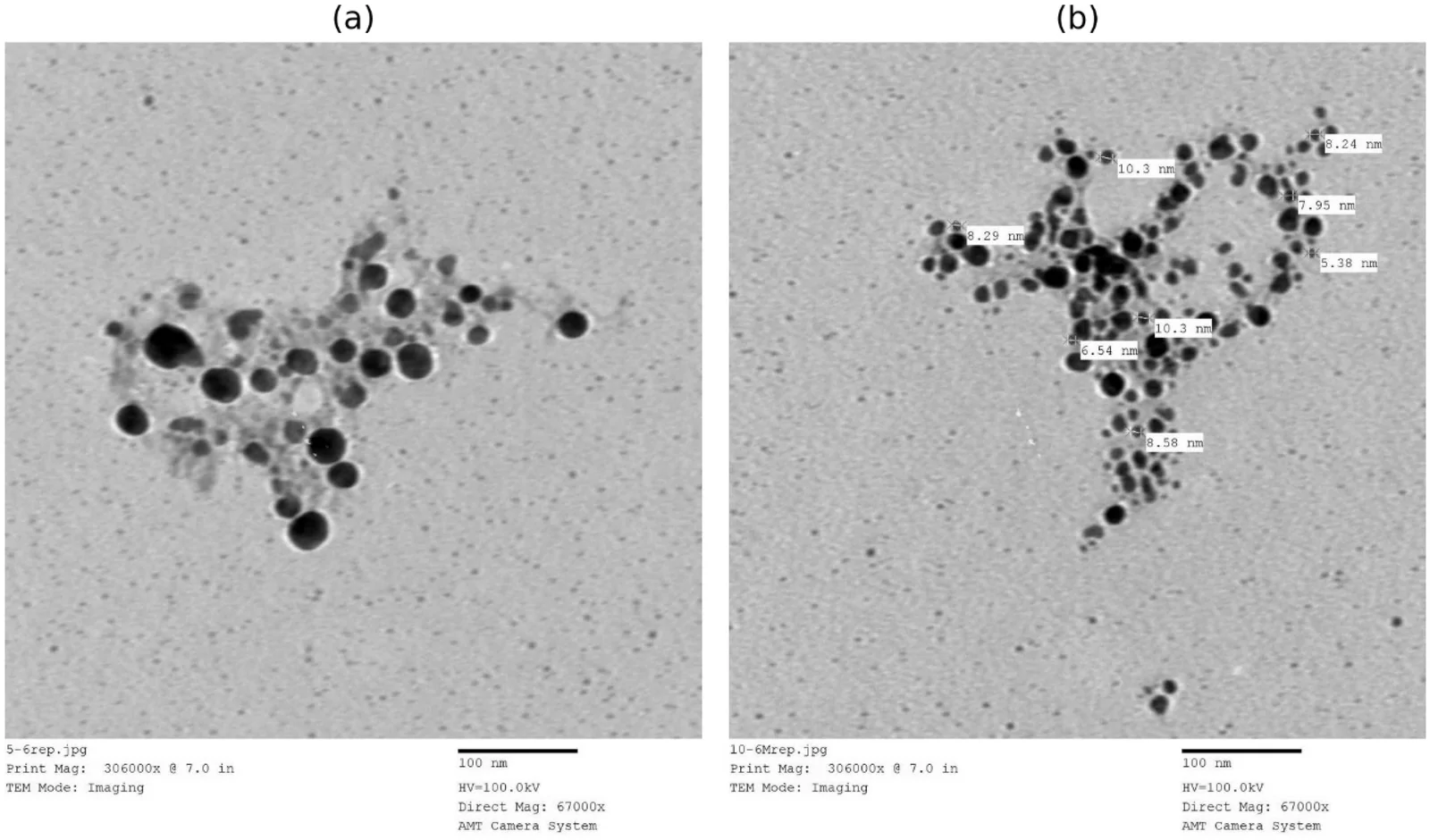
Transmission electron microscopy (TEM) images of *Ziziphus* stem-derived nanoparticles: (**a**) representative micrographs illustrating plain nanoparticle morphology and aggregation; (**b**) representative micrographs showing selected particle-size measurements within the aggregates.

**Figure 6:**
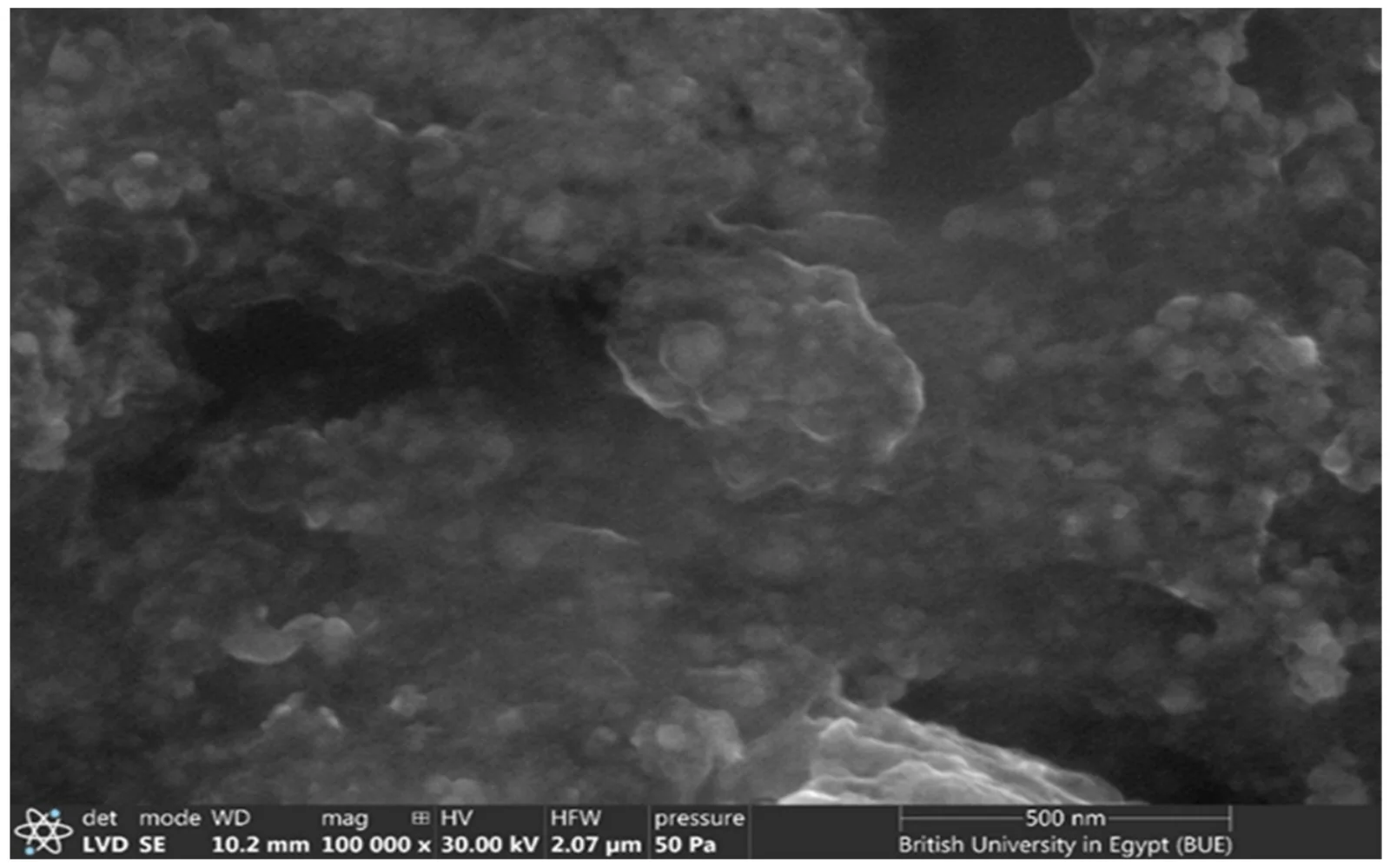
Scanning Electron Microscopy (SEM) analysis of *Artemisia* stem’s nanoparticles.

**Figure 7:**
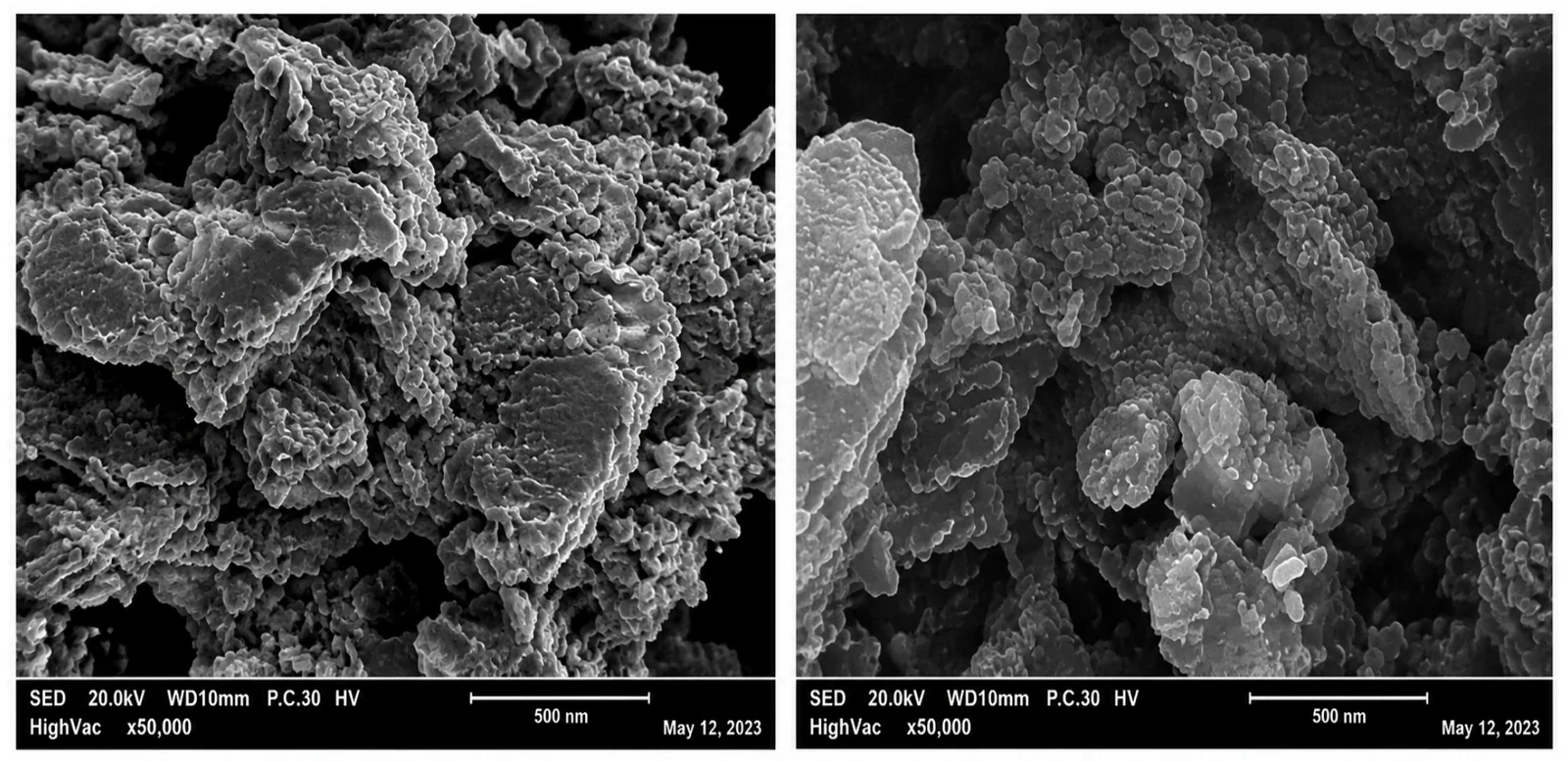
Scanning Electron Microscopy (SEM) analysis of *Ziziphus* stem’s nanoparticles. Resolution was improved using ChatGPT AI platform (GPT-6, OpenAI).

Transmission Electron Microscopy (TEM) analysis (Figure 4 and Figure 5) revealed predominantly spherical silver nanoparticles with narrow size distributions. The calculated average particle diameters were 16.8 ± 2.1 nm for *Artemisia* leaf AgNPs, 18.2 ± 2.3 nm for *Artemisia* stem AgNPs, 15.6 ± 2.0 nm for *Ziziphus* leaf AgNPs, and 17.4 ± 2.5 nm for *Ziziphus* stem AgNPs (*n* = 100 particles). In addition to the dominant spherical morphology, occasional pentagonal and triangular nanostructures were observed, suggesting polycrystalline growth behavior and anisotropic crystal development. The well-defined particle boundaries and contrast observed in TEM micrographs further support the formation of crystalline silver nanostructures, consistent with previously reported plant-mediated metallic AgNPs. Scanning Electron Microscopy (SEM) analysis (Figure 6 and Figure 7) further confirmed the formation of densely packed and uniformly distributed nanoparticles with minimal aggregation. The observed surface morphology indicates effective stabilization and capping by phytochemicals present in the plant extracts, which likely contributed to controlling particle growth and preventing agglomeration.

Furthermore, the synthesized nanoparticles retained their characteristic optical properties after storage at room temperature for at least 90 days, demonstrating excellent colloidal stability, as evidenced in the Supplementary Data (Figures S1–S4).

### 2.3. Fourier-Transform Infrared Spectroscopy

**Figure 8:**
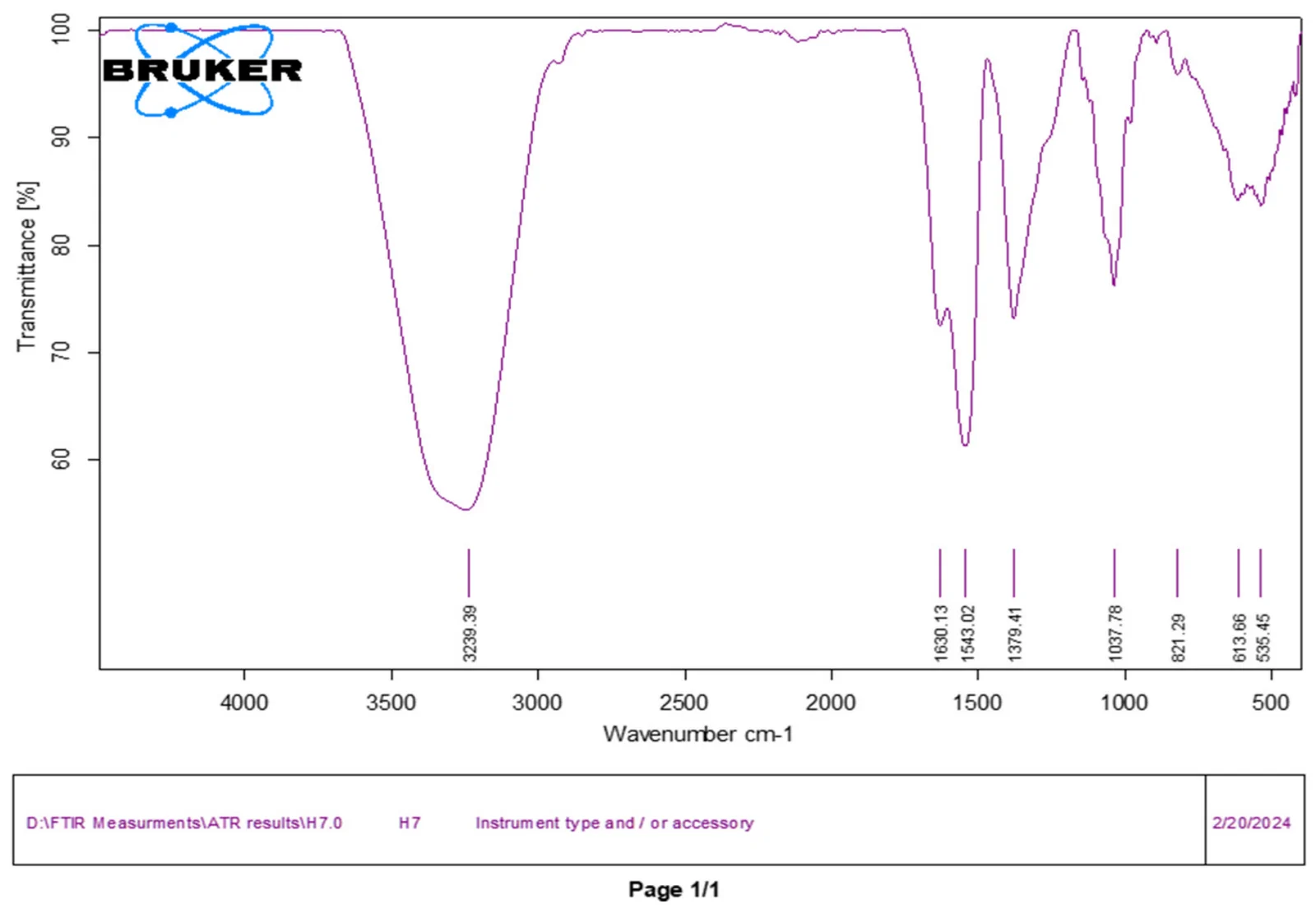
I.R Spectra of *Artemisia* stem’s nanoparticles.

**Figure 9:**
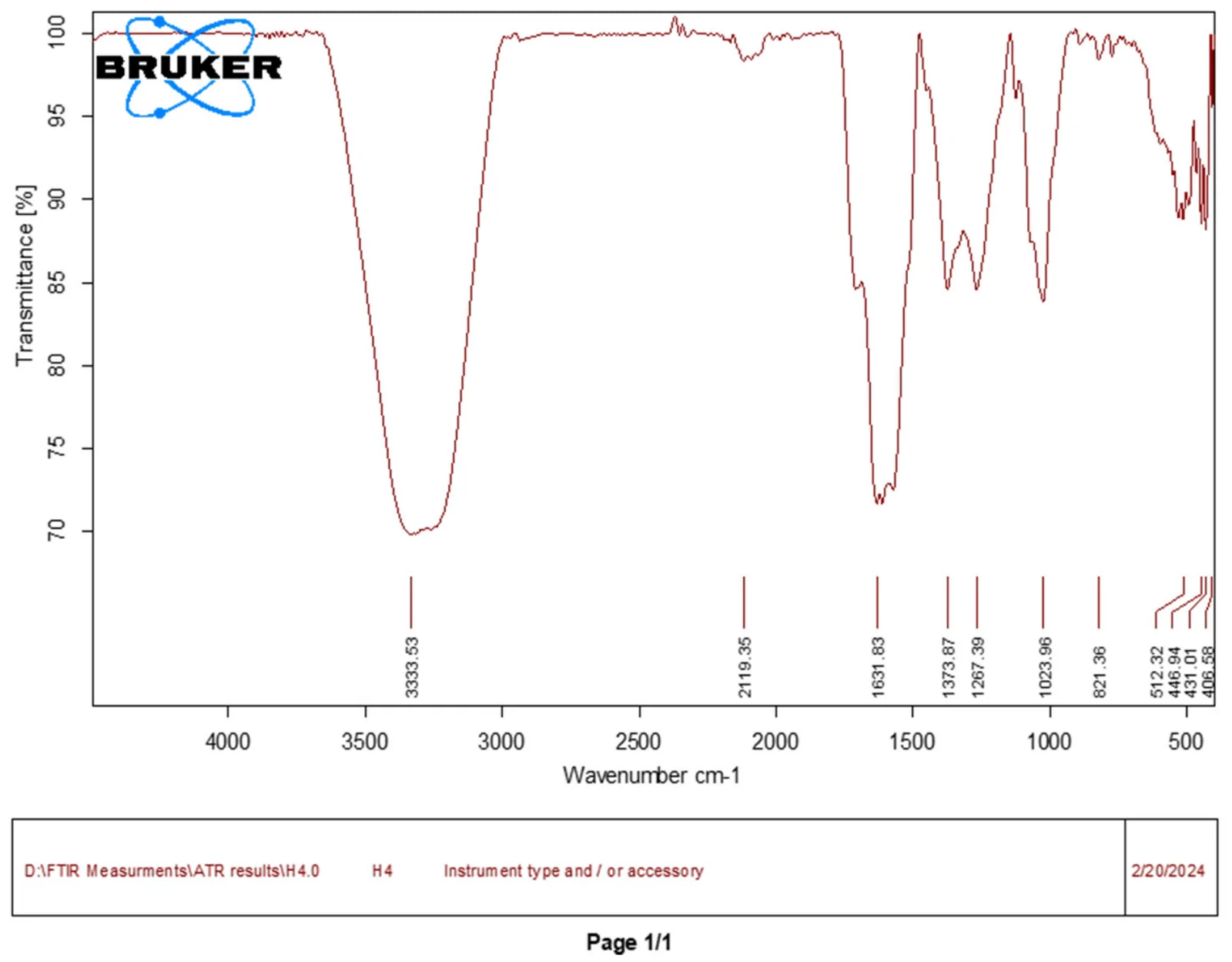
I.R Spectra of *Ziziphus* stem’s nanoparticles.

FTIR spectra revealed prominent absorption bands at 3339–3239 cm^−1^ (O–H stretching of phenolics and alcohols), 1630–1611 cm^−1^ (C=O stretching of carbonyl groups), and 1076–1014 cm^−1^ (C–O stretching of esters and polysaccharides), (Figure 8 and Figure 9). These functional groups are abundant in *Artemisia* and *Ziziphus* extracts and are responsible for reducing Ag^+^ to Ag^0^ and capping the nanoparticles to prevent aggregation. Minor shifts in peak positions between the two plant systems indicate differences in phytochemical composition and binding strength (Figure S5). In addition, several absorption bands were observed in the region below 1000 cm^−1^ (approximately 900–600 cm^−1^), which correspond to fingerprint-region vibrations such as C–H bending, aromatic ring deformation, and possible metal–oxygen (Ag–O) interactions between the silver nanoparticle surface and plant-derived capping molecules. These bands further support the coordination of phytochemical constituents with the nanoparticle surface and contribute to the stabilization of the biosynthesized AgNPs.

### 2.4. Dynamic Light Scattering (DLS)

The particle size distribution and hydrodynamic diameter of the synthesized AgNPs in all studied plant samples were estimated by dynamic light scattering (DLS). As shown in the intensity-based size distribution curves, all samples exhibited mean hydrodynamic diameters in the nanometric range (<100 nm). The Z-average (intensity-weighted mean hydrodynamic diameter) values for the AgNPs obtained from each plant extract are summarized in Table 1.

**Figure 10:**
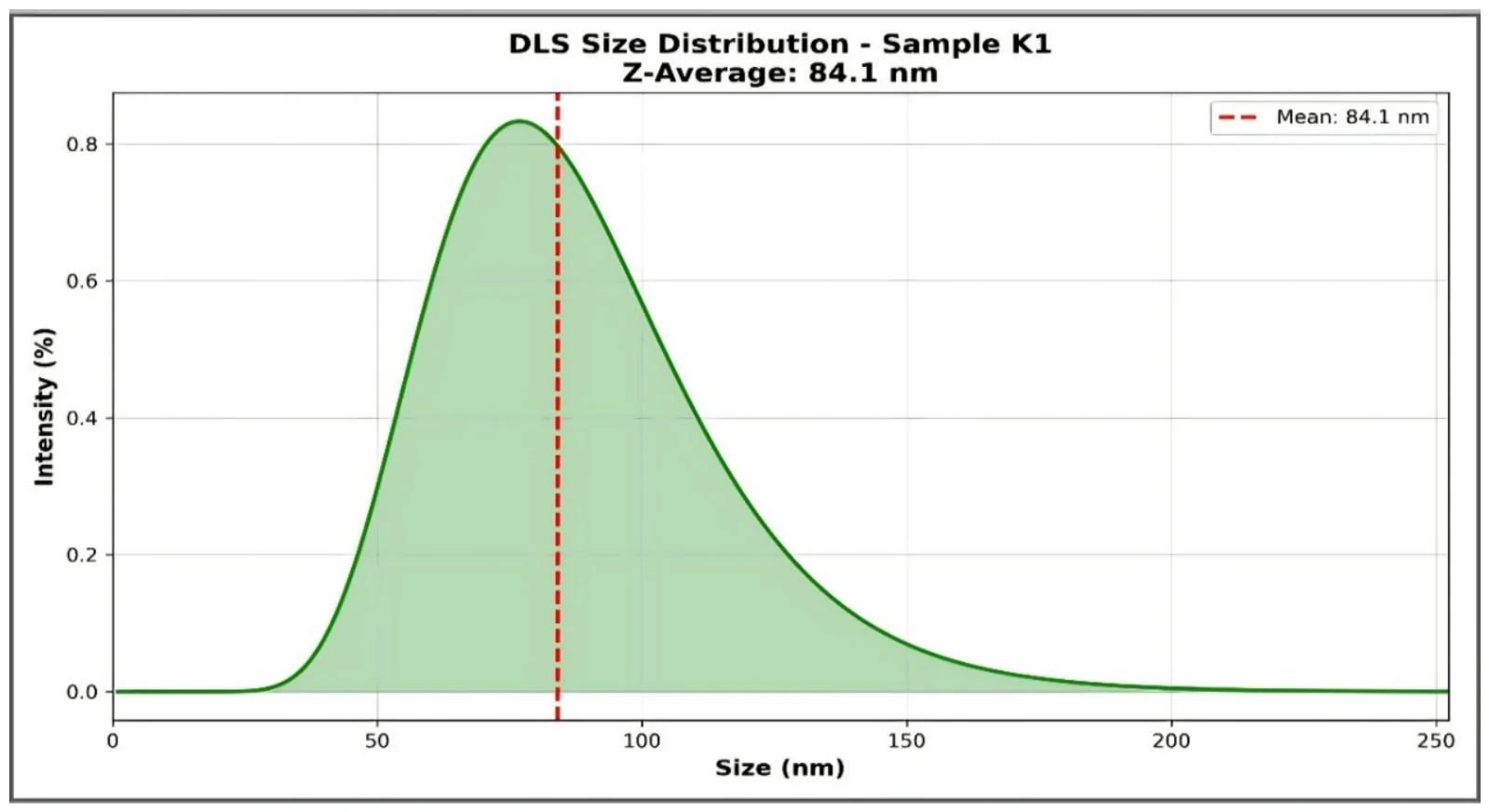
DLS of *Artemisia* leaves‘ nanoparticles.

All samples exhibited a monomodal size distribution with a relatively narrow peak, indicating a high degree of homogeneity in the synthesized nanoparticles (Figure 10 for *Artemisia* leaves; Figures S6–S8 for *Artemisia* stems, *Ziziphus* leaves, and *Ziziphus* stems, respectively).

### 2.5. Zeta Potential Analysis

The zeta potential distribution of the synthesized AgNPs was measured to evaluate their colloidal stability. Mean zeta potential values obtained from electrophoretic light scattering (ELS) analysis are summarized in Table 2.

**Figure 11:**
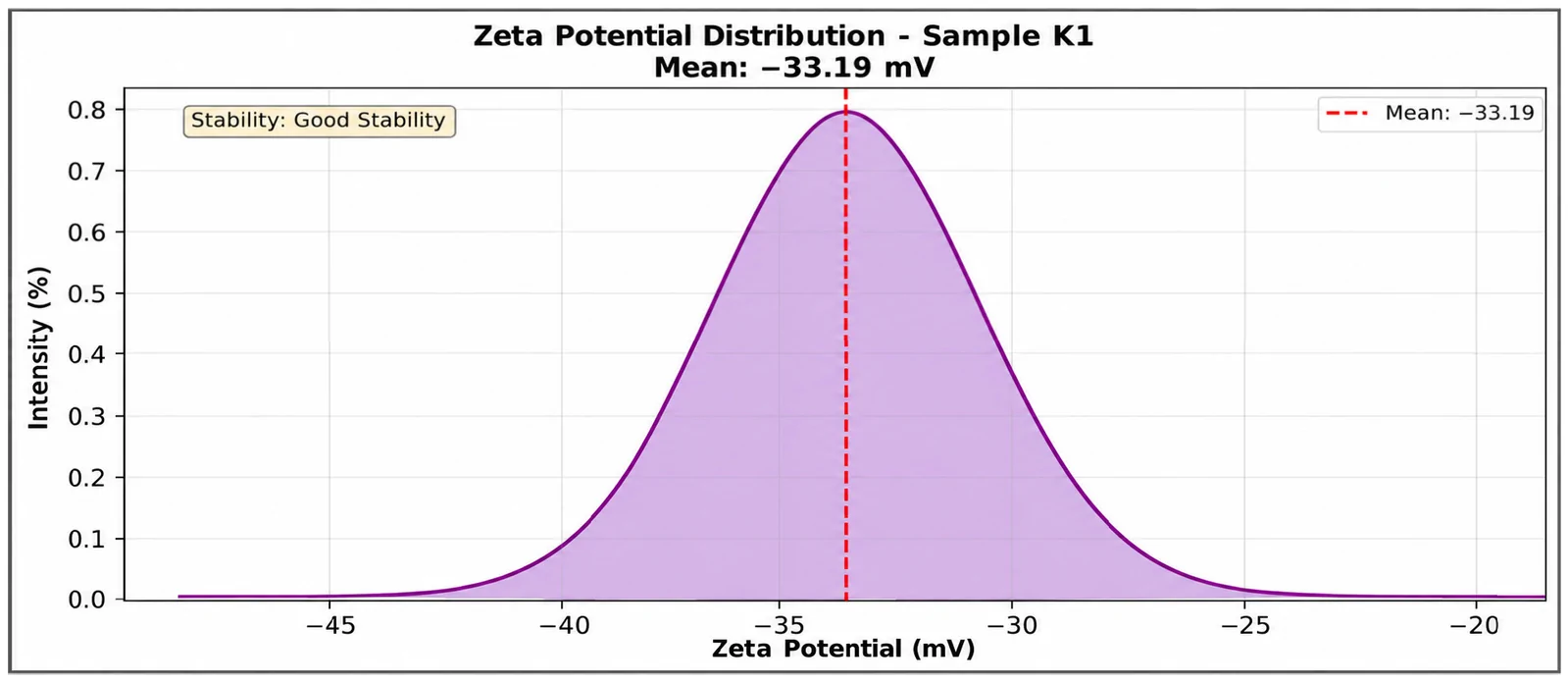
Zeta potential analysis of Ag NPs synthesized using *Artemisia* leaf extract.

Zeta potential is a key indicator of particle surface charge and colloidal stability. In general, zeta potential values greater than +30 mV or less than −30 mV denote a stable colloidal system, where strong electrostatic repulsion between particles prevents aggregation and flocculation. As shown in Figure 11 (*Artemisia* leaves) and Figures S9–S11 (*Artemisia* stems, *Ziziphus* leaves, and *Ziziphus* stems, respectively), all samples exhibited negative zeta potential values ranging from −33.19 to −34.40 mV. These substantially negative values indicate the presence of a robust electrical double layer around the nanoparticles, providing sufficient repulsive forces to overcome van der Waals attractive interactions. The progressively more negative zeta potential may reflect a higher surface density of ionizable functional groups and/or a more efficient stabilization by phytochemical capping agents. The narrow, bell-shaped zeta potential distributions further confirm a high degree of homogeneity in surface charge across all samples.

### 2.6. X-Ray Diffraction (XRD) Analysis

**Figure 12:**
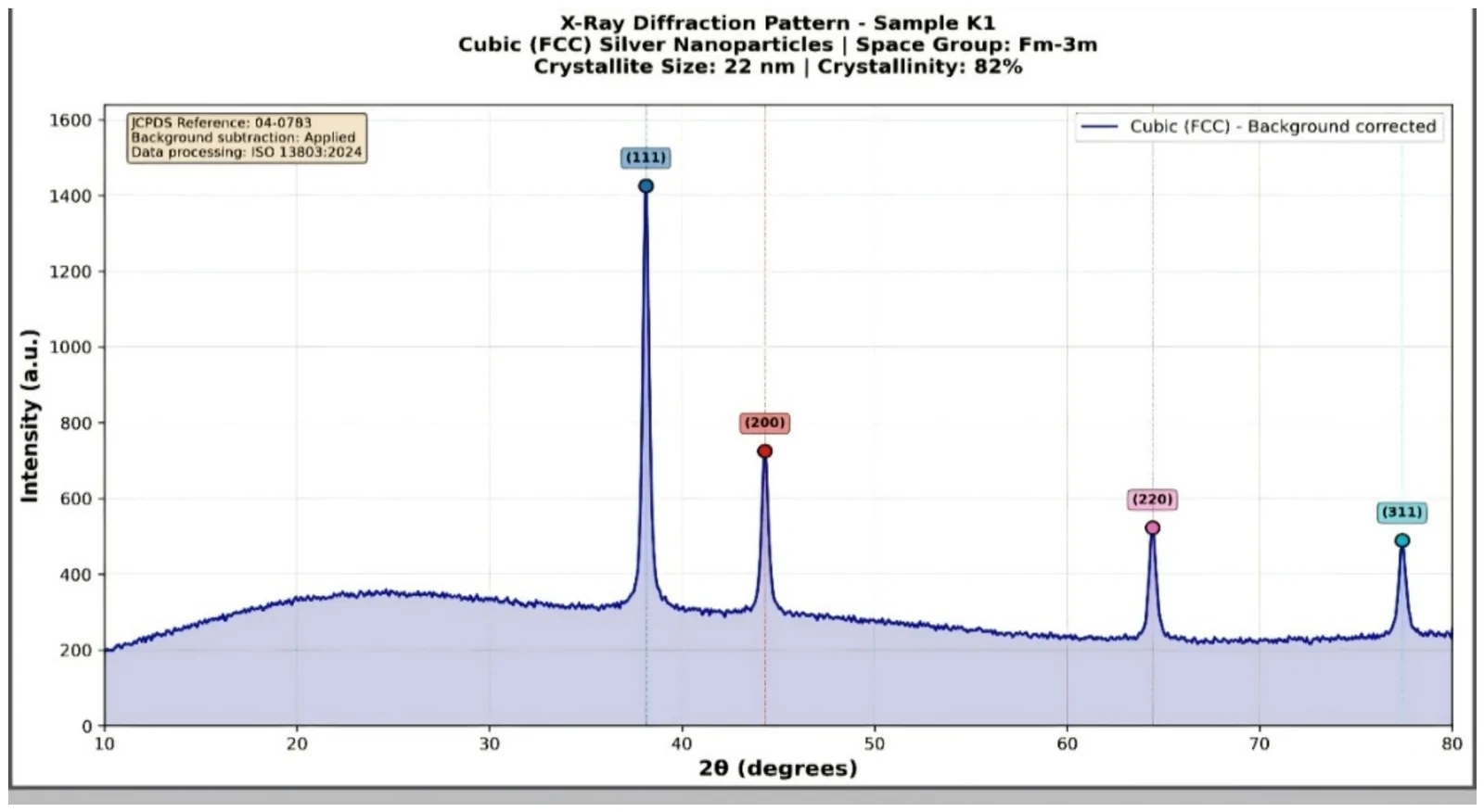
X-ray diffraction (XRD) pattern of AgNPs prepared using *Artemisia* leaf extract.

The crystallographic structure and phase purity of the synthesized silver nanoparticles (AgNPs) were evaluated by X-ray diffraction (XRD). All samples exhibited diffraction patterns consistent with a face-centered cubic (FCC) structure of metallic silver, in excellent agreement with JCPDS Card No. 04-0783. The high intensity of the (111) reflection relative to other peaks indicates a preferred orientation along this plane and high crystalline quality. As summarized in Table 3, the AgNPs prepared using *Artemisia* leaf extract showed the highest crystallinity (82%) and the largest crystallite size (22 nm), reflecting a more ordered lattice structure compared with the other samples. As shown in Figure 12 (*Artemisia* leaves) and Figures S12–S14 (*Artemisia* stems, *Ziziphus* leaves, and *Ziziphus* stems, respectively), the dominance of the (111) plane as the main diffraction peak indicates a lower surface energy configuration, which is thermodynamically favorable for silver at the nanoscale.

### 2.7. Energy Dispersive X-Ray (EDX) Spectroscopy Analysis

**Figure 13:**
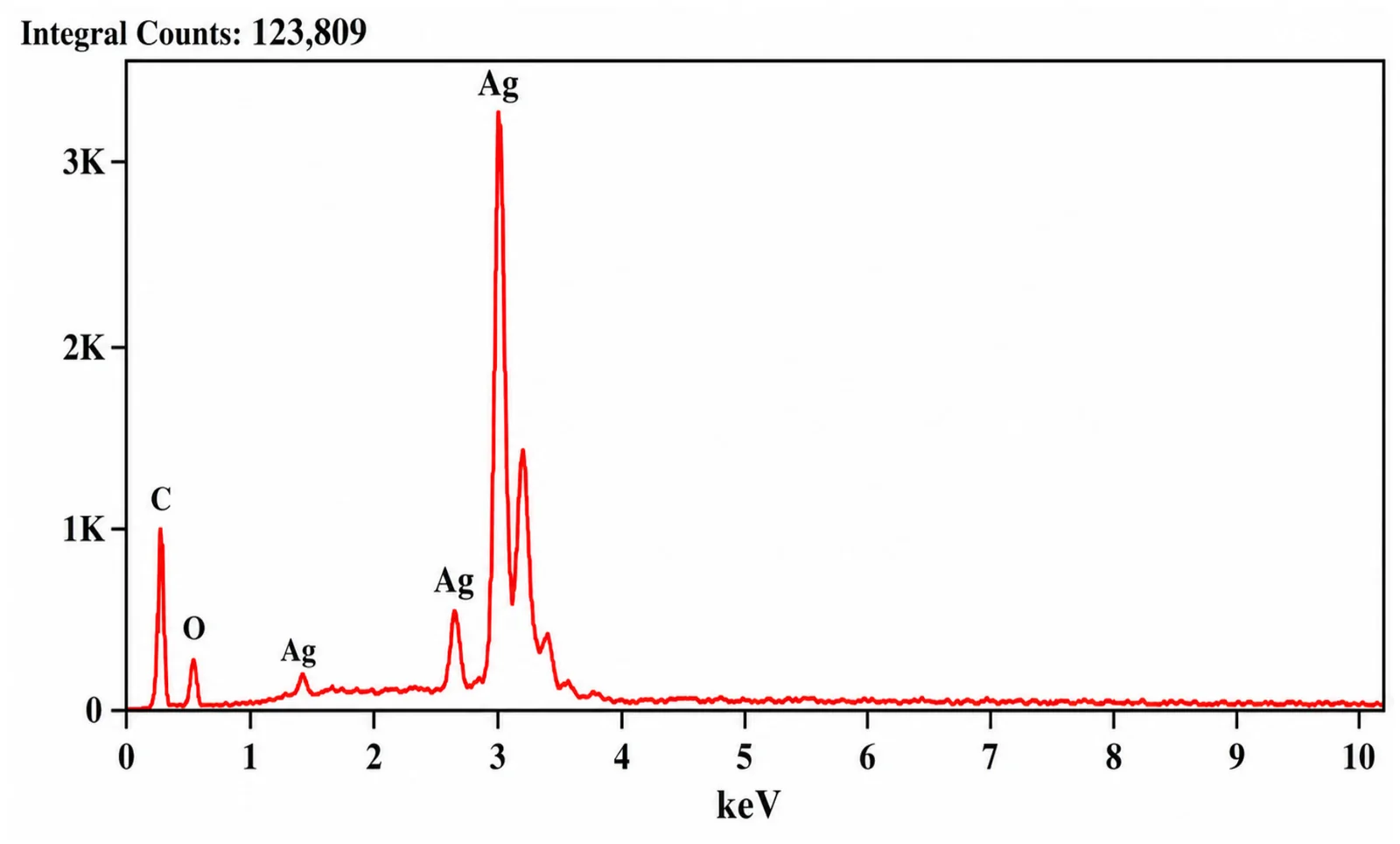
EDX spectrum of AgNPs prepared using *Artemisia* leaf extract.

The elemental composition of the synthesized AgNPs was investigated by energy-dispersive X-ray (EDX) spectroscopy. The EDX spectra of all samples confirmed silver (Ag) as the major constituent, with characteristic Ag peaks clearly observed. As shown in Figure 13 (*Artemisia* leaves) and Figures S15–S17 (*Artemisia* stems, *Ziziphus* leaves, and *Ziziphus* stems, respectively), all samples exhibited high Ag content, indicating the efficient reduction of Ag^+^ to metallic Ag and successful nanoparticle formation. The quantitative EDX results are summarized in Table 4.

## 3. Discussion

Collectively, the results of the chemical characterization part of this study confirm the successful green synthesis of stable, nearly spherical AgNPs using *Artemisia absinthium* and *Ziziphus vulgaris* leaf and stem extracts, with particle sizes of approximately 15–22 nm (TEM/SEM) and hydrodynamic diameters < 90 nm (DLS). FTIR spectra identified phenolic, carbonyl, and polysaccharide functional groups as key reducing and capping agents, while XRD patterns confirmed a pure face-centered cubic (FCC) metallic silver phase with high crystallinity, particularly for *Artemisia* leaf-derived AgNPs. EDX analysis verified silver as the dominant elemental constituent (Ag Lα peak at ~3 keV), and strongly negative zeta potentials (−33 to −34 mV) indicated robust colloidal stability arising from phytochemical surface capping.

In addition, the present study primarily focuses on evaluating the biological activities of the biosynthesized AgNPs. Detailed mechanistic investigations, such as reactive oxygen species generation, apoptosis pathway analysis, or inflammatory mediator studies, were not conducted and will be explored in future work to better understand the underlying mechanisms of action. Previous studies have suggested that silver nanoparticles exert antibacterial activity through disruption of bacterial cell membranes and oxidative stress induction, while their anticancer effects are often associated with apoptosis induction and mitochondrial dysfunction.

### 3.1. Antibacterial Activity

#### 3.1.1. Assessment of the Antibacterial Activity Using the Agar Disk Diffusion Approach

The biosynthesized AgNPs displayed strong antibacterial effects against both *Escherichia coli* (Gram-negative) and *Staphylococcus aureus* (Gram-positive). For *E. coli*, inhibition zones measured 18.4 ± 0.6 mm and 17.1 ± 0.5 mm for *Artemisia* leaf and stem nanoparticles, respectively, whereas *Ziziphus* leaf and stem nanoparticles produced slightly larger zones of 20.1 ± 0.7 mm and 19.6 ± 0.6 mm. Against *S. aureus*, *Artemisia* leaf and stem AgNPs achieved inhibition zones of 16.2 ± 0.5 mm and 15.7 ± 0.4 mm, while *Ziziphus* leaf and stem AgNPs produced 17.5 ± 0.4 mm and 17.1 ± 0.3 mm, respectively, Table 5 and Table 6 and Tables S1–S6. The consistently higher inhibition produced by *Ziziphus* nanoparticles reflects subtle differences in surface chemistry and capping molecules, most likely related to the higher content of saponins and triterpenoids in *Ziziphus* extracts, (Figures S18–S21). The small particle sizes of 15–22 nm also promote efficient penetration through bacterial cell walls, (Figures S22–S25). These findings agree with previous reports that plant-capped AgNPs disrupt bacterial membrane, produce reactive oxygen species (ROS), and affect DNA replication [21,22,23]. The antibacterial activity was evaluated using the agar disk diffusion method as a preliminary screening tool.

#### 3.1.2. Determination of Minimum Inhibitory Concentration (MIC) and Minimum Bactericidal Concentration (MBC)

The observed inhibition zones in the agar well diffusion assay indicate that the biosynthesized AgNPs possess significant antibacterial activity against both Gram-positive (*Staphylococcus aureus* ATCC 6538) and Gram-negative (*Escherichia coli* ATCC 8739) bacteria, consistent with previously reported antimicrobial properties of plant-mediated AgNPs. However, the agar well diffusion method provides only a preliminary, semi-quantitative evaluation of antibacterial activity and can be influenced by nanoparticle diffusion behavior in the agar medium. Therefore, more precise quantitative assessments using minimum inhibitory concentration (MIC) and minimum bactericidal concentration (MBC) assays were performed. A summary of the MIC and MBC procedures for the biosynthesized nanoparticles is displayed in Supplementary Section S1.

MIC and MBC values for AgNPs synthesized from *Artemisia* leaves, *Artemisia* stems, *Ziziphus* leaves, and *Ziziphus* stems are summarized in Table 7 and the detailed results are displayed in Supplementary Table S7. Against *S. aureus*, MIC values ranged from 15.62 to 125 µg mL^−1^, with the lowest MIC observed for *Ziziphus* stem-mediated AgNPs (15.62 µg mL^−1^), followed by *Ziziphus* leaf-mediated AgNPs (31.25 µg mL^−1^). *Artemisia* leaf- and stem-mediated AgNPs exhibited higher MICs of 62.2 and 125 µg/mL, respectively. A similar trend was observed against *E. coli*, where MIC values ranged from 15.62 to 125 µg mL^−1^; again, *Ziziphus* stem-mediated AgNPs showed the strongest inhibitory activity (MIC = 15.62 µg mL^−1^), whereas *Artemisia* stem-mediated AgNPs were the least active (MIC = 125 µg mL^−1^).

Notably, AgNPs synthesized using *Ziziphus* extracts (particularly stems) were more potent than those synthesized using *Artemisia* extracts, as evidenced by consistently lower MIC and MBC values against both *S. aureus* and *E. coli*. among all samples. These results confirm that the biosynthesized AgNPs not only inhibit bacterial growth but also exert a bactericidal effect at concentrations relatively close to their MICs, supporting their potential application as effective antibacterial agents.

### 3.2. Anti-Inflammatory Activity

The membrane-stabilizing potential of the nanoparticles was evaluated by their ability to inhibit hypotonicity-induced hemolysis of human red blood cells. Both *Artemisia* and *Ziziphus* AgNPs exhibited concentration-dependent inhibition, with maximal effects observed at the highest tested concentration of 1000 µg mL^−1^. At this level, *Artemisia* leaf and stem nanoparticles inhibited hemolysis by 87.0 ± 1.3 % and 85.2 ± 1.4 %, respectively (Table 8 and Table S8), whereas *Ziziphus* leaf and stem nanoparticles achieved 91.0 ± 1.2 % and 89.5 ± 1.5 % inhibition (Table 9 and Table S9). The stronger effect of *Ziziphus* AgNPs parallels their superior antibacterial performance. Stabilization of the erythrocyte membrane is attributed to interactions of AgNPs with membrane proteins and lipids, which reduce osmotic stress and oxidative damage [24], (Figures S26–S29). These results suggest that plant-mediated AgNPs can protect cells against inflammation-induced lysis and may be promising candidates for anti-inflammatory therapy.

### 3.3. Cytotoxicity Toward MCF-7 Breast Cancer, HepG2 Liver Cancer, and A549 Lung Carcinoma Cells

The MTT assay demonstrated a clear dose-dependent decrease in viability of MCF-7 cells and other cells exposed to the green-synthesized AgNPs. Cytotoxic activity was evaluated against MCF-7 breast cancer cells (Table 10), and against HepG2 human liver cancer cells and A549 lung carcinoma cells (Supplementary Tables S10 and S11, respectively), using the MTT assay. Half-maximal inhibitory concentrations (IC_50_) were calculated as 19.6 ± 1.2 µg mL^−1^ for *Artemisia* leaf nanoparticles, 20.2 ± 1.4 µg mL^−1^ for *Artemisia* stem nanoparticles, 21.3 ± 1.5 µg mL^−1^ for *Ziziphus* leaf nanoparticles, and 21.0 ± 1.6 µg mL^−1^ for *Ziziphus* stem nanoparticles, (Figures S30–S35). Microscopic examination revealed hallmark features of apoptosis including cell shrinkage, membrane blebbing, and nuclear condensation particularly at concentrations near the IC50, (Figures S36–S44). The slightly higher potency of *Artemisia* AgNPs may arise from synergistic effects of flavonoids and sesquiterpene lactones, which can enhance ROS production and trigger mitochondrial pathways of apoptosis [25,26,27], Table 10. Previous studies show that AgNPs disrupt mitochondrial membrane potential, activate caspase-3, and cause DNA fragmentation [28,29]; our findings are consistent with these mechanisms and confirm the anticancer potential of both plant-derived nanoparticles, Tables S10 and S11.

A summary of the half-maximal inhibitory concentration (IC_50_) values for the biosynthesized nanoparticles in DMSO against the non-cancerous WI-38 cell line is presented in Table 11, while the detailed experimental data, including optical density readings, viability percentages, toxicity percentages, and calculated IC_50_ values, are provided in Supplementary Table S12 and Supplementary Figures S45–S49.

Importantly, the IC_50_ values against the non-cancerous WI-38 lung fibroblast cell line were markedly higher, ranging from 208.71 µg mL^−1^ to >250 µg mL^−1^ (Table 11), indicating substantially lower cytotoxicity toward normal cells. This pronounced selectivity, with IC_50_ values for cancer cells approximately 10- to 12-fold lower than those for WI-38 cells, suggests that the biosynthesized AgNPs preferentially target malignant cells while sparing normal cells at therapeutically relevant concentrations. Such differential cytotoxicity supports the potential of these nanoparticles as selective anticancer agents with an acceptable safety margin for normal human cells.

### 3.4. Scratch Wound Healing Assessment

**Figure 14:**
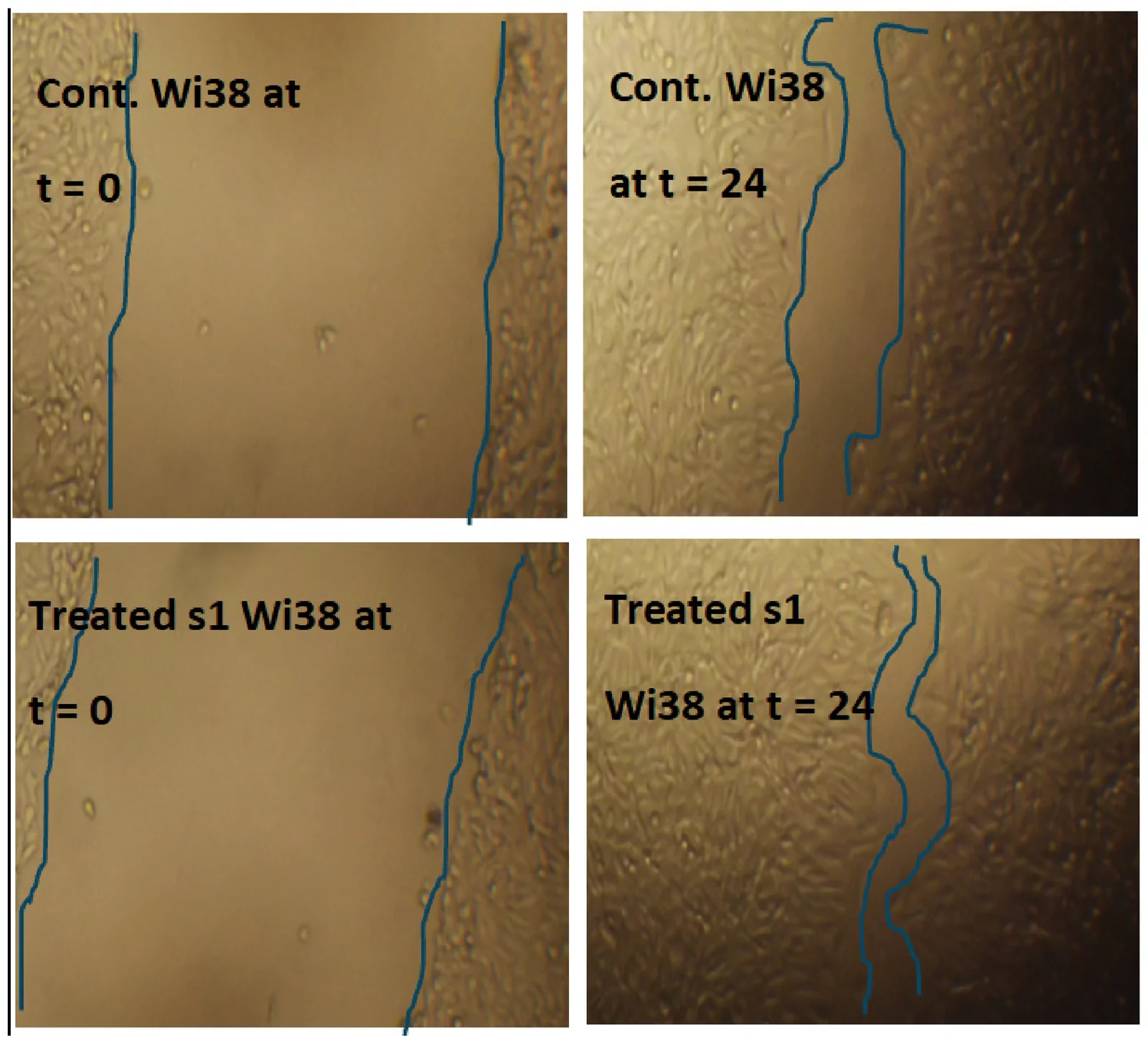
Scratch wound healing assay of Wi-38 fibroblasts treated with Sample 1 (*Artemisia* leaves) for 24 h.

To further evaluate the biological impact of the biosynthesized nanoparticles on normal cells, a scratch wound healing assay was performed using the non-cancerous WI-38 fibroblast cell line. This two-dimensional migration assay quantitatively assesses cell motility and cell–cell interactions by measuring the rate of wound closure over time. Four nanoparticle samples (coded 1–4), corresponding to *Artemisia* leaf, *Artemisia* stem, *Ziziphus* leaf, and *Ziziphus* stem AgNPs, were dissolved and tested in DMSO at non-cytotoxic concentrations. Calculations for the scratch wound healing assay are provided in Supplementary Section S2. Detailed microscopic observations and raw measurements of wound area are presented in Supplementary Table S13, while Table 12 summarizes the calculated wound-closure percentages and corresponding wound areas (µm^2^) for each sample relative to the untreated control. Scratch wound healing results for Sample 1 (*Artemisia* leaves) are displayed in Figure 14, while results for samples 2–4 are shown in Supplementary Figures S50–S52.

The scratch wound healing assay showed a wound closure of 77.6% for the untreated WI-38 control, whereas all four plant-mediated nanoparticle samples exhibited enhanced wound healing, with closure percentages reaching up to 93.4% in sample 3 (*Ziziphus* leaves). These findings indicate that the biosynthesized nanoparticles are not only biocompatible with normal human fibroblasts but also actively promote cell migration and wound repair, suggesting their potential as promising topical wound-healing agents for skin applications. This pro-healing effect is consistent with their demonstrated antimicrobial activity, supporting their dual functionality as both antibacterial and wound-healing materials. It should be noted that the therapeutic wound healing activities are preliminary, as they are based on in vitro assays that require further in vivo confirmation.

### 3.5. Comparative Analysis of Plant Sources

Despite identical synthesis conditions, measurable differences in biological performance were observed between the two plant systems. *Ziziphus* AgNPs displayed slightly stronger antibacterial, anti-inflammatory, and wound healing activities, whereas *Artemisia* AgNPs showed marginally greater cytotoxicity toward cancer cells. These contrasting patterns likely reflect differences in phytochemical composition: *Artemisia* extracts contain abundant flavonoids and sesquiterpene lactones that favor ROS-mediated apoptosis, whereas *Ziziphus* extracts are rich in saponins and triterpenoids that interact strongly with bacterial membranes and erythrocyte proteins.

Such plant-specific effects highlight the importance of selecting appropriate botanical sources when tailoring the properties of green-synthesized nanoparticles. These findings further support the hypothesis that plant-specific phytochemical profiles play a decisive role in directing nanoparticle formation and biological performance, even when synthesis conditions are fully standardized.

### 3.6. Mechanistic Insights into Biological Activities

The multiple biological activities of silver nanoparticles arise from a combination of physical interactions and chemical reactivity. In antibacterial systems, AgNPs attach to bacterial cell walls and membranes through electrostatic attraction, causing structural damage and increasing membrane permeability. Once internalized, silver ions released from the nanoparticle surface interact with DNA and intracellular proteins, disrupting metabolic pathways and inhibiting replication. The generation of ROS further exacerbates oxidative stress, leading to cell death [30,31,32]. The slightly enhanced antibacterial effect of *Ziziphus* AgNPs may be attributed to the stronger binding of saponin-rich capping agents, which promote stable release of silver ions and facilitate interaction with bacterial membranes. The anti-inflammatory activity observed in the hypotonicity-induced hemolysis model is likely related to the capability of AgNPs to stabilize erythrocyte membranes. Nanoparticles can interact with phospholipid bilayers, reducing the influx of water into cells and preventing lysis. Additionally, phenolic and flavonoid compounds adsorbed on the nanoparticle surface may scavenge free radicals and inhibit the release of pro-inflammatory mediators [33].

Cytotoxicity toward MCF-7 breast cancer cells is primarily mediated through the initiation of apoptosis. ROS generation disrupts mitochondrial membrane potential, activates caspase cascades, and causes fragmentation of nuclear DNA [34,35]. The slightly lower IC_50_ values of *Artemisia*-derived nanoparticles suggest that flavonoids and sesquiterpene lactones enhance ROS formation, thereby promoting mitochondrial dysfunction and apoptotic signaling.

The enhanced wound closure likely arises from sub-cytotoxic Ag^+^ release and phytochemical capping that together generate mild ROS signaling to activate fibroblast migration and proliferation pathways. Saponin and polyphenol compounds from *Ziziphus* extracts stabilize ion release and buffer oxidative stress, promoting lamellipodia formation and focal-adhesion turnover required for efficient scratch closure. This coordinated modulation of redox signaling, cytoskeletal dynamics, and biocompatibility underpins the dual antibacterial and pro-healing functionality of the biosynthesized nanoparticles [36,37].

This study provides one of the few direct comparative evaluations of AgNPs synthesized from two phytochemically distinct medicinal plants using identical experimental conditions. Most previous reports have focused on single-plant systems, making it difficult to separate the effects of plant chemistry from differences in synthesis parameters [38,39,40,41]. By standardizing silver nitrate concentration, plant-to-precursor ratios, and reaction conditions, we were able to attribute differences in nanoparticle properties directly to inherent phytochemical profiles. The finding that *Ziziphus* extracts produce slightly smaller, more antibacterial nanoparticles, while *Artemisia* extracts generate particles with stronger anticancer activity, underscores the importance of plant selection in designing application-specific nanomaterials.

In the present work, the colloidal stability of the biosynthesized silver nanoparticles was monitored over a 90-day storage period under controlled conditions (room temperature, protected from light). Stability was evaluated through periodic visual observation for signs of precipitation or color alteration, together with UV–Visible spectroscopic analysis of the surface plasmon resonance (SPR) band. Throughout the monitoring period, no visible sedimentation or significant change in solution color was observed. DLS revealed that all samples displayed a monomodal size distribution, confirming a high degree of homogeneity in the biosynthesized silver nanoparticles. Zeta potential measurements showed consistently negative values ranging from −33.19 to −34.40 mV, indicating a robust electrical double layer that generates sufficient electrostatic repulsion to counteract van der Waals attraction and ensure good colloidal stability. Many earlier studies reported particle aggregation within weeks when stored at ambient temperature [42,43]. The stability achieved here is attributed to the strong capping action of plant-derived phenolics and polysaccharides, which form a protective layer on the nanoparticle surface. Several plant systems have been explored for AgNP synthesis, including Azadirachta indica (neem), Withania somnifera (ashwagandha), and Origanum vulgare (oregano).

Neem extracts typically yield particles of 20–30 nm with excellent antibacterial activity but moderate anticancer effects [44]. Ashwagandha-derived nanoparticles have demonstrated strong cytotoxicity against HeLa and MCF-7 cells with IC_50_ values in the range of 25–35 µg mL^−1^ [45]. Oregano-based synthesis produces stable nanoparticles with particle sizes of 25–40 nm and robust antifungal properties [46]. Compared with these reports, the current method achieved smaller particle sizes (15–22 nm) and lower IC_50_ values (≈19–21 µg mL^−1^) using simple aqueous extracts and room-temperature conditions. The combination of small size, high stability, and broad-spectrum biological activity highlights the competitiveness of *Artemisia* and *Ziziphus* as green nano-factories.

Furthermore, many earlier methods relied on extended reaction times of 12–24 h or required elevated temperatures to achieve complete reduction [47,48]. In contrast, the present synthesis proceeded rapidly at ambient conditions, with visible color change occurring within 15–20 min and full reduction confirmed within 2 h. Such operational simplicity reduces energy consumption and enhances scalability for industrial production. Although UV–Visible spectroscopy and electron microscopy provide strong evidence for nanoscale metallic silver formation, the XRD analysis was further performed. The results showed that all samples exhibited a face-centered cubic (FCC) structure of metallic silver, with the high intensity of the (111) peak, indicating a preferred orientation along this plane and high crystalline quality. The EDX spectra of all samples confirmed silver (Ag) as the major constituent, indicating efficient reduction of Ag^+^ to metallic Ag and successful nanoparticle formation. Quantitative nanoparticle yield and silver conversion efficiency were determined in the present comparative investigation; similar charge intensities and particle size distributions under standardized synthesis conditions suggest comparable reduction efficiency between plant systems.

### 3.7. Limitations, Practical Implications and Future Perspectives

One limitation of this study is the absence of direct photochemical characterization, including total phenolic content (TPC), total flavonoid content (TFC), or HPLC-based chromatographic profiling of the extracts. To address this gap, we relied on previously published chromatographic investigations of the two studied plants to interpret the relationship between the extract nanocompositions and the assessed pharmacological activities. The absence of voucher specimen deposition represents another limitation of this botanical study, although plant identification was carried out using standard botanical descriptions from the literature.

The multifunctional biological performance of these plant-mediated silver nanoparticles highlights their potential applicability in advanced wound dressings, antimicrobial surface coatings, and targeted anticancer formulations. The combined antibacterial, anti-inflammatory, wound healing and cytotoxic activities observed in this study suggest that green-synthesized AgNPs derived from *Artemisia absinthium* and *Ziziphus vulgaris* may offer promising alternatives to chemically synthesized nanomaterials in biomedical applications. It should be noted that all the investigated therapeutic and biological activities are preliminary as they are based on in vitro assays that require further in vivo confirmation. Prior to clinical translation, comprehensive in vivo toxicity evaluation, biodistribution analysis, and pharmacokinetic profiling will be essential in order to establish safety and therapeutic windows. Furthermore, exploration of synergistic nanoformulations combining nanoparticles derived from different plant parts may provide enhanced biological performance through complementary phytochemical capping profiles. The optimization of extraction parameters, including solvent polarity, extraction time, and plant part selection, may further improve nanoparticle yield and functional efficiency.

## 4. Materials and Methods

### 4.1. Material Plant and Preparation of Extracts

Fresh leaves and stems of *Artemisia absinthium* and *Ziziphus vulgaris* were collected during the spring season from Al-Gabal Al-Akhdar, Libya. The plant materials were identified based on their morphological characteristics using standard botanical descriptions reported in the literature. Although formal taxonomic authentication by a qualified botanist and the deposition of voucher specimens in a recognized herbarium were not performed at the time of collection, the identification was conducted with careful reference to established taxonomic keys for both species. Samples were washed thoroughly with distilled water to remove surface contaminants, shade-dried at room temperature, and ground to a fine powder. Dried plant materials (leaves and stems) were finely powdered, and 5 g of each sample was extracted with 200 mL of distilled water (1:40 *w*/*v*). The mixture was heated at 70–80 °C for 30 min under continuous stirring to facilitate the extraction of water-soluble phytochemicals. Aqueous extraction was selected because polar phytochemicals such as phenolic compounds, flavonoids, and polysaccharides are known to act as reducing and stabilizing agents in plant-mediated nanoparticle synthesis. Moderate heating enhances the diffusion of these compounds from the plant matrix and improves extraction efficiency while minimizing the degradation of thermolabile constituents. Similar extraction conditions have been widely employed in previous studies on the green synthesis of metallic nanoparticles using plant extracts. After extraction, the mixture was filtered through Whatman No. 1 filter paper (Cytiva, Maidstone, UK), and the filtrate was collected for subsequent nanoparticle synthesis. To ensure reproducibility, all extraction and synthesis parameters were strictly standardized. Equal plant mass, solvent volume, extraction temperature, and time were used for all samples. The concentration of silver nitrate(Merck KGaA, Darmstadt, Germany) (1 mM), extract-to-precursor ratio (10:1, *v*/*v*), and reaction conditions were kept constant throughout the study. These controlled parameters ensured consistent nanoparticle formation across independent preparations.

### 4.2. Chemical Characterization of Plant Extracts

In the present work, qualitative functional group identification was performed using FTIR spectroscopy (Nicolet iS10, Thermo Scientific, Waltham, MA, USA) to confirm the presence of chemical moieties capable of reducing and stabilizing silver nanoparticles. Detailed quantitative phytochemical profiling (e.g., HPLC or LC–MS analysis) was beyond the scope of this study; however, previously published chromatographic investigations of *Artemisia absinthium* and *Ziziphus vulgaris* have extensively documented their phenolic, flavonoid, terpenoid, and saponin contents [17,18,19,20]. The chemical composition of the aqueous extracts of *Artemisia absinthium* and *Ziziphus vulgaris* was characterized using Fourier-transform infrared (FTIR) spectroscopy to identify the principal functional groups involved in silver ion reduction and nanoparticle stabilization. Detailed quantitative phytochemical profiling of both plants has been extensively reported in the literature using validated chromatographic techniques, including HPLC and LC–MS [17,18,19,20]. For *Artemisia absinthium*, multiple HPLC-based studies [17,18] report total phenolic contents typically ranging from approximately 60 to 120 mg gallic acid equivalents (GAE)/g dry extract, and total flavonoid contents in the range of 20–40 mg quercetin equivalents (QE)/g extract, depending on plant part, geographical origin, and extraction conditions. The dominant phenolic and flavonoid constituents consistently identified include chlorogenic acid, caffeic acid, quercetin, and luteolin, while sesquiterpene lactones such as artemisinin are also reported as characteristic secondary metabolites of the genus *Artemisia* [17,18]. Reported HPLC studies indicate that these individual compounds are generally present at the mg/g level, with substantial variability among different samples. Similarly, *Ziziphus vulgaris* extracts have been shown to contain high levels of phenolic and flavonoid compounds, with total phenolic contents commonly reported in the range of 70–140 mg GAE/g extract and total flavonoid contents of approximately 15–45 mg QE/g extract [19,20]. HPLC analyses consistently identify rutin, quercetin, kaempferol, and gallic acid as major phenolic constituents. In addition, triterpenoids such as oleanolic acid and ursolic acid, along with a high content of saponins, are recognized as hallmark components of *Ziziphus* species. The total saponins represent a substantial fraction of the extract, frequently accounting for several tens of milligrams per gram of dry extract, depending on extraction methodology [19,20].

In the present study, FTIR spectra of both plant extracts (Figures S5 and S6) exhibited broad absorption bands at 3339–3239 cm^−1^, corresponding to O–H stretching vibrations of phenolic and flavonoid hydroxyl groups. Bands at 1630–1611 cm^−1^ were attributed to C=O stretching vibrations of carbonyl-containing compounds, including phenolic acids, flavonoids, sesquiterpene lactones, and triterpenoids. Additional absorption bands in the 1076–1014 cm^−1^ region correspond to C–O stretching vibrations of polysaccharides, glycosidic linkages, and saponins. These functional groups are well established as the chemical moieties responsible for Ag^+^ reduction and nanoparticle stabilization. The FTIR results obtained in this study are therefore fully consistent with the quantitatively documented phytochemical profiles of *Artemisia absinthium* and *Ziziphus vulgaris* reported in the literature [17,18,19,20], confirming the chemical suitability of the extracts for green nanoparticle synthesis. Although experimental quantification of total phenolic and flavonoid contents was not performed in the present study, previously reported chromatographic analyses provide a reliable chemical framework for interpreting the present results. The reported ranges of phenolic and flavonoid contents in *Artemisia absinthium* and *Ziziphus vulgaris* extracts strongly support their role as effective reducing and stabilizing agents in green nanoparticle synthesis. It is important to note that the present work follows a comparative experimental design under strictly identical synthesis conditions, which minimizes variability related to extraction and reaction parameters. Therefore, the observed differences in nanoparticle characteristics and biological activities can be reasonably attributed to inherent differences in phytochemical composition between the two plant systems. One limitation of this study is the absence of direct photochemical characterization, including total phenolic content (TPC), total flavonoid content (TFC), or HPLC-based chromatographic profiling of the extracts. To address this gap, we relied on previously published chromatographic investigations of the two studied plants to interpret the relationship between the extract nanocompositions and the assessed pharmacological activities. Nevertheless, future studies will incorporate quantitative phytochemical analysis (e.g., total phenolic content, total flavonoid content, and chromatographic profiling such as HPLC or LC–MS) to establish a more direct correlation between extract composition and nanoparticle properties.

### 4.3. Synthesis of Silver Nanoparticles

An aqueous 1 mM silver nitrate (AgNO_3_) solution served as the silver precursor. Plant extract and AgNO_3_ solution were mixed at a 10:1 ratio (*v*/*v*) under ambient light and temperature. The reaction mixture was stirred gently until the color changed from pale yellow to dark brown, indicating the reduction of Ag^+^ ions to metallic silver nanoparticles, (Figure 1 and Figure 2). The colloidal suspensions were centrifuged at 10,000 rpm for 20 min, washed three times with distilled water, and dried under vacuum. Separate nanoparticle batches were produced from *Artemisia* leaves, *Artemisia* stems, *Ziziphus* leaves, and *Ziziphus* stems. Silver nanoparticles were synthesized by mixing the prepared plant extracts with an aqueous solution of silver nitrate (AgNO_3_, 1 mM). The extract-to-precursor ratio was maintained at 10:1 (*v*/*v*). The reaction mixture was kept under continuous stirring to allow efficient interaction between the plant-derived phytochemicals and Ag^+^ ions, leading to the reduction of silver ions and formation of AgNPs. All synthesis parameters, including precursor concentration, extract volume, reaction temperature, and reaction time, were maintained constantly to ensure reproducibility of nanoparticle formation. After completion of the reaction, the mixture was filtered to remove residual plant material, and the obtained nanoparticle suspension was used for subsequent characterization and biological activity assays.

### 4.4. Characterization of Nanoparticles

Formation of AgNPs was preliminarily monitored by visual observation and subsequently confirmed using UV–Visible spectroscopy (UV-2600, Shimadzu, Kyoto, Japan) in the range of 300–700 nm to detect the characteristic surface plasmon resonance (SPR) band of silver nanoparticles. Fourier-transform infrared (FTIR) spectra were recorded using an FTIR spectrometer (Nicolet iS10, Thermo Scientific, Waltham, MA, USA) in the range of 4000–400 cm^−1^ using the KBr pellet method to identify functional groups involved in nanoparticle reduction and stabilization. Transmission Electron Microscopy (TEM) analysis was performed using a JEOL JEM-2100 microscope (JEOL Ltd., Tokyo, Japan) operated at an accelerating voltage of 200 kV to determine nanoparticle morphology and size distribution. Scanning Electron Microscopy (SEM) images were obtained using a Quanta 250 FEG SEM (FEI Company, Hillsboro, OR, USA) to examine surface morphology and aggregation behavior.

The hydrodynamic diameter and colloidal stability of the synthesized AgNPs were further characterized by dynamic light scattering (DLS) and zeta potential measurements (by electrophoretic light scattering/laser Doppler velocimetry) using a Malvern ZetaSizer Nano ZS (Malvern Panalytical Ltd., Malvern, UK) instrument equipped with a He–Ne laser (λ = 633 nm, 4 mW) and 173° backscatter detection (NIBS technology). Measurements were performed at 25.0 ± 0.1 °C (Peltier-controlled) after a 120 s thermal equilibration period, with automatic optimization of run parameters (typically 10–15 sub-runs per measurement). The dispersant was deionized water (refractive index = 1.330, viscosity = 0.8872 cP at 25 °C), and the optical properties of silver were set as refractive index = 0.135 and absorption coefficient = 3.413. The sample preparation protocol for DLS and zeta potential measurements is detailed in the Supplementary Material Section 3.

Crystallographic phase identification and structural properties of the synthesized AgNPs were characterized by X-ray diffraction (XRD) using a Bruker D8 Advance diffractometer (Bruker AXS GmbH, Karlsruhe, Germany) equipped with Cu Kα radiation (λ = 1.5406 Å) operated at 40 kV and 30 mA. Diffraction patterns were recorded over a 2θ range of 10–80° with a step size of 0.02° and a scan speed of 2° min^−1^, using a scintillation counter (or position-sensitive detector) and automatic divergence slit optics to maintain a constant irradiated area. Sample preparation protocol for XRD measurements is detailed in Supplementary Material Section 4.

The energy-dispersive X-ray spectroscopy (EDX) is an X-ray microanalysis technique used to determine the elemental composition of a sample; in most nanomaterials studies it is implemented as an accessory detector on a scanning electron microscope (SEM). The surface and structural morphology of the prepared samples were characterized using high-resolution Scanning Electron Microscopy (HR-SEM) on a FEI Quanta FEG 250 instrument (FEI, Hillsboro, OR, USA).

While the initially applied characterization techniques (UV–Vis, TEM, SEM, and FTIR) provided robust confirmation of nanoparticle formation, morphology, and surface functionalization, additional physicochemical analyses—including dynamic light scattering (DLS), zeta potential measurements, X-ray diffraction (XRD), and energy-dispersive X-ray spectroscopy (EDX)—were subsequently performed to further enhance nanoparticle characterization. Specifically, DLS provided the hydrodynamic size distribution in colloidal systems, zeta potential quantified surface charge and colloidal stability, XRD confirmed crystallinity and phase structure, and EDX verified elemental composition. These complementary analyses collectively offer a more comprehensive understanding of the synthesized nanoparticles, and strengthening the overall characterization protocol.

### 4.5. Antibacterial Activity

Antibacterial activity was assessed against *Escherichia coli* (Gram-negative) and *Staphylococcus aureus* (Gram-positive) employing the agar well diffusion assay. *Staphylococcus aureus* ATCC 6538 and *Escherichia coli* ATCC 8739 were obtained from the Cairo Microbiological Resources Centre (Cairo MIRCEN), Egyptian Microbial Culture Collection (EMCC), Faculty of Agriculture, Ain Shams University, Cairo, Egypt. Nanoparticle suspensions (50 µL, 100 µg mL^−1^) were loaded into wells of 6 mm diameter. The plates were then incubated at 37 °C for 24 h, after which the zones of inhibition were recorded in millimeters. Trials were repeated in triplicate. Control experiments were systematically performed using plant extracts alone and silver nitrate solution (1 mM) to distinguish the intrinsic antibacterial effects of the extracts and precursors from those of the biosynthesized AgNPs. Summary of MIC and MBC procedures for the biosynthesized nanoparticles is displayed in Supplementary Section S1.

### 4.6. Anti-Inflammatory Activity

The membrane-stabilizing activity of the nanoparticles was evaluated using a hypotonicity-induced erythrocyte hemolysis model. Human red blood cells were suspended in hypotonic saline containing various concentrations of nanoparticles (100–1000 µg mL^−1^) and incubated at 37 °C for 1 h. Hemolysis was quantified spectrophotometrically at 540 nm. Percentage inhibition of hemolysis was considered relative to control samples. Isotonic saline served as the negative control (no hemolysis), while hypotonic saline without nanoparticles served as the positive hemolysis control.

### 4.7. Cytotoxicity Assay (MTT Protocol)

MCF-7 human breast cancer cells were seeded into 96-well plates at a density of 1 × 10^5^ cells mL^−1^. After initial 24 h incubation, the cells were exposed to serial dilutions of nanoparticle suspensions for 24 h. Cell viability was evaluated using the MTT assay, in which 20 µL of MTT solution (5 mg mL^−1^) was added, followed by a 4 h incubation. Formazan crystals were then solubilized in dimethyl sulfoxide (DMSO), and absorbance was measured at 560 nm (background 620 nm). IC50 values were determined from dose–response curves. Untreated biological cells maintained under the same culture environments were used as the negative control and considered 100% viable for the calculation of relative cell viability. To exclude the potential interference of nanoparticles with the colorimetric MTT assay, control wells containing the tested nanoparticle formulations without cells were included to evaluate any background absorbance caused by light scattering or direct interaction with the MTT reagent. The absorbance values obtained from these nanoparticle-only controls were subtracted from the corresponding experimental measurements. Cell viability was expressed as a percentage relative to untreated control cells. Dose–response curve was generated using GraphPad Prism software version 11.1.0 (GraphPad Software, San Diego, CA, USA), and the IC_50_ values were assessed via nonlinear regression analysis.

### 4.8. Source of Cell Lines and Biological Materials

The MCF-7 human breast adenocarcinoma (ATCC^®^ HTB-22™) and HepG2 human hepatocellular carcinoma (ATCC^®^ HB-8065™) cell lines were purchased from the American Type Culture Collection (ATCC, Manassas, VA, USA) and authenticated by the supplier via short tandem repeat (STR) profiling. Cells were grown in Dulbecco’s Modified Eagle’s Medium (DMEM) containing 10% fetal bovine serum (FBS) and 1% penicillin–streptomycin and were kept at 37 °C in a humidified 5% CO_2_ incubator. Cell passages below 20 were used for all experiments. Human red blood cells (HRBCs) used in the hypotonicity-induced hemolysis assays were obtained from residual blood samples collected during routine laboratory procedures in the Department of Zoology, Faculty of Science, University of Derna, Libya. The samples were obtained from healthy volunteer donors who had provided informed consent for the use of their blood for laboratory and research purposes. All samples were anonymized prior to use, and no personal or identifiable information was associated with the materials used in this study. According to the institutional laboratory guidelines of the University of Derna, the use of anonymized residual human blood samples for in vitro experimental assays does not require formal Institutional Review Board (IRB) approval.

All experiments were performed using three independent biological replicates, and each measurement was conducted in triplicates as technical replicates. The results are expressed as mean ± standard error (SE) unless otherwise stated. Statistical analysis was performed using one-way analysis of variance (ANOVA) to evaluate differences between treated and control groups. When significant differences were noted, post hoc comparisons were performed using Tukey’s statistical test. Significance was defined as a *p*-value of less than 0.05.

### 4.9. Procedures for Scratch Wound Healing Assay

WI-38 cells were seeded in 6-well plates and cultured until confluence was achieved. A straight scratch was then introduced into each monolayer using a yellow pipette tip held at approximately 30° to ensure a consistent and limited initial wound width. After 48 h of incubation, the wounded areas were imaged using a 10× objective, and wound lengths were measured.

## 5. Conclusions

This study demonstrates a rapid, eco-friendly synthesis of silver nanoparticles (AgNPs) using aqueous extracts of *Artemisia absinthium* and *Ziziphus vulgaris* which are widely available plants that are often discarded or burned as agricultural waste; therefore, transforming this abundant biomass into high-value nanomaterials with therapeutic potential not only reduces environmental burden but also supports the sustainable, circular use of natural resources. Under ambient conditions, both plants produced stable, predominantly spherical nanoparticles (15–22 nm) exhibiting strong surface plasmon resonance at 420–450 nm; Fourier-transform infrared spectra confirmed the involvement of hydroxyl and carbonyl groups in metal ion reduction and nanoparticle capping, while TEM/SEM analyses revealed uniform morphology with excellent colloidal stability for at least 90 days. All samples exhibited a face-centered cubic (FCC) structure of metallic silver in XRD analysis. The EDX spectra of all samples confirmed silver (Ag) as the major constituent. DLS revealed that all samples displayed a monomodal size distribution. Zeta potential measurements showed consistently negative values, indicating a robust electrical double layer that ensures good colloidal stability.

The biosynthesized AgNPs demonstrated potent antibacterial activity against *Staphylococcus aureus* and *Escherichia coli*, concentration-dependent anti-inflammatory effects in hypotonicity induced hemolysis assays, and significant cytotoxicity toward MCF-7 breast cancer cells with IC_50_ values as low as 19 µg mL^−1^. Furthermore, the positive scratch wound repair assay results suggest their potential as promising agents for topical wound healing. It should be noted that all the investigated therapeutic and biological activities are preliminary as they are based on in vitro assays that require further in vivo confirmation. A distinctive feature of this study is the direct side-by-side comparison of two medicinal plants with contrasting phytochemical profiles under identical synthesis conditions, providing clear evidence of how plant metabolites influence nanoparticle formation and biological performance. These findings establish a rational framework for selecting botanical sources to design green nanomaterials with tailored biomedical properties while contributing to a cleaner and more sustainable environment.

## Figures and Tables

**Table 1:** Z-average (hydrodynamic diameter) of AgNPs prepared using the studied plant extracts.

Samples	The Z-Average (Hydrodynamic Diameter)
*Artemisia* leaves	84.1 nm
*Artemisia* stems	87.6 nm
*Ziziphus* leaves	88.6 nm
*Ziziphus* stems	88.4 nm

**Table 2:** Mean zeta potential of AgNPs prepared using the studied plant extracts.

Plant Samples	Mean Zeta Potential(mV)	Stability Status
*Artemisia* leaves	−33.19	Good Stability
*Artemisia* stems	−33.80	Good Stability
*Ziziphus* leaves	−34.16	Good Stability
*Ziziphus* stems	−34.40	Good Stability

**Table 3:** Summary of XRD parameters for AgNPs prepared using the studied plant extracts.

Plants	Crystal System	Crystal Size (nm)	Crystallinity (%)	Main Peak
*Artemisia* leaves	Cubic (FCC)	22	82%	(111)
*Artemisia* stems	Cubic (FCC)	20	79%	(111)
*Ziziphus* leaves	Cubic (FCC)	19	77%	(111)
*Ziziphus* stems	Cubic (FCC)	18	73%	(110)

**Table 4:** Elemental composition (EDX) of AgNPs prepared using the four plant extracts.

Plants	Main Element	Weight % (Range)	Atom% (Range)
*Artemisia* leaves	Ag	76.1–69.7	29.7–24.3
*Artemisia* stems	Ag	82.6	38.6
*Ziziphus* leaves	Ag	86.7–76.4	46.8–33.5
*Ziziphus* stems	Ag	89.2–78.2	52.9–37.0

Weight %: relative mass percentage of each element in the analyzed sample. Atom %: atomic percentage of each element relative to the total number of atoms detected. The major Ag peaks appear at approximately 3 keV at Figure 13, corresponding to the characteristic Ag Lα X-ray emission.

**Table 5:** The effect of *Ziziphus* Leaf extract before adding nanoparticles.

Concentrations (mg/mL)	Inhibition Zone (mm) *Escherichia coli*	Inhibition Zone (mm) *Staphylococcus aureus*
25	2	4
50	5	5
75	3	3
100	4	5

**Table 6:** The effect of *Artemisia* plant leaf’s extract before adding nanoparticles.

Concentrations (mg/mL)	Inhibition Zone (mm) *Escherichia coli*	Inhibition Zone (mm) *Staphylococcus aureus*
25	7	6
50	9	7
75	10	6
100	11	10

**Table 7:** Summary of minimum inhibitory concentration (MIC) and minimum bactericidal concentration (MBC) values biosynthesized AgNPs from four plant extracts.

**Microorganism**	**MIC (** **µg mL^−1^** **)**			
	sample 1 *Artemisia* leaves	sample 2 *Artemisia* stems	sample 3 *Ziziphus* leaves	sample 4 *Ziziphus* stems
*St. aureus* (ATCC 6538)	62.2 ± 0.0	125 ± 0.0	31.25 ± 0.0	15.62 ± 0.0
*E. coli* (ATCC 8739)	31.25 ± 0.0	125 ± 0.0	31.25 ± 0.0	15.62 ± 0.0
**Microorganism**	**MBC (µg mL** ^**−1**^ **)**			
	sample 1 *Artemisia* leaves	sample 2 *Artemisia* stems	sample 3 *Ziziphus* leaves	sample 4 *Ziziphus* stems
*St. aureus* (ATCC 6538)	125 ± 0.0	250 ± 0.0	31.25 ± 0.0	15.62 ± 0.0
*E. coli* (ATCC 8739)	125 ± 0.0	500 ± 0.0	62.5 ± 0.0	31.25 ± 0.0

**Table 8:** Effect of *Artemisia* Leaf’s on HRBC hemolytic and membrane stabilization.

*Artemisia* Leaf’s Conc. µg mL^−1^	OD R1	OD R2	OD R3	Hypotonic Ab. Mean	Sample with Isotonic Solution Ab.	Hemolysis Inhibition %	SD	SE
**Control**	0.984	0.987	0.982	0.984	0.001	0.0	0.003	0.001
1000	0.056	0.061	0.048	0.055	0.032	97.6	0.007	0.002
500	0.093	0.099	0.096	0.096	0.026	92.7	0.003	0.001
250	0.114	0.117	0.12	0.117	0.022	90.1	0.003	0.001
125	0.219	0.221	0.225	0.222	0.017	78.8	0.003	0.001
62.5	0.307	0.309	0.313	0.310	0.012	69.4	0.003	0.001
31.25	0.4	0.403	0.408	0.404	0.01	59.6	0.004	0.001
15.62	0.496	0.499	0.498	0.498	0.004	49.6	0.002	0.000
7.8	0.596	0.604	0.607	0.602	0.003	38.9	0.006	0.002
3.9	0.717	0.723	0.729	0.723	0.001	26.6	0.006	0.002

**Table 9:** Effect of Sample *Ziziphus* Leaf’s on HRBC hemolytic and membrane stabilization.

*Ziziphus spina*-leaf’s Conc. µg mL^−1^	OD R1	OD R2	OD R3	Hypotonic Ab. Mean	Sample with Isotonic Solution Ab.	Hemolysis Inhibition %	SD	SE
Control	0.984	0.987	0.982	0.984	0.001	0.0	0.003	0.001
1000	0.127	0.129	0.13	0.129	0.023	89.0	0.002	0.000
500	0.187	0.194	0.199	0.193	0.019	81.9	0.006	0.002
250	0.254	0.255	0.248	0.252	0.016	75.6	0.004	0.001
125	0.336	0.339	0.341	0.338	0.013	66.6	0.002	0.001
62.5	0.422	0.428	0.433	0.428	0.008	57.0	0.006	0.002
31.25	0.516	0.512	0.519	0.516	0.005	47.8	0.004	0.001
15.62	0.593	0.599	0.597	0.596	0.004	39.6	0.003	0.001
7.8	0.687	0.692	0.693	0.691	0.002	29.9	0.003	0.001
3.9	0.794	0.799	0.801	0.798	0.001	18.9	0.004	0.001

**Table 10:** Cytotoxic activity of the synthesized nanoparticles against MCF-7 human breast cancer cells determined by the MTT assay. Cell viability values represent mean ± SD of three independent experiments. IC_50_ values were calculated from dose–response curves using nonlinear regression analysis.

Samples	µg/mL	O.D			Mean O.D	±SD	Cell Viability (%)	Cytotoxicity (%)	IC50 ± SD
Control (untreated cells) Mcf7	—	0.753	0.749	0.748	0.750	0.00152	100	0.00	µg
1	250	0.020	0.019	0.022	0.020	0.00088	2.71	97.28	13.77 ± 0.11
	125	0.021	0.026	0.023	0.023	0.00145	3.11	96.88	
	62.5	0.022	0.025	0.022	0.023	0.00101	3.06	96.93	
	31.25	0.026	0.024	0.026	0.025	0.00066	3.37	96.62	
	15.62	0.197	0.201	0.215	0.204	0.00545	27.24	72.75	
	7.81	0.576	0.562	0.555	0.564	0.00617	75.24	24.75	
2	250	0.022	0.023	0.020	0.021	0.00088	2.88	97.11	15.95 ± 0.14
	125	0.024	0.025	0.025	0.024	0.00033	3.28	96.71	
	62.5	0.042	0.046	0.055	0.047	0.00384	6.35	93.64	
	31.25	0.112	0.143	0.133	0.129	0.00913	17.24	82.75	
	15.62	0.314	0.288	0.296	0.299	0.00768	39.91	60.08	
	7.81	0.576	0.562	0.555	0.564	0.00617	75.24	24.75	
3	250	0.020	0.018	0.021	0.019	0.00066	2.57	97.42	37.85 ± 0.22
	125	0.022	0.023	0.024	0.023	0.00057	3.06	96.93	
	62.50	0.055	0.064	0.062	0.060	0.00272	8.04	91.95	
	31.25	0.389	0.377	0.392	0.386	0.00458	51.46	48.53	
	15.62	0.734	0.744	0.740	0.739	0.00290	98.57	1.42	
	7.81	0.747	0.744	0.753	0.748	0.00264	99.73	0.26	
4	250	0.033	0.025	0.028	0.028	0.00233	3.82	96.17	19.54 ± 0.15
	125	0.042	0.046	0.038	0.042	0.00230	5.61	94.40	
	62.50	0.057	0.053	0.062	0.057	0.00260	7.64	92.35	
	31.25	0.098	0.113	0.121	0.110	0.00674	14.75	85.24	
	15.62	0.389	0.377	0.381	0.382	0.00360	50.93	49.06	
	7.81	0.742	0.733	0.746	0.740	0.00384	98.71	1.28	

**Table 11:** Summary of half-maximal inhibitory concentration (IC_50_) values, calculated mathematically, for the biosynthesized nanoparticles in DMSO against the non-cancerous WI-38 cell line.

Cells	Samples	IC_50_ (µg/mL)
**Wi38**	*Artemisia* leaves	>>250
	*Artemisia* stems	208.71
	*Ziziphus* leaves	233.68
	*Ziziphus* stems	>>250

**Table 12:** Calculated wound-closure percentages and wound areas (µm^2^) for the four nanoparticle samples and the untreated WI-38 control in the scratch wound healing assay.

Item	at 0h		at 48 h		RM µm	Wound Closure % µm^2^	Area Difference
	Width	Area	Width	Area			
Control wi38	1274.025	1370889.121	270	290528.1			
	1276.025	1375557.502	158	170324.3			
	1316.002	1413388.78	346.006	371611.1			
	1348.024	1447780.472	388.021	416735.3			
	1306.006	1402653.056	346.023	371629.4			
	1304.014	1405814.461	244	263048.3			
mean	1304.016	1402680.565	292.0083	313979.4	21.08349	77.61576	1088701
1	1468.001	1579613.116	184.011	198001.4			
	1460.067	1573955.146	82.098	88501.81			
	1478	1587374.956	202.01	216959.1			
	1430.022	1535846.488	166	178284.3			
	1470.022	1578806.568	60	64440.12			
	1536.22	1656148.087	208	224237.9			
mean	1473.722	1585290.727	150.3532	161737.4	27.57018	89.79762	1423553
2	1588	1708735.64	150.12	161533.6			
	1502.001	1619160.082	264.189	284796.3			
	1556.032	1671181.48	236.136	253610.5			
	1584.102	1701328.716	404.02	433918.3			
	1580.032	1696957.528	264.03	283568.7			
	1600.08	1724993.445	262.069	282527.9			
mean	1568.375	1687059.482	263.4273	283325.9	27.1864	83.20593	1403734
3	1692.011	1820654.596	132	142036			
	1670.005	1800268.73	144	155232.3			
	1668.145	1791591.066	24.083	25865.19			
	1672.097	1795835.522	92.087	98901.62			
	1640.03	1761395.5	66.121	71014.09			
	1714.005	1847812.228	206.01	222092.6			
mean	1676.049	1802926.274	110.7168	119190.3	32.61108	93.38906	1683736
4	1328.074	1429047.466	292	314200.8			
	1326.054	1429488.864	276.065	297598.6			
	1296.006	1391913.036	142	152508.3			
	1436.139	1542416.158	148.216	159184.3			
	1384.006	1486425.212	120.067	128952.2			
	1430.006	1541642.278	156.461	168675.4			
mean	1366.714	1470155.503	189.1348	203519.9	24.5329	86.15657	1266636

## Data Availability

The original contributions presented in the study are included in the article/Supplementary Materials, further inquiries can be directed to the corresponding authors.

## Supplementary Materials

The following supporting information can be downloaded at: https://www.mdpi.com/article/10.3390/ph19101630/s1, Figures S1 and S2: SEM analysis of *Artemisia* and *Ziziphus* leaf nanoparticles; Figures S3 and S4: TEM analysis of *Artemisia* and *Ziziphus* leaf nanoparticles; Figure S5: IR spectrum of *Artemisia* leaf nanoparticles; Figures S6–S8: DLS analysis of *Artemisia* and *Ziziphus* nanoparticles; Figures S9–S11: Zeta potential analysis of Ag NPs synthesized using *Artemisia* and *Ziziphus* extracts; Figures S12–S14: XRD analysis of Ag NPs synthesized using *Artemisia* and *Ziziphus* extracts; Figures S15–S17: EDX spectra of Ag NPs prepared using *Artemisia* and *Ziziphus* extracts; Figures S18–S21: Effects of *Ziziphus* and *Artemisia* extracts on Staphylococcus and *E. coli*; Figures S22–S25: Antibacterial effects of *Ziziphus* and *Artemisia* nanoparticles; Figures S26–S29: Anti-inflammatory effects of *Artemisia* and *Ziziphus* extracts; Figures S30–S34: Control and effects of Samples 1–4 on MCF-7 cells; Figures S35–S39: Control and effects of Samples 1–4 on HepG2 cells; Figure S40: Control of A549 cells; Figures S41–S44: Effects of Samples 1–4 on A549 cells; Figures S45–S49: Control and effects of Samples 1–4 on WI-38 cells; Figures S50–S52: Scratch wound healing assay of WI-38 fibroblasts treated with Samples 2–4; Tables S1–S6: Antibacterial effects of plant extracts and nanoparticles; Table S7: MIC and MBC data for the four nanoparticle samples; Tables S8 and S9: Hemolytic and membrane-stabilization effects of *Artemisia* and *Ziziphus* stems; Table S10: Cytotoxic activity of the synthesized nanoparticles against HepG2 cells; Table S11: Effects of the studied samples on A549 cells, including viability, toxicity, and IC_50_ values; Table S12: Viability, toxicity, and IC_50_ data for WI-38 cells; Table S13: Scratch wound healing assay data for WI-38 cells. Supplementary Section S1: Summary of MIC and MBC Determination Procedures for Biosynthesized Nanoparticles. Supplementary Section S2: Calculations for Scratch Wound Healing Assay. Supplementary Section S3: The sample preparation protocol for DLS and zeta potential measurements. Supplementary Section S4: The sample preparation protocol for XRD measurements.
