# Supplementary material for: Green Synthesis of Silver Nanoparticles Using Artemisia absinthium and Ziziphus vulgaris Extracts: Comparative Evaluation of Antibacterial, Anti-Inflammatory, Wound-Healing and Anticancer Activities
Source: Pharmaceuticals (Basel). 2026 Oct 14;19(10):1630. doi: 10.3390/ph19101630 (PMC13708049; doi:10.3390/ph19101630)
Supplement: Supplementary file 1 [file pharmaceuticals-19-01630-s001.zip › pharmaceuticals-4599612-supplementary.pdf]

Green Synthesis of Silver Nanoparticles Using *Artemisia absinthium* and *Ziziphus vulgaris* Extracts: Comparative Evaluation of Antibacterial, Anti-Inflammatory, Wound-Healing and Anticancer Activities

Mohammed Gamal [1,*], Asma B. Omer [2], Asmaa Saleh [3], Roula Bayram [4,5], Ibrahim A. Naguib [6], Mohamed Ahmed Elsayed [7,*], Safa Mousa [8] and Hamad M. Adress Hasan [9]

1 Pharmaceutical Analytical Chemistry Department, Faculty of Pharmacy, Beni-Suef University,
Alshaheed Shehata Ahmed Hegazy St., Beni-Suef 62574, Egypt
2 Department of Health Science, College of Health and Rehabilitation Sciences, Princess Nourah bint Abdulrahman University, P.O. Box 84428, Riyadh 11671, Saudi Arabia; abalfaki@pnu.edu.sa
3 Department of Pharmaceutical Sciences, College of Pharmacy, Princess Nourah bint Abdulrahman University, P.O. Box 84428, Riyadh 11671, Saudi Arabia; asali@pnu.edu.sa
4 Pharmaceutical Science, Pharmacy Program, Batterjee Medical College, Jeddah 21442, Saudi Arabia; lab3.jed@bmc.edu.sa
5 Department of Quality Control and Pharmaceutical Chemistry, Faculty of Pharmacy, University of Aleppo, Aleppo P.O. Box 12212, Syria
6 Department of Pharmaceutical Chemistry, College of Pharmacy, Taif University, Taif 21944, Saudi Arabia
7 Pharmaceutical Analytical Chemistry Department, Faculty of Pharmacy, Fayoum University,
Fayoum 63514, Egypt
8 Chemistry Department, Faculty of Science, AlQuba Campus, Derna University, Derna P.O. Box 125, Libya
9 Chemistry Department, Faculty of Science, Omar Al-Mukhtar University, Bayda P.O. Box 991, Libya
* Correspondence: mgamalm3000@yahoo.com (M.G.); mae06@fayoum.edu.eg (M.A.E.)

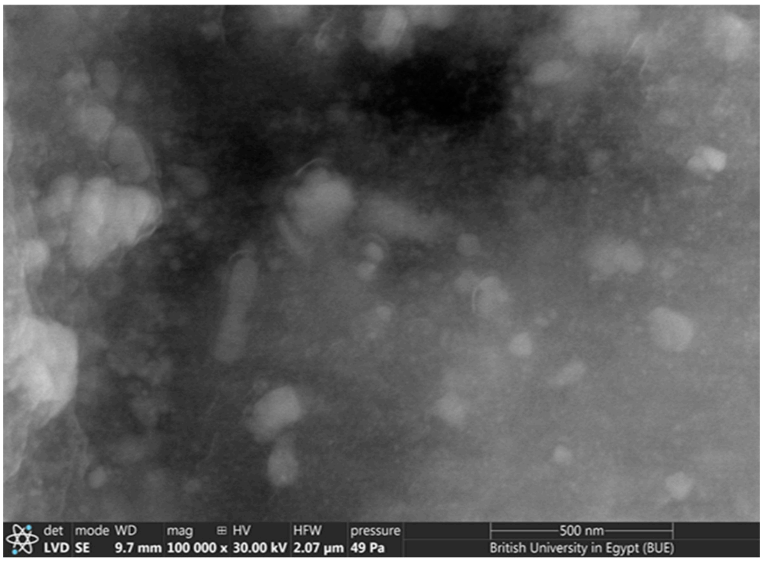

Figure S1. Scanning Electron Microscopy (SEM) analysis of *Artemisia* leaf nanoparticles.

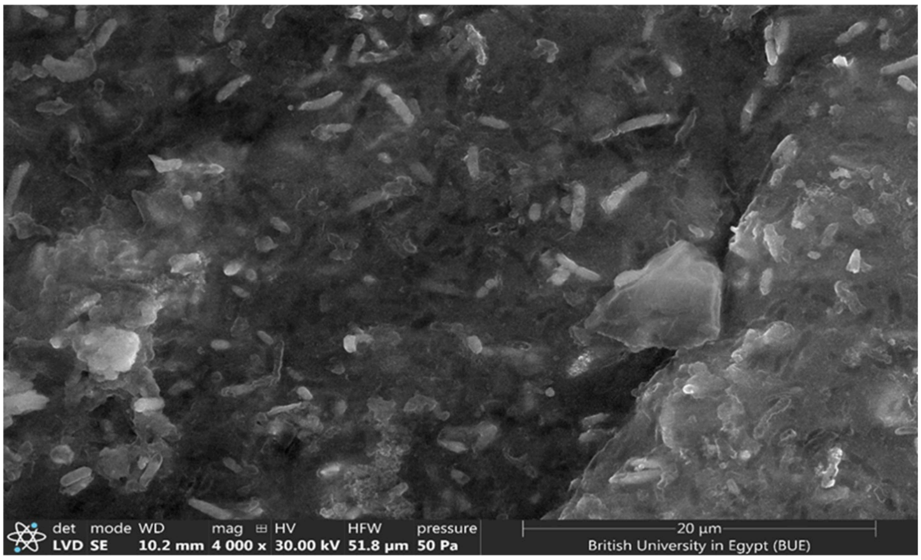

Figure S2. Scanning Electron Microscopy (SEM) analysis of Ziziphus leaf's nanoparticles.

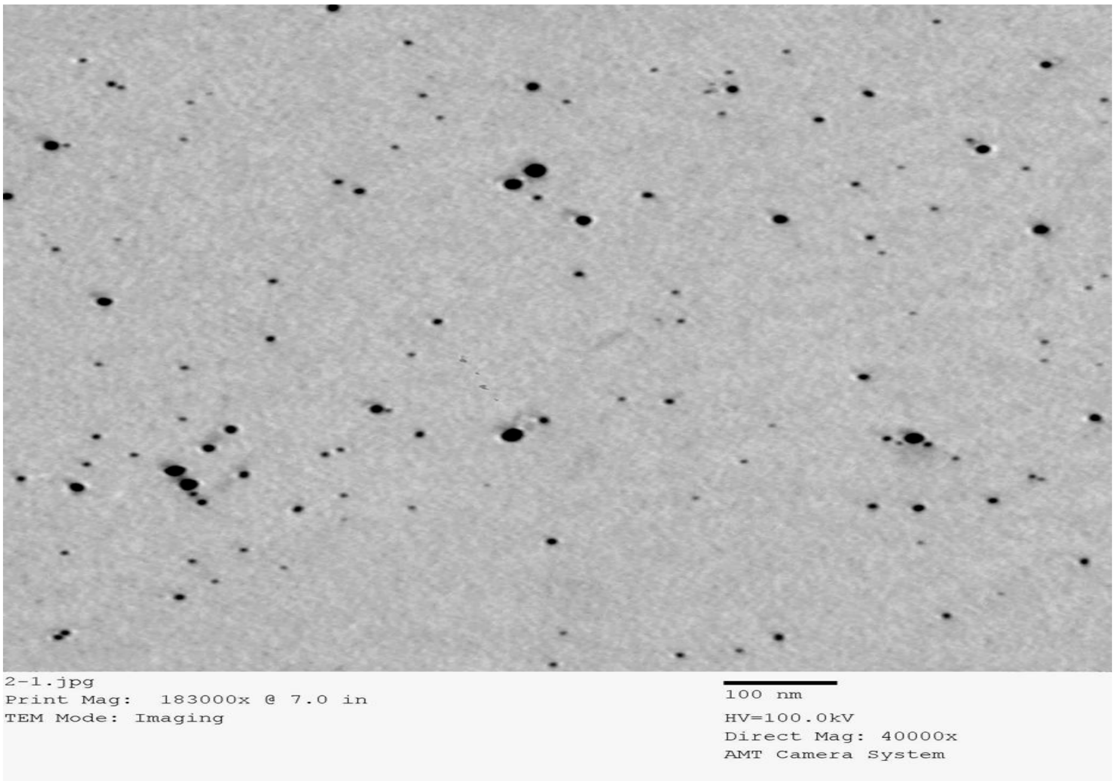

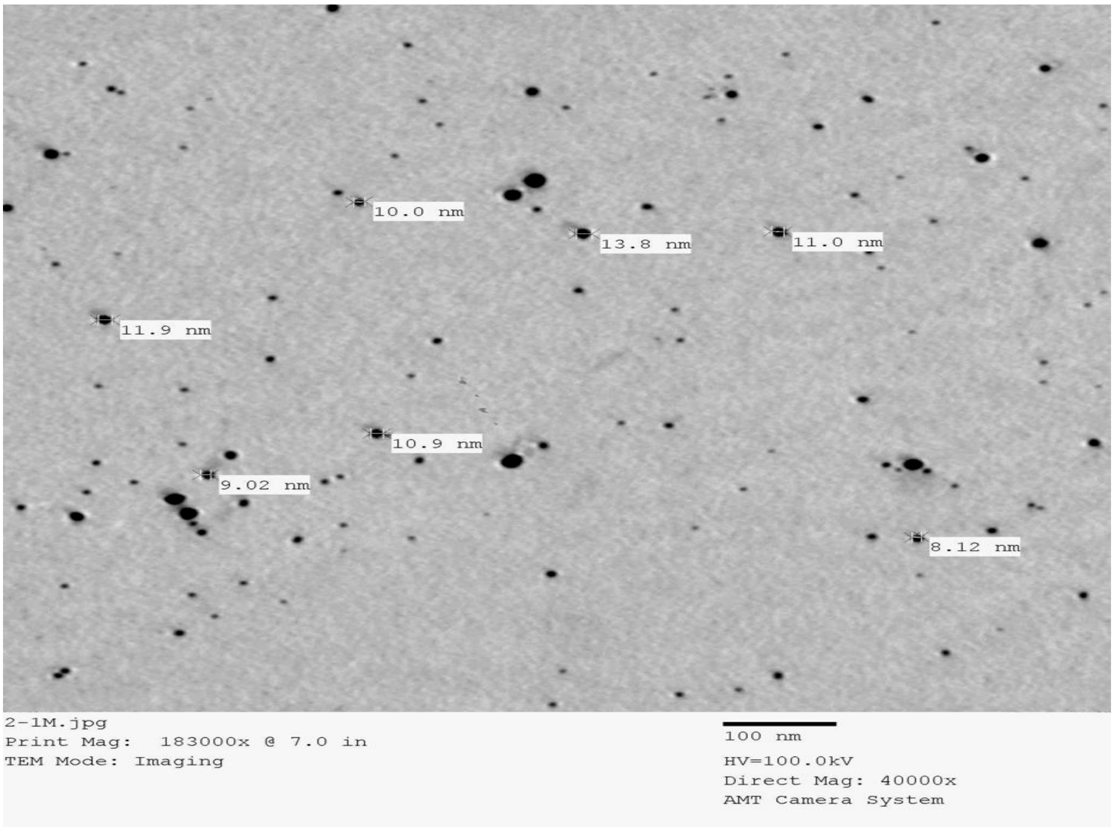

Figure S3. Transmission Electron Microscopy (TEM) analysis of Artemisia leaf's nanoparticles.

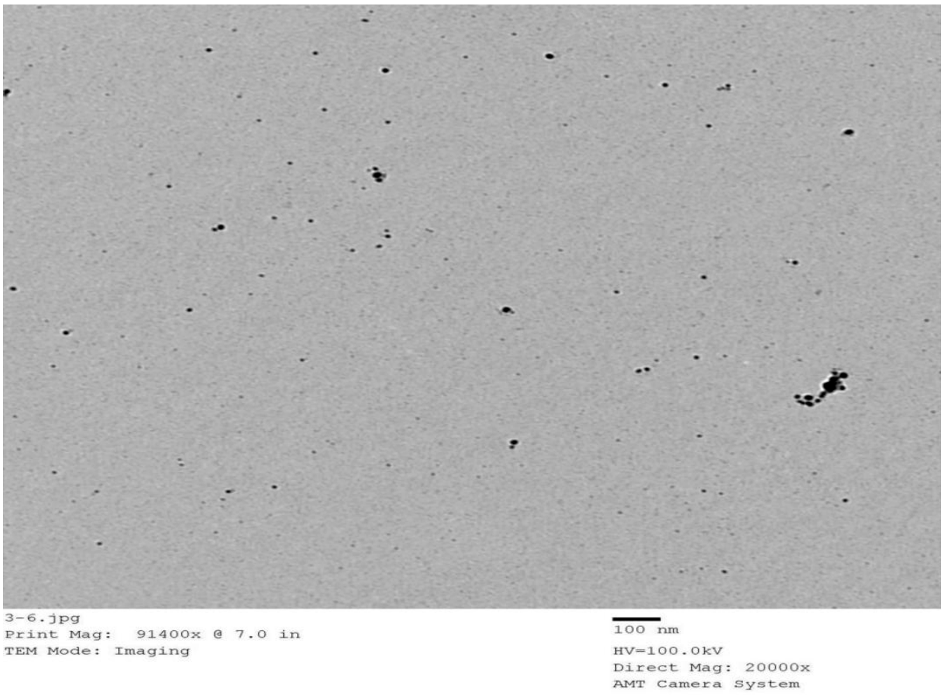

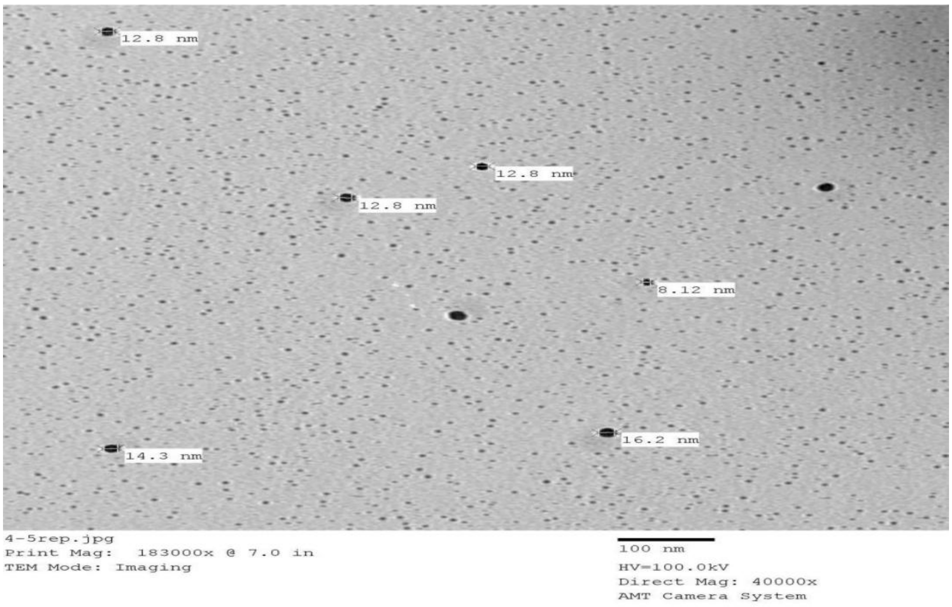

Figure S4. Transmission Electron Microscopy (TEM) analysis of Ziziphus leaf's nanoparticles.

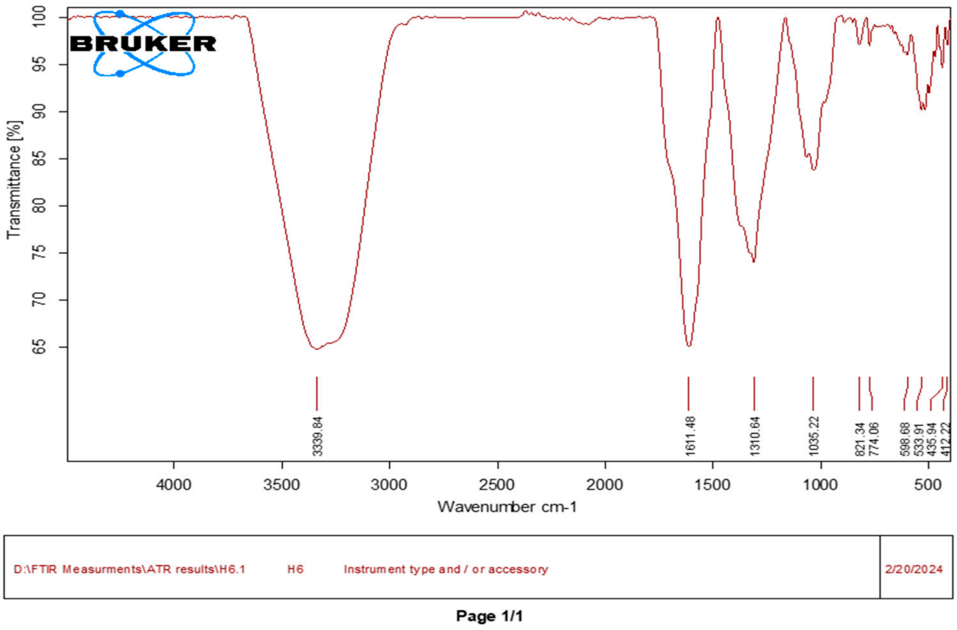

Figure S5. I.R Spectra of *Artemisia* leaf nanoparticles.

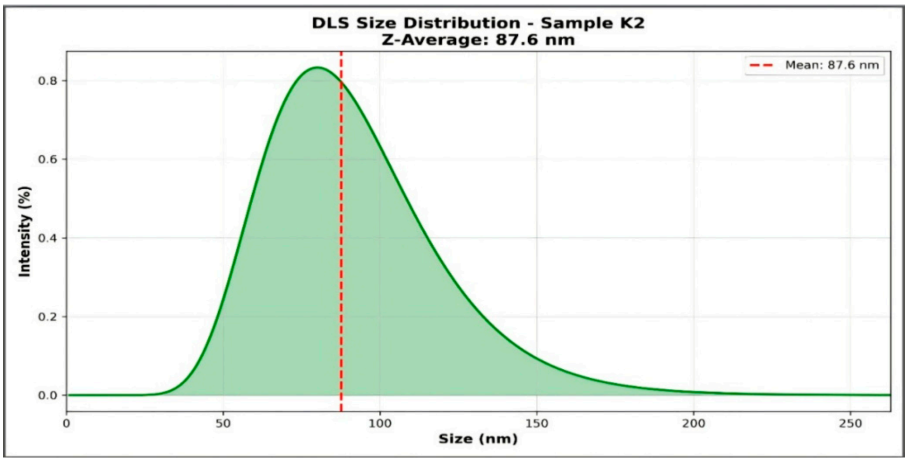

Figure S6. DLS of *Artemisia* stem nanoparticles.

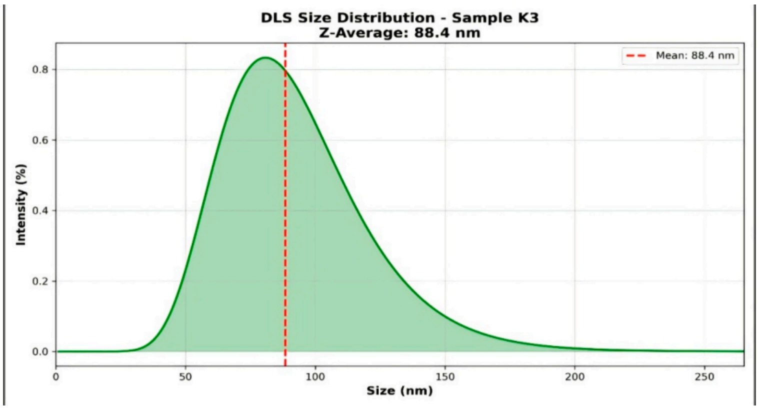

Figure S7. DLS of *Zizphus* leaf nanoparticles.

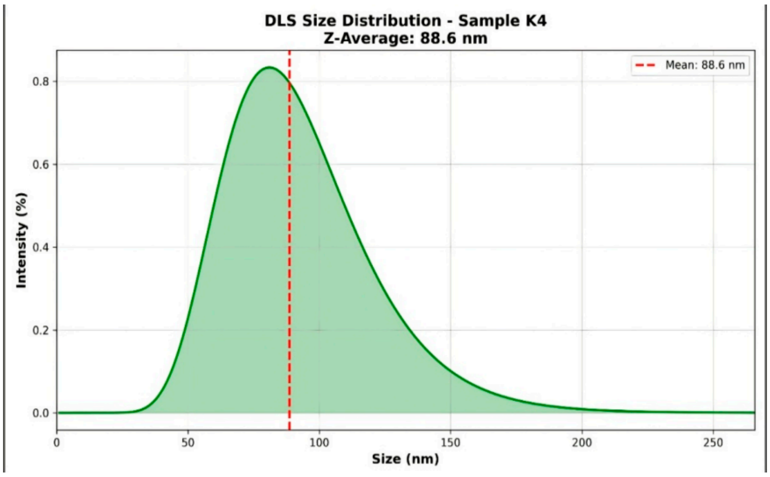

Figure S8. DLS of *Zizphus* stem nanoparticles.

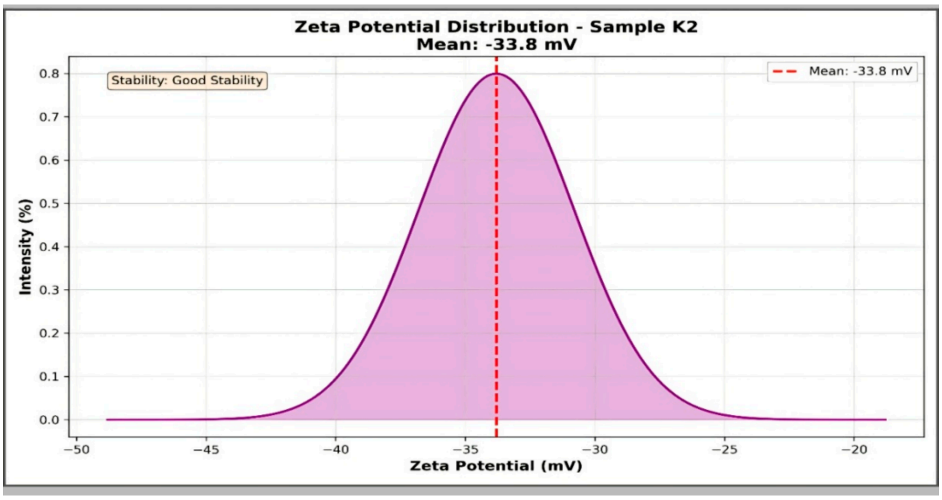

Figure S9. Zeta potential analysis of Ag NPs synthesized using *Artemisia* stem extract.

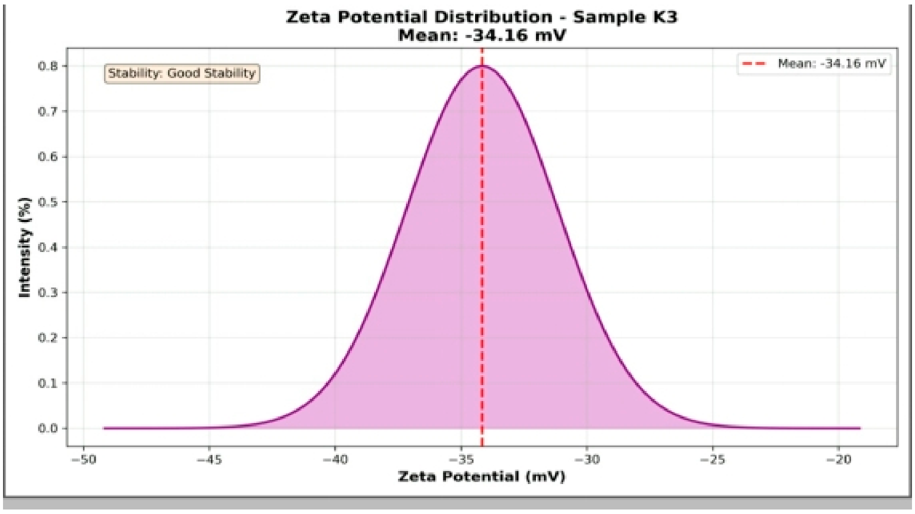

Figure S10. Zeta potential analysis of Ag NPs synthesized using *Ziziphus* leaf extract.

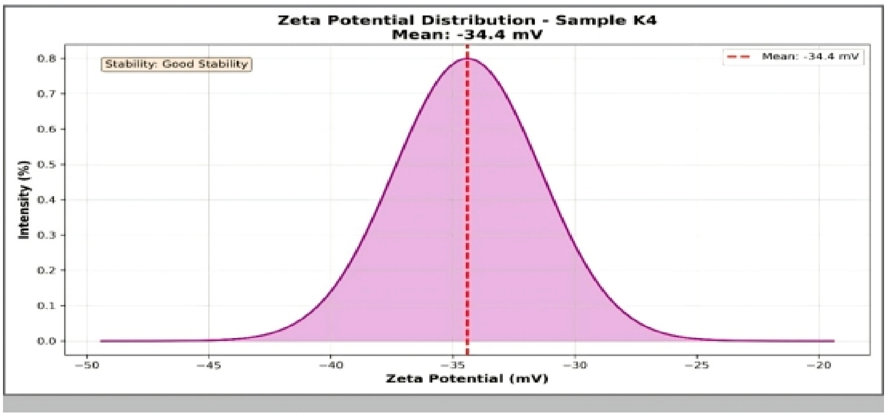

Figure S11. Zeta potential analysis of Ag NPs synthesized using *Ziziphus* stem extract.

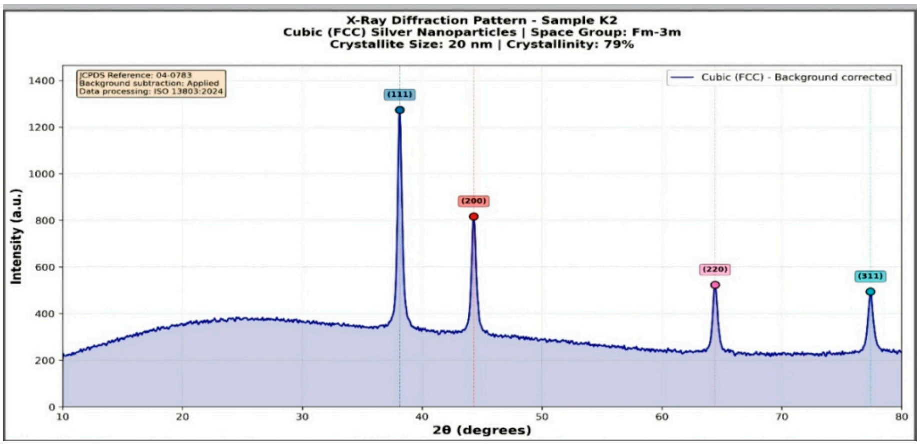

Figure S12. XRD of analysis of Ag NPs synthesized using *Artemisia* stem extract.

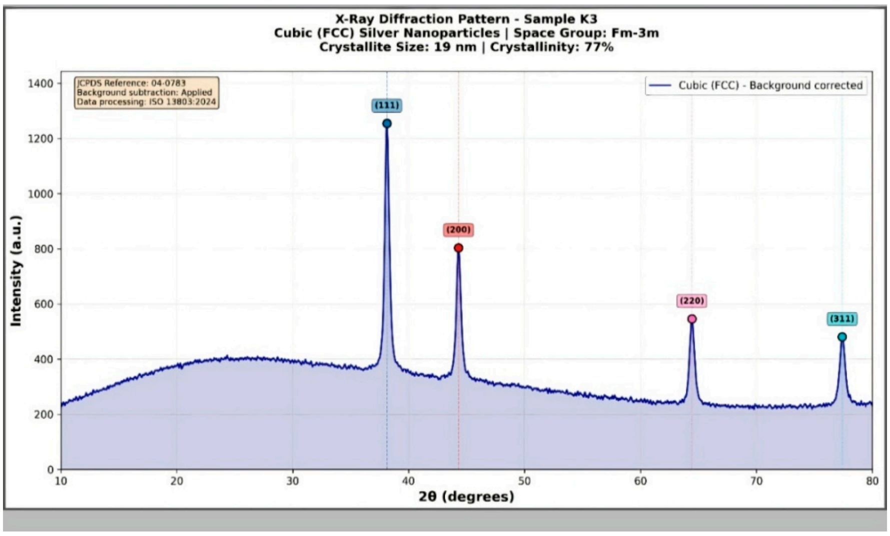

Figure S13. XRD of analysis of Ag NPs synthesized using *Ziziphus* Leaf extract.

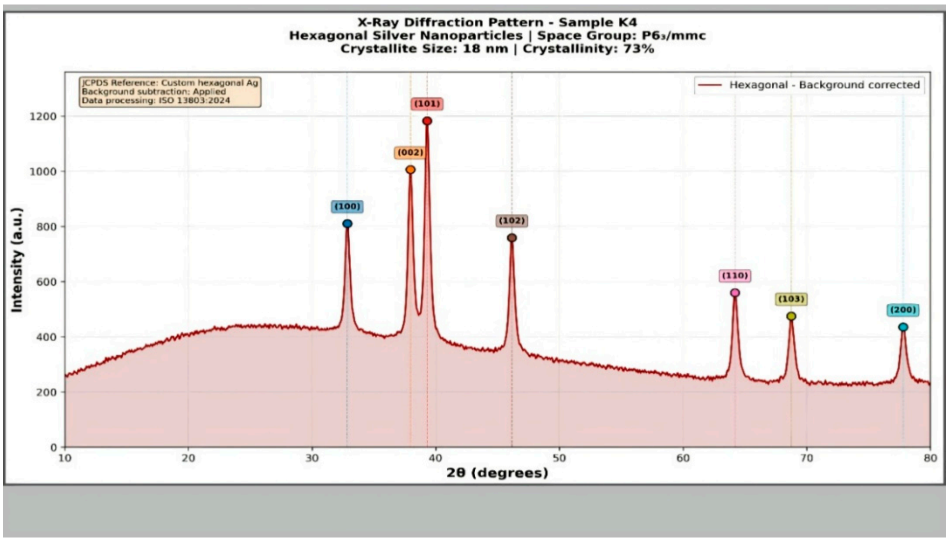

Figure S14. XRD of analysis of Ag NPs synthesized using *Ziziphus* stem extract.

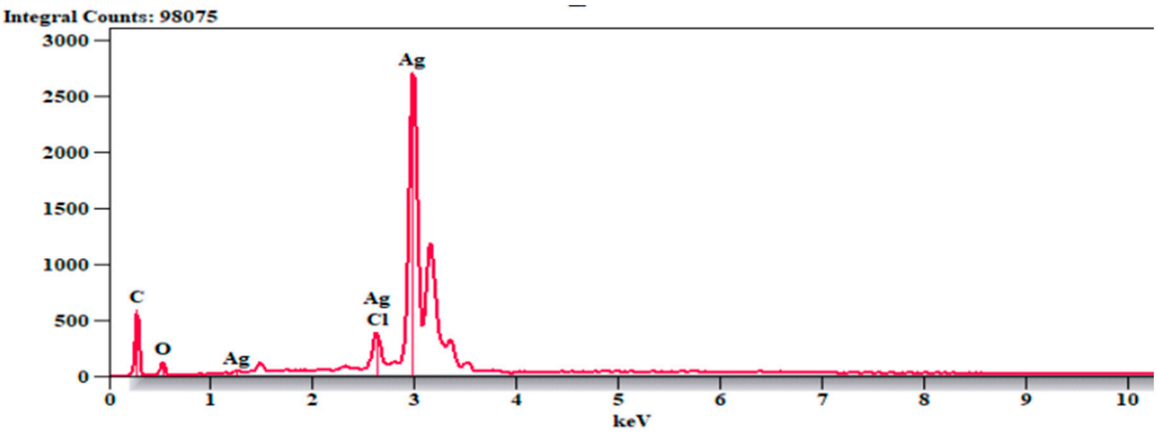

Figure S15. EDX spectrum of AgNPs prepared using *Artemisia* stem extract.

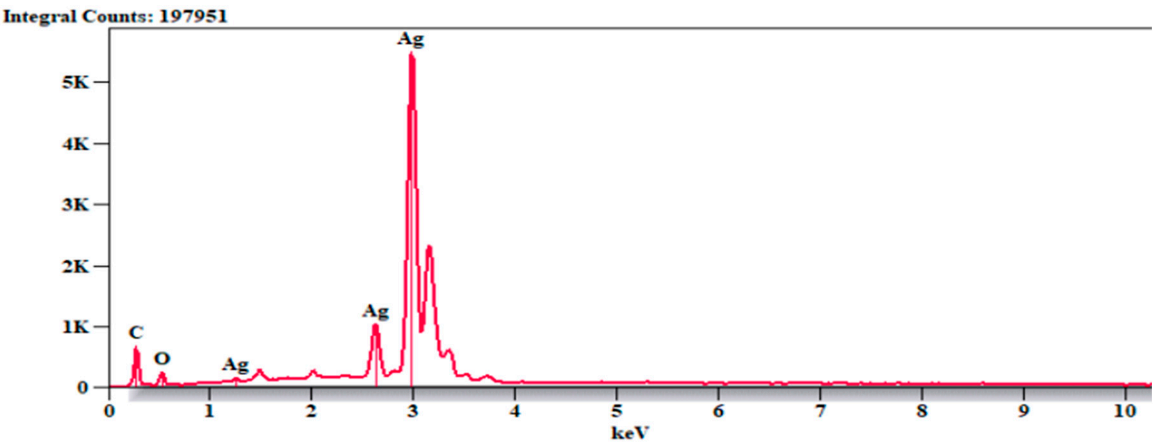

Figure S16. EDX spectrum of AgNPs prepared using *Ziziphus* leaf extract.

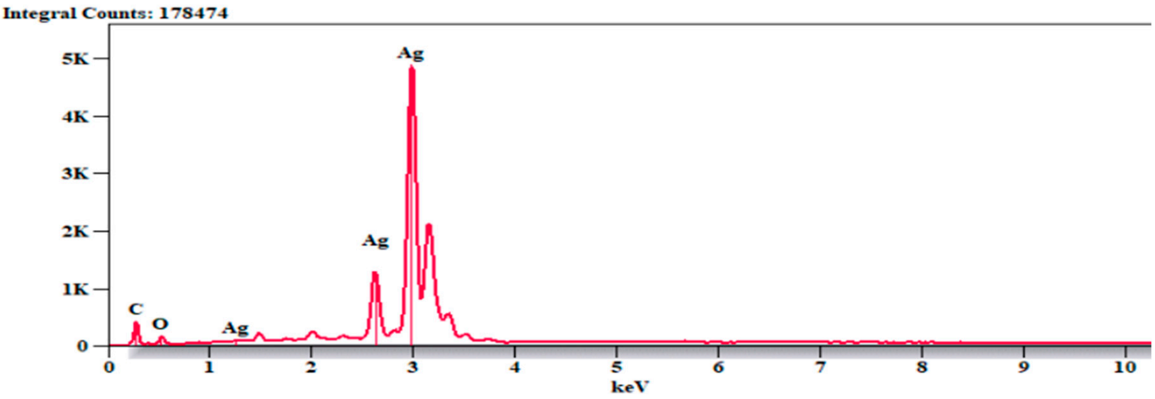

Figure S17. EDX spectrum of AgNPs prepared using *Ziziphus* stem extract.

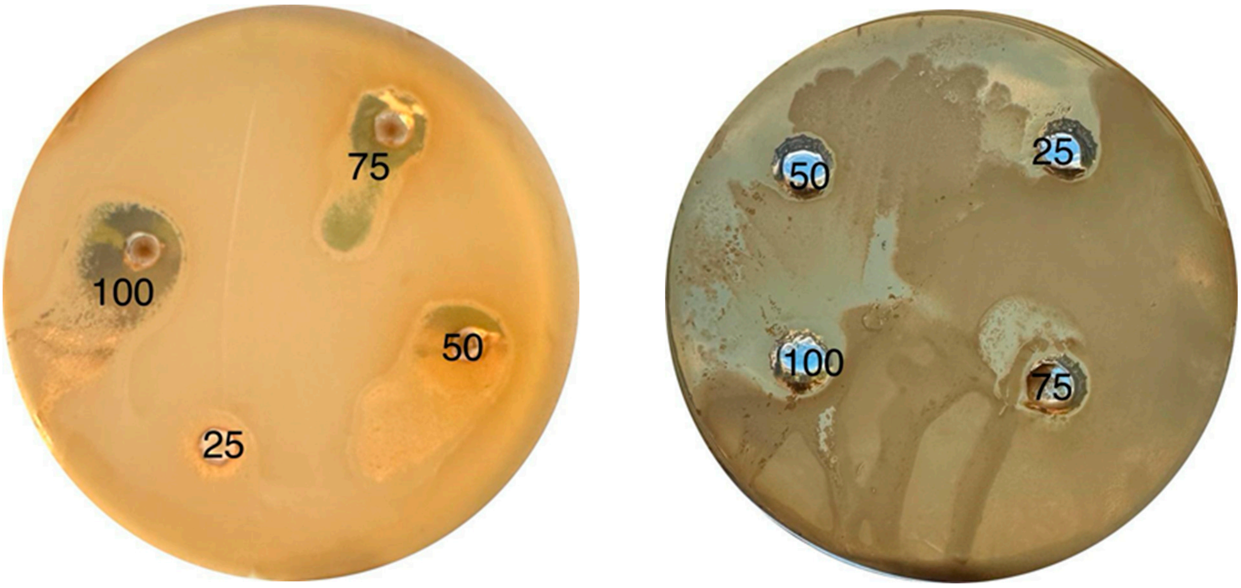

Figure S18. The effect of ***Ziziphus*** **Leaf extract** before adding nanoparticles on Staphylococcus and *E. coli* bacteria.

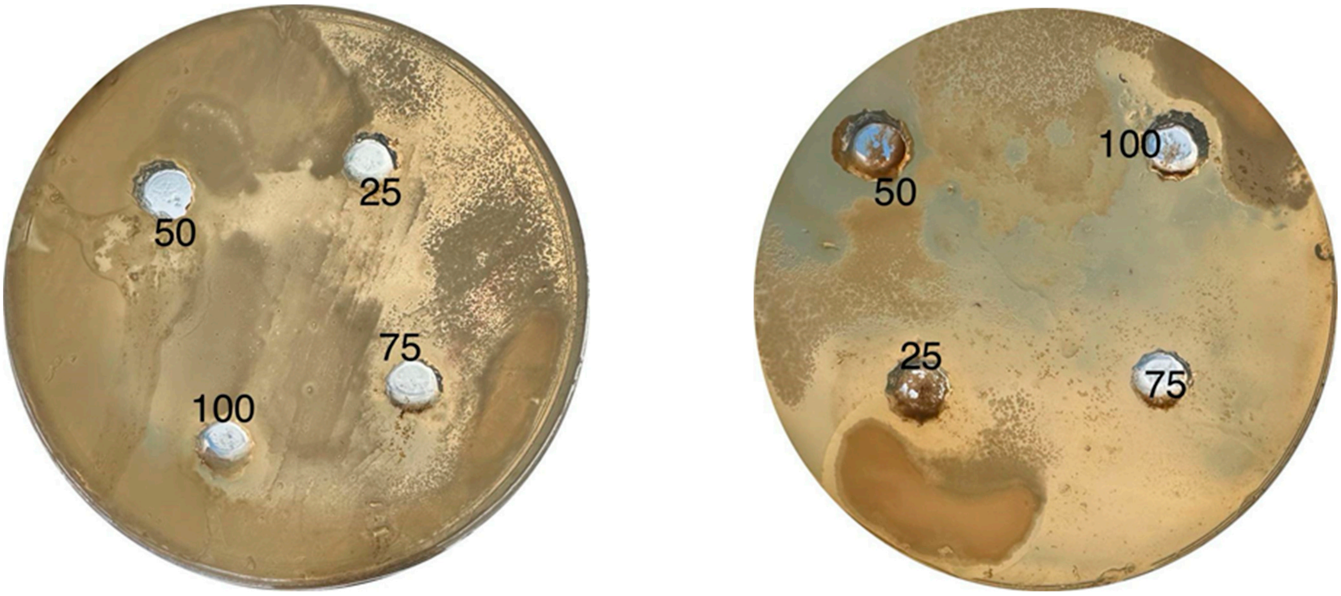

Figure S19. The effect of ***Ziziphus*** **Stems** extracts on *Staphylococcus* and *E. coli* bacteria.

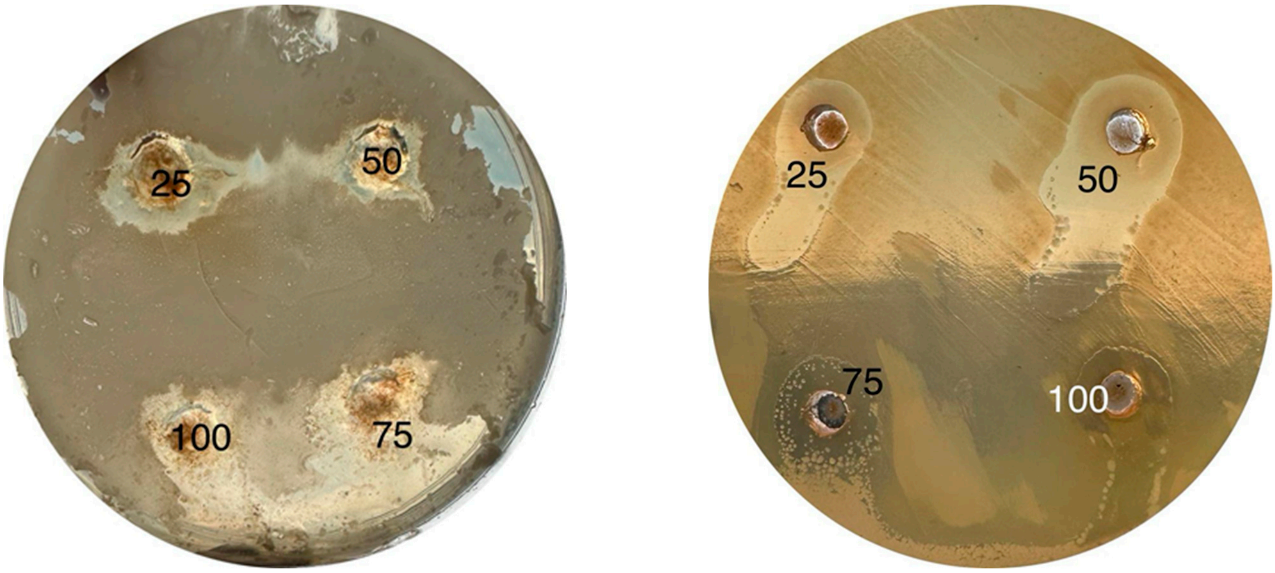

Figure S20. The effect of ***Artemisia*** plant leaf's extracts.

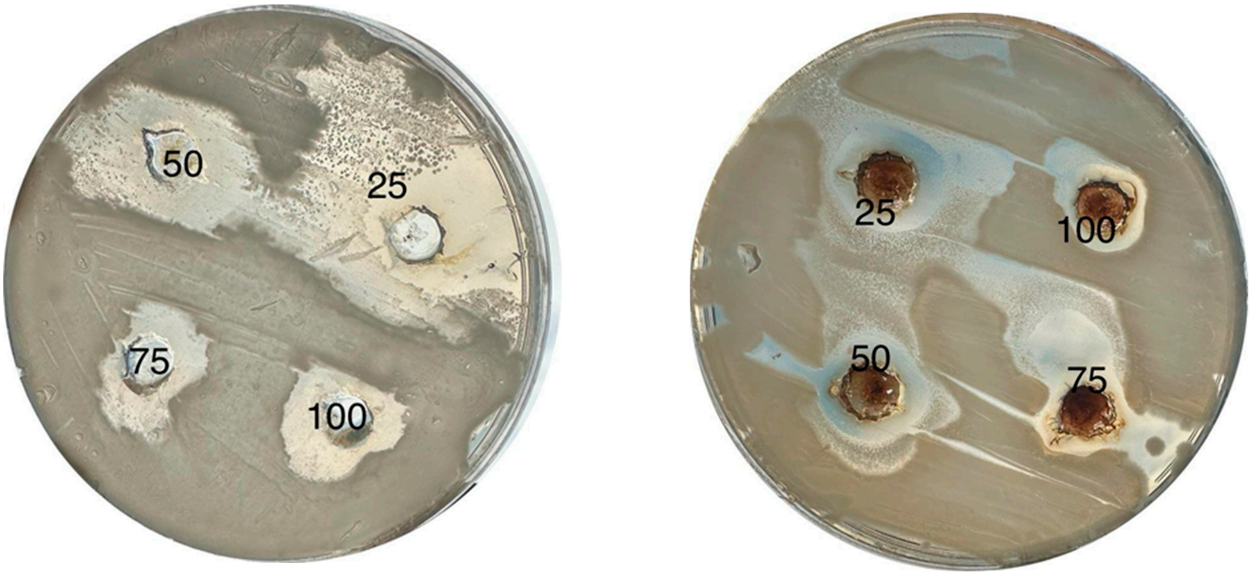

Figure S21. The effect of ***Artemisia*** Stems plant extract on ***Staphylococcus*** and ***E. coli bacteria***.

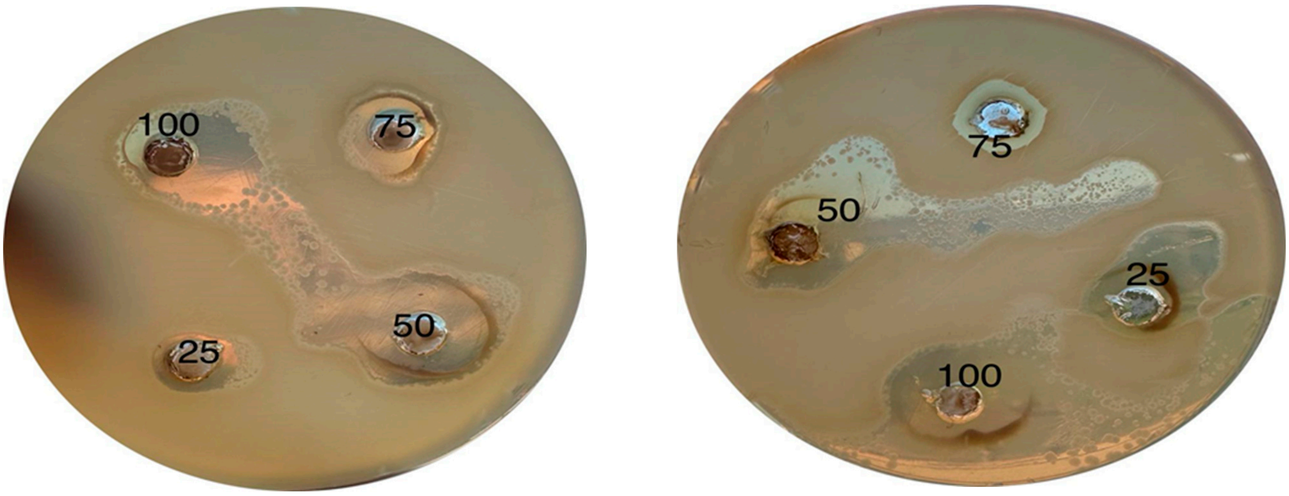

Figure S22. Effect of *Ziziphus* leaf nanoparticles on the studied Bacteria.

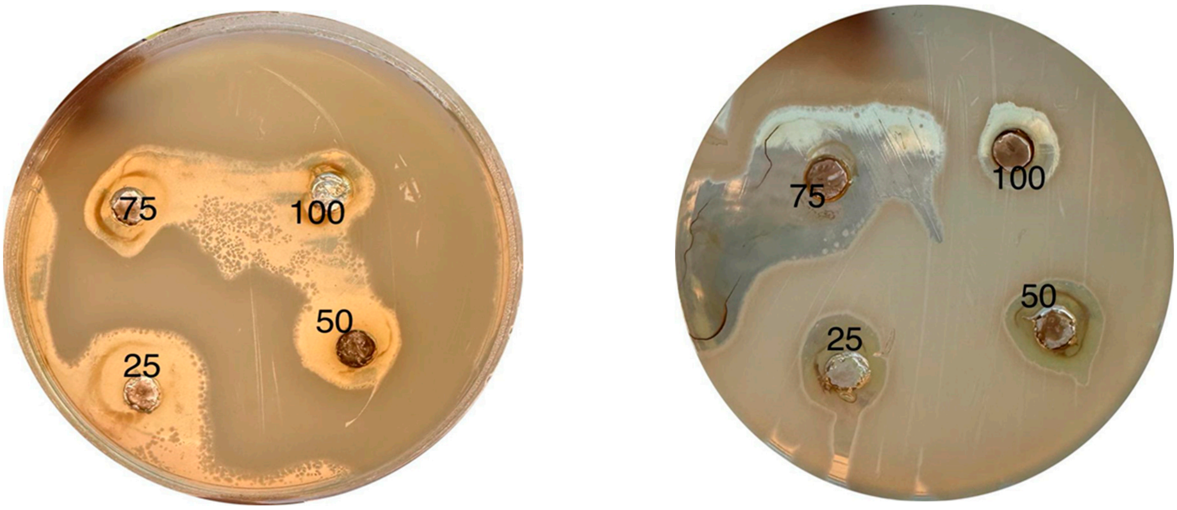

Figure S23. The Effect of stems of *Ziziphus* nanoparticles on the studied Bacteria.

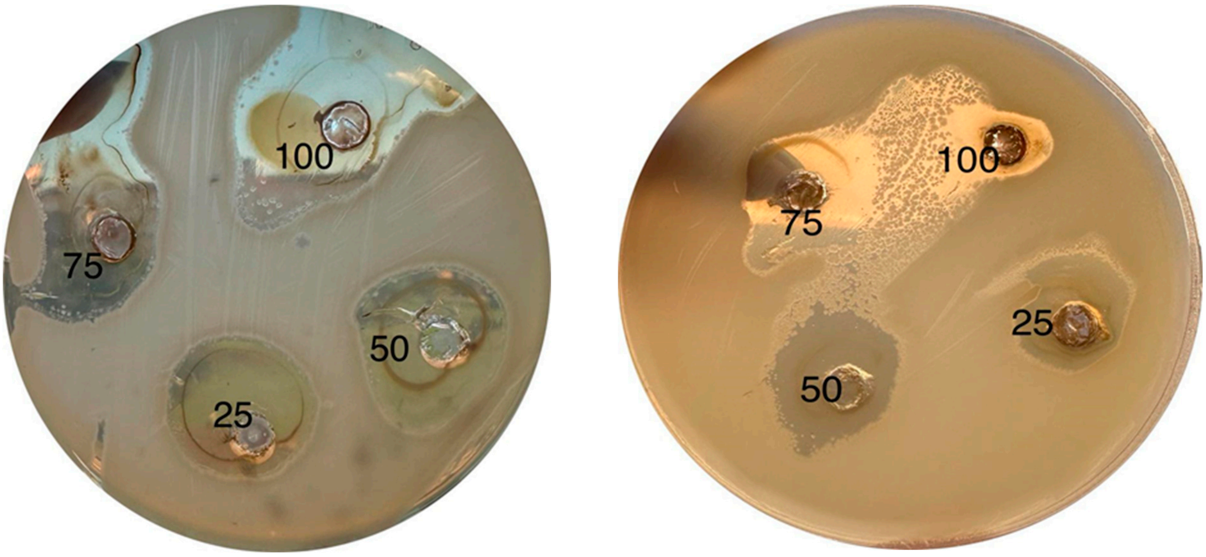

Figure S24. Effect of leaf's ***Artemisia*** nanoparticles on the studied Bacteria**.**

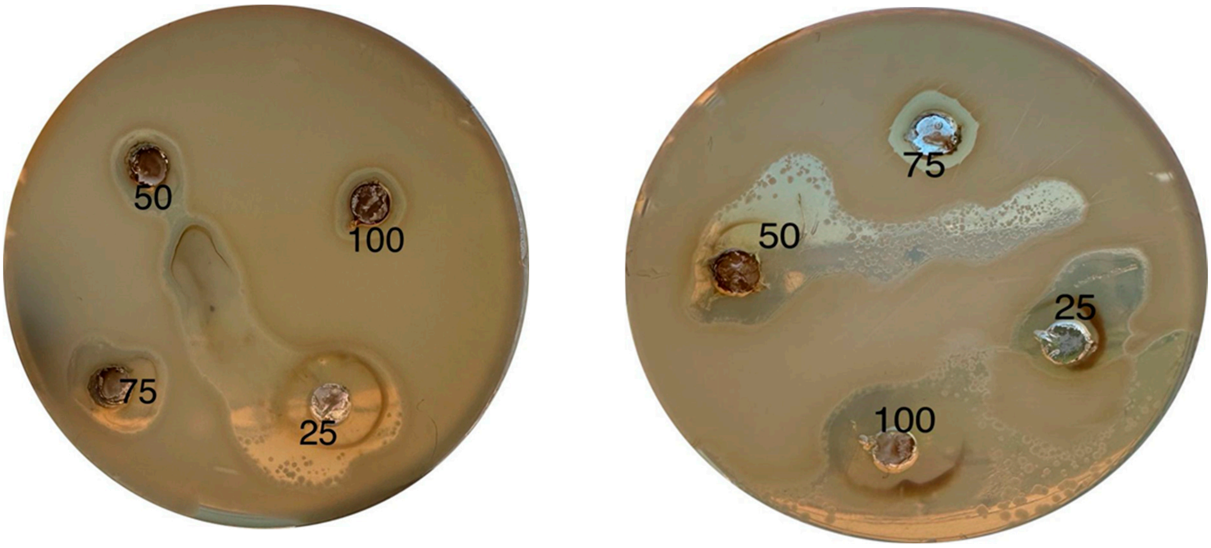

Figure S25. Effect of stems ***Artemisia*** nanoparticles on the studied Bacteria.

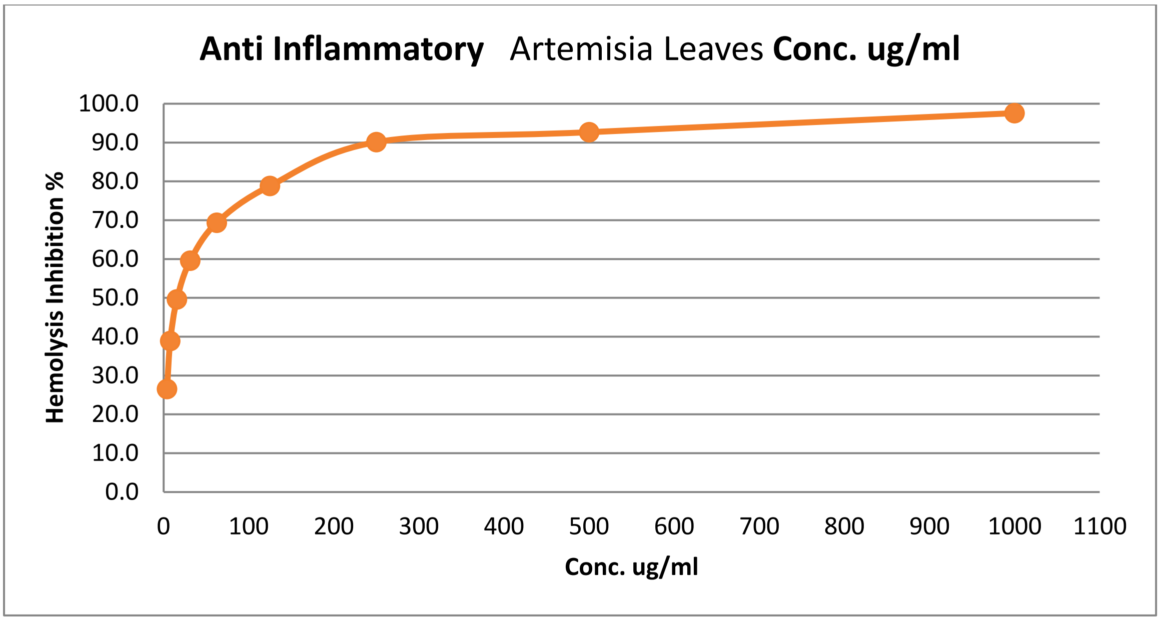

Figure S26. Anti-inflammatory of *Artemisia* leaf's.

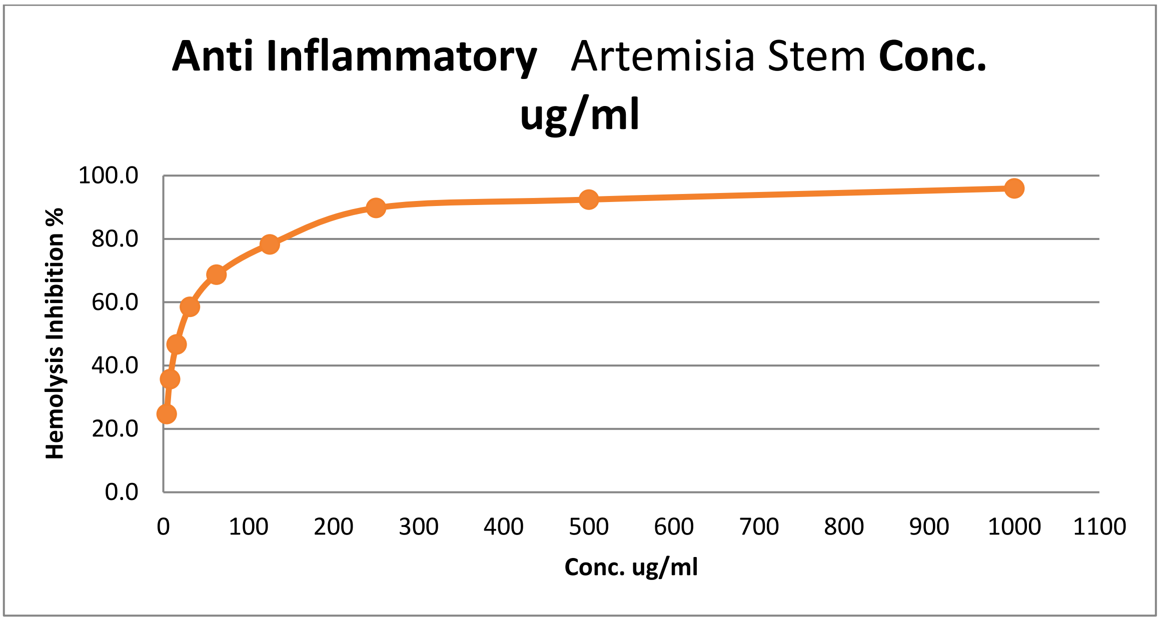

Figure S27. Anti-inflammatory of *Artemisia* stems.

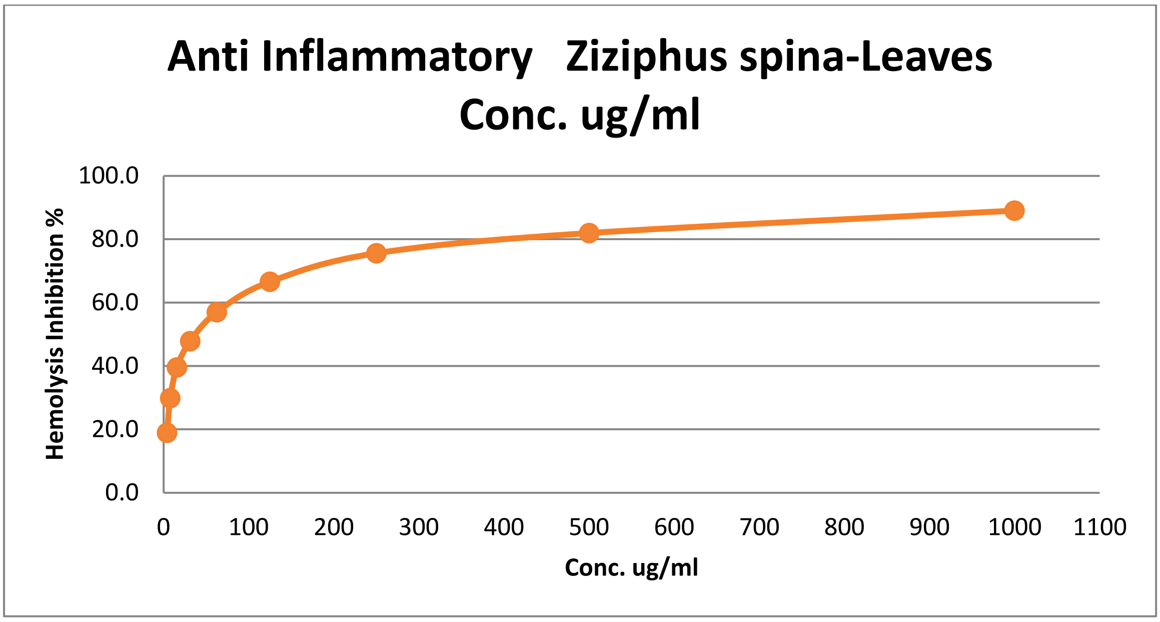

Figure S28. Anti-inflammatory of *Ziziphus* leaf's.

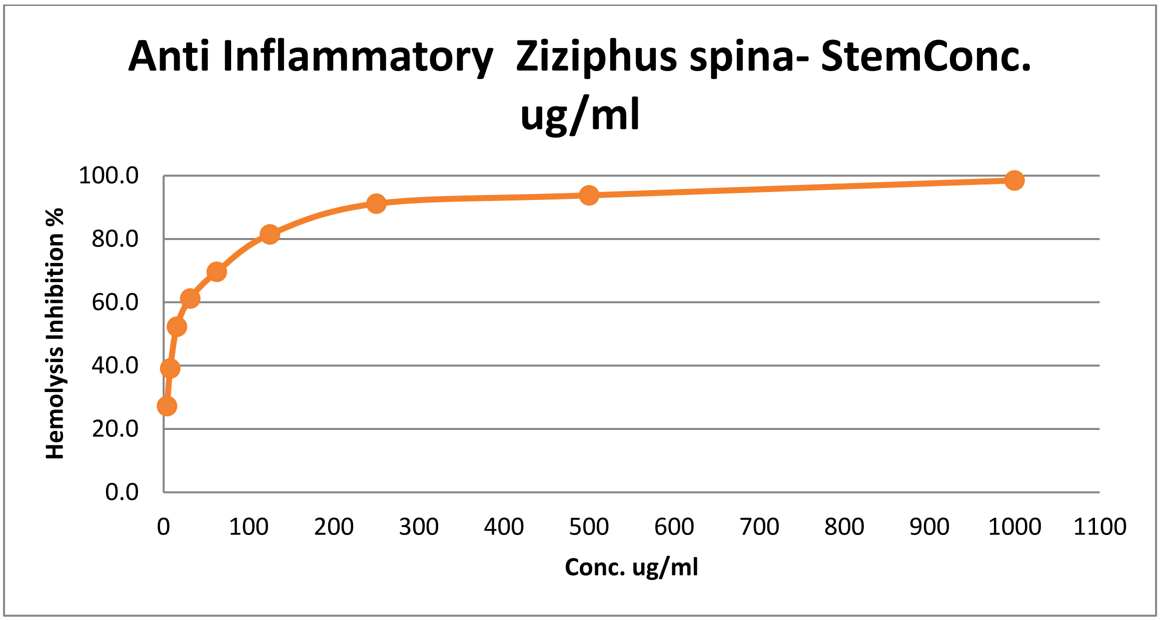

Figure S29. Anti-inflammatory of *Ziziphus* stems.

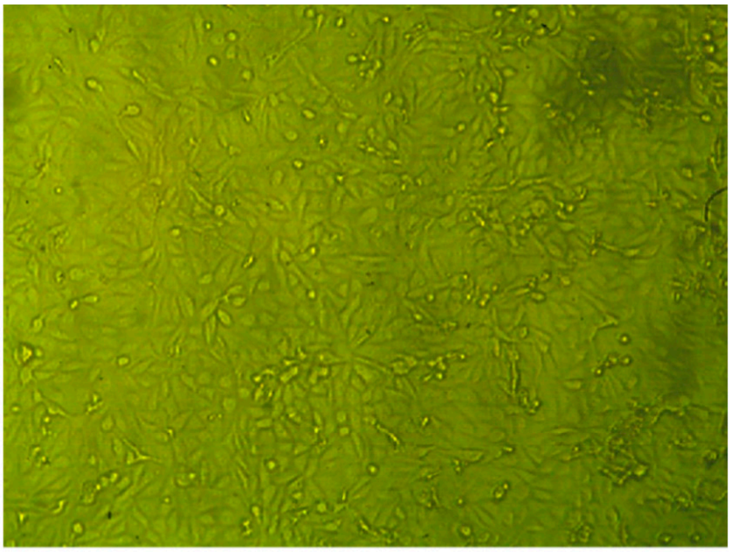

control
Mcf7 cells

Organism : *Homo sapiens*, human
Tissue : mammary gland, breast; derived from metastatic site: pleural effusion
Cell Type : epithelial
Culture Properties : adherent
Disease : adenocarcinoma
ATCC : HTB-22

Figure S30. Control of McF7 Cell.

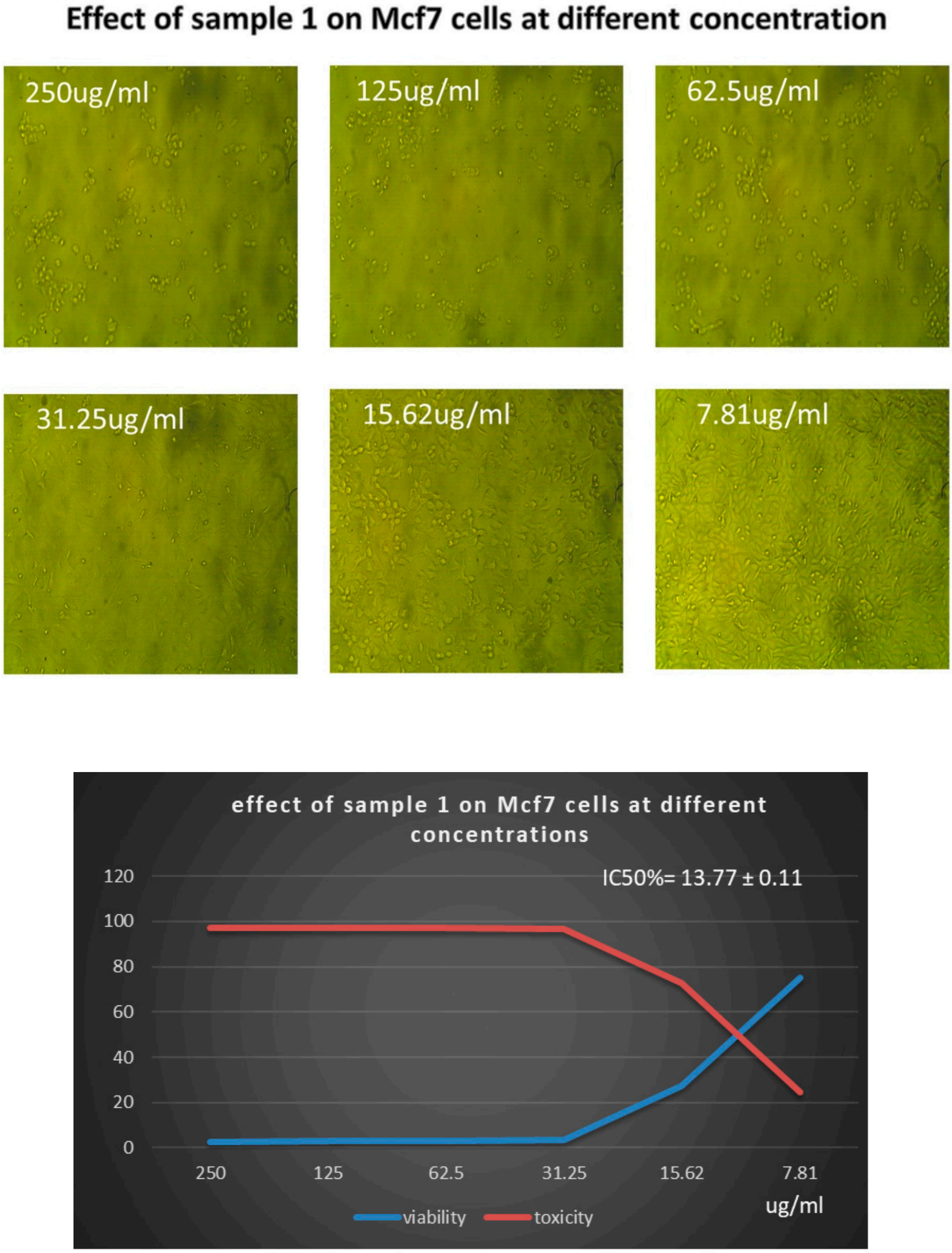

Figure S31. Effect of the sample 1 on Mcf7 cell.

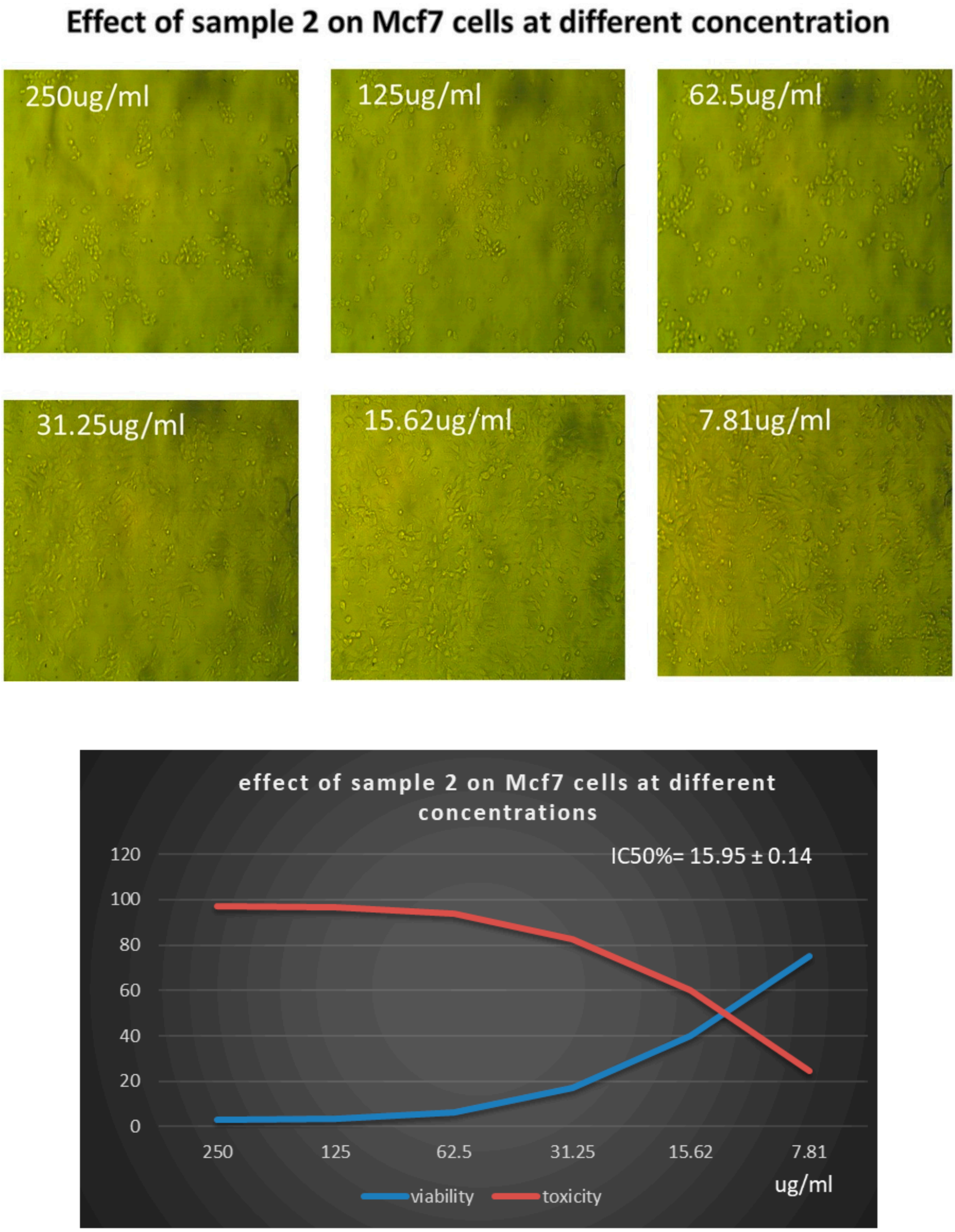

Figure S32. Effect of the sample 2 on Mcf7.

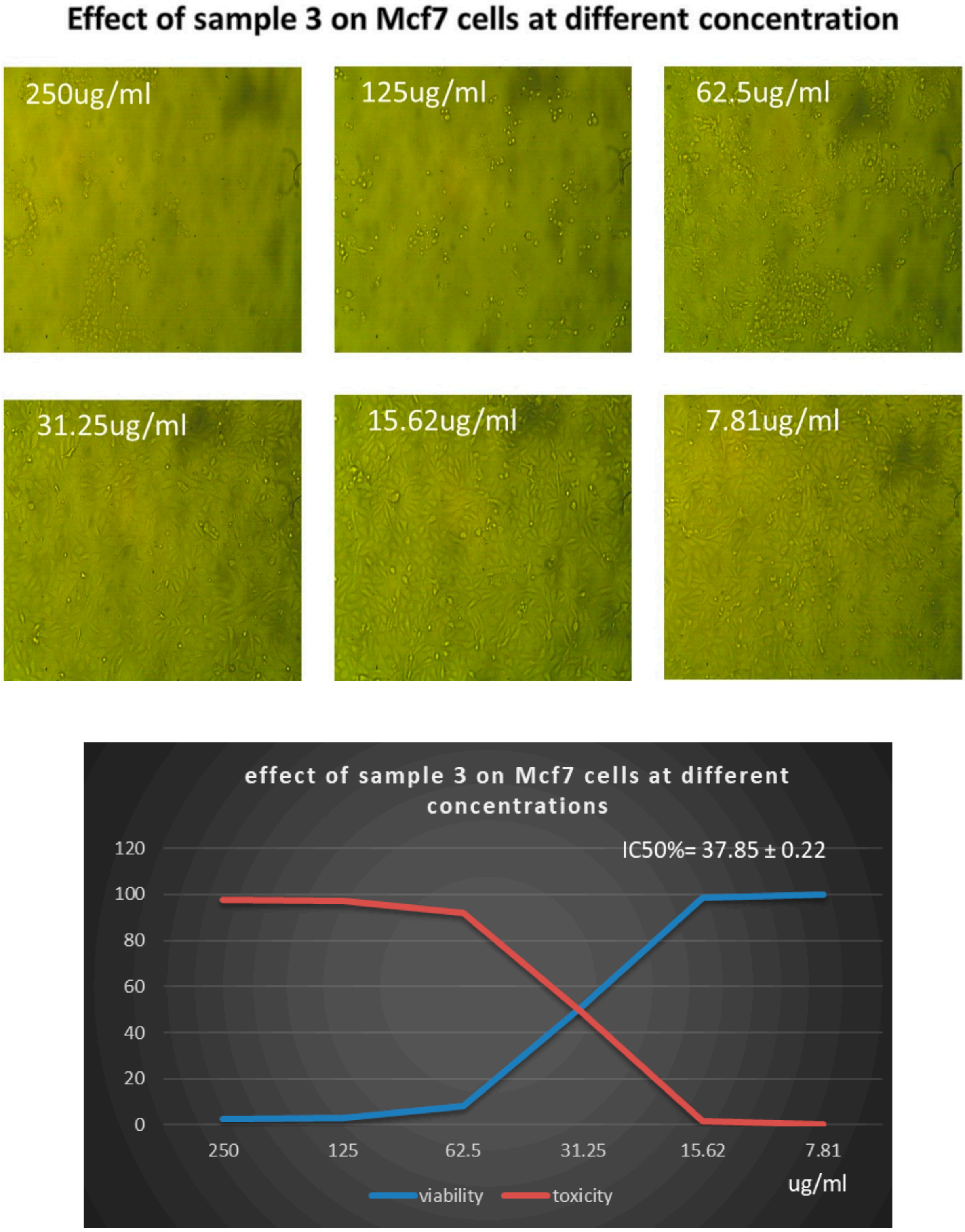

Figure S33. Effect of the sample 3 on Mcf7.

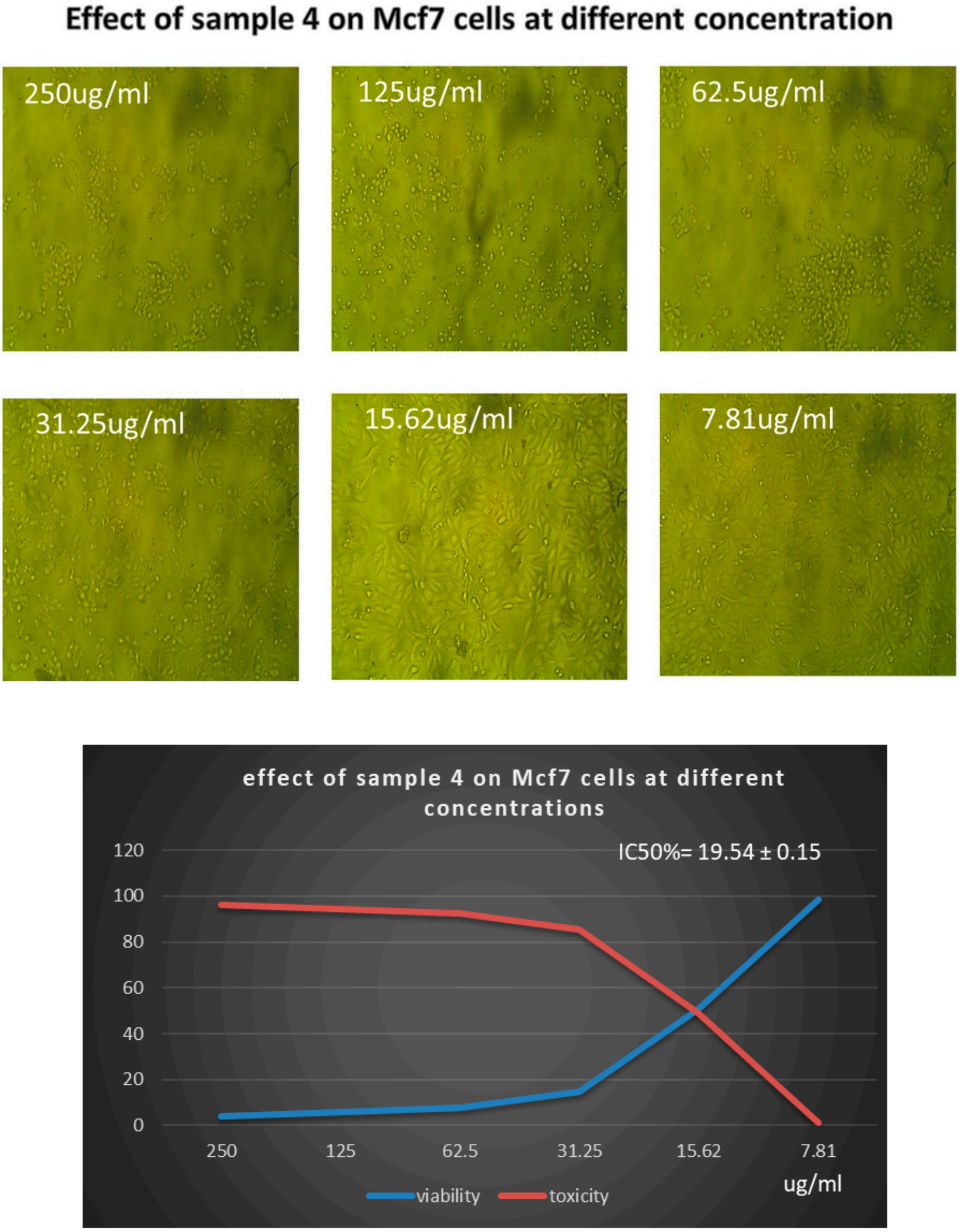

Figure S34. Effect of the sample 4 on Mcf7.

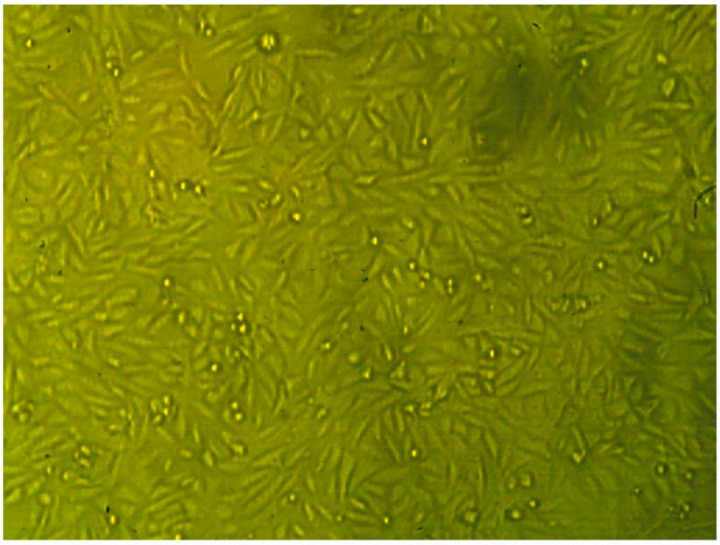

control
HepG2 cells

Organism :	*Homo sapiens*, human
Tissue :	liver
Cell Type :	epithelial
Culture Properties :	adherent
Disease :	hepatocellular carcinoma
ATCC :	HB-8065

Figure S35. The control of HepG2 cells.

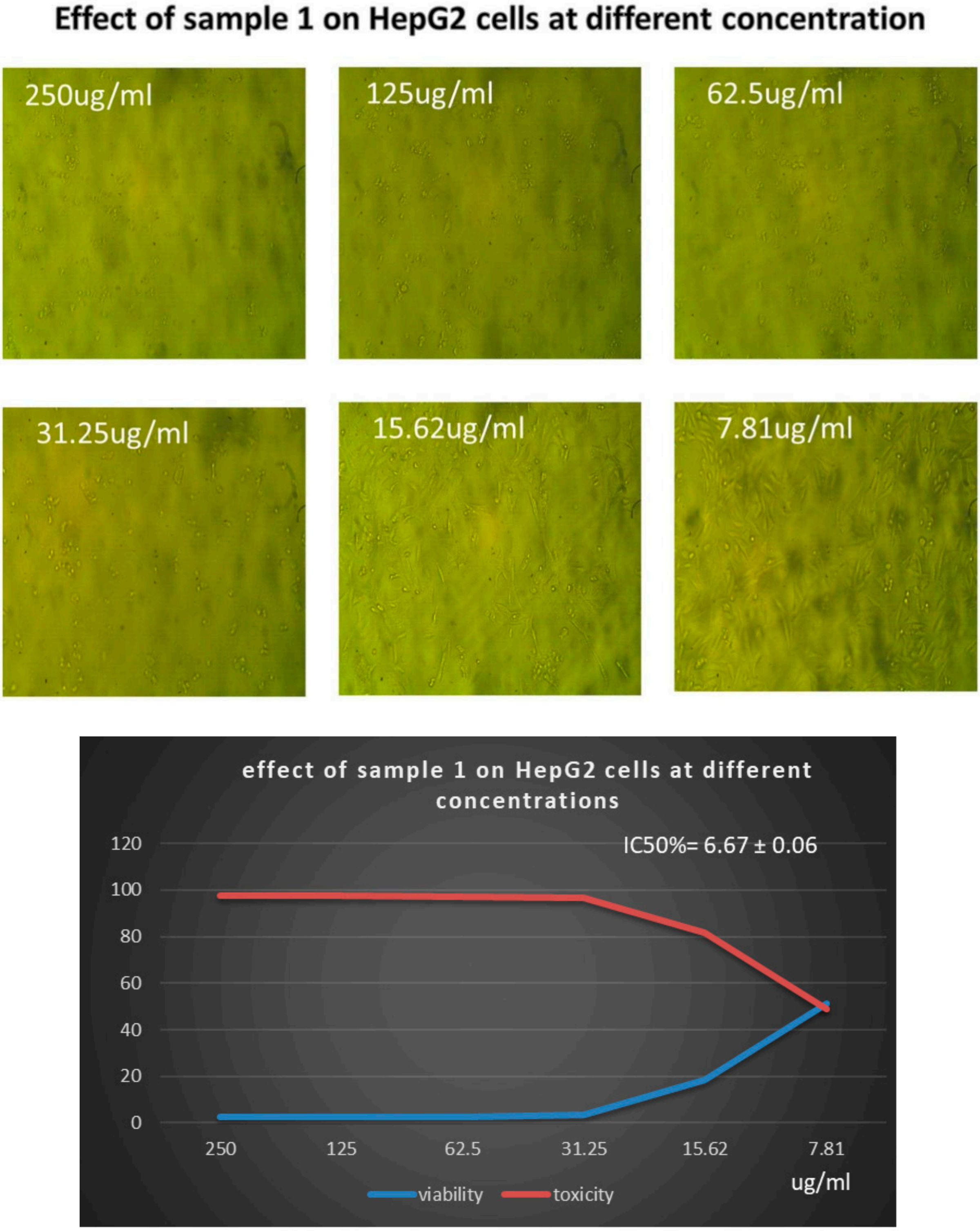

Figure S36. Effect of the sample 1 on HepG2.

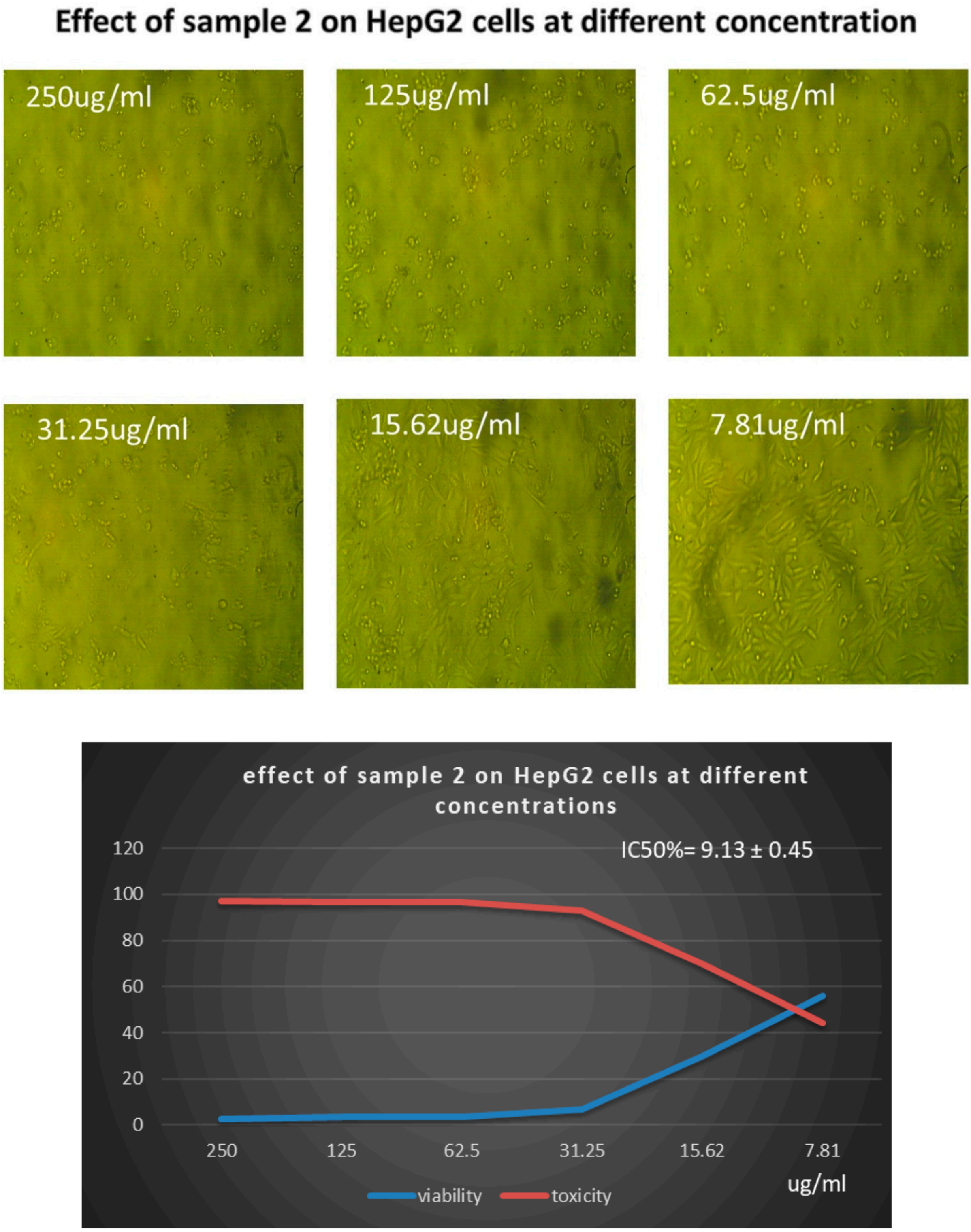

Figure S37. Effect of the sample 2 on HepG2.

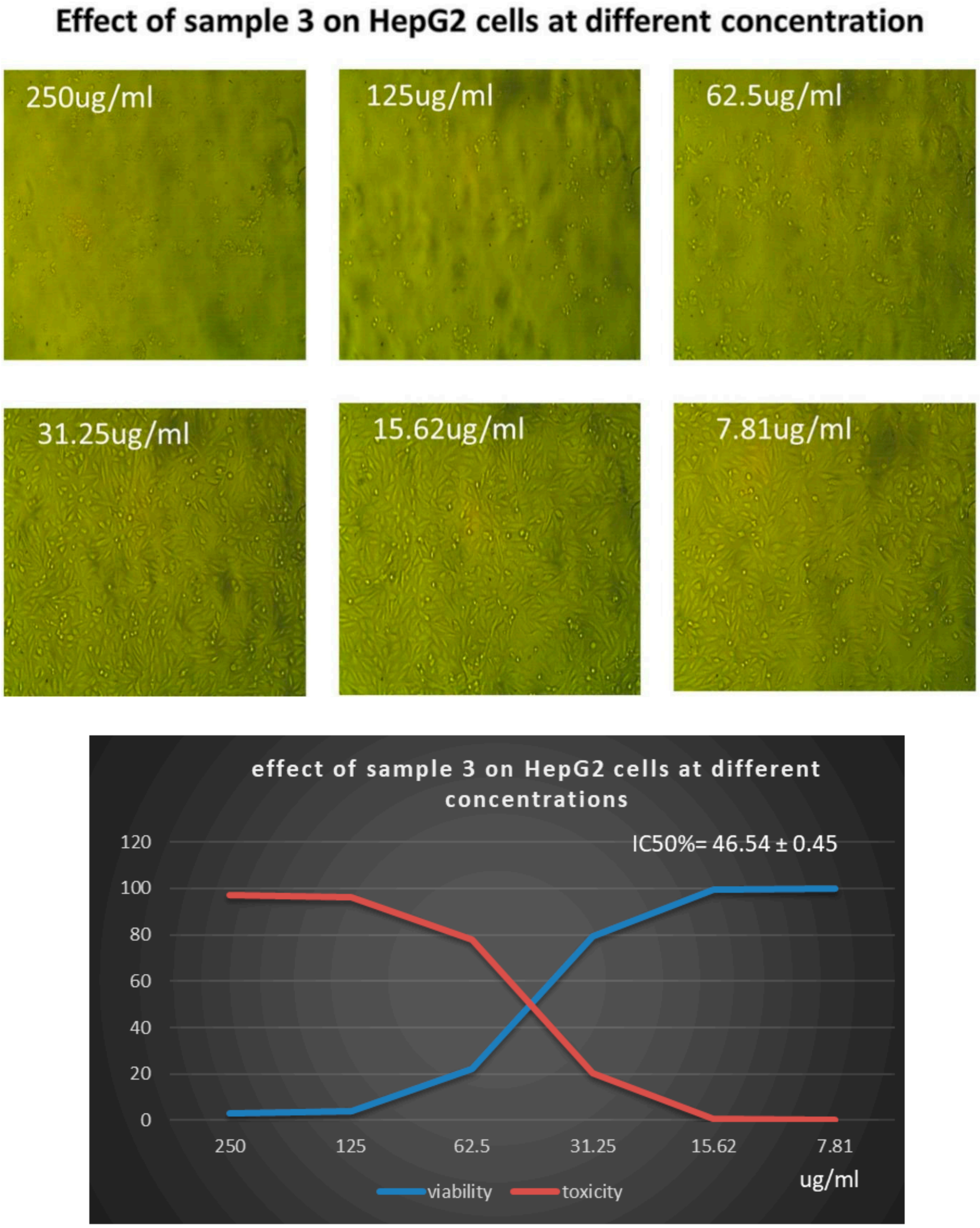

Figure S38. Effect of the sample 3 on HepG2.

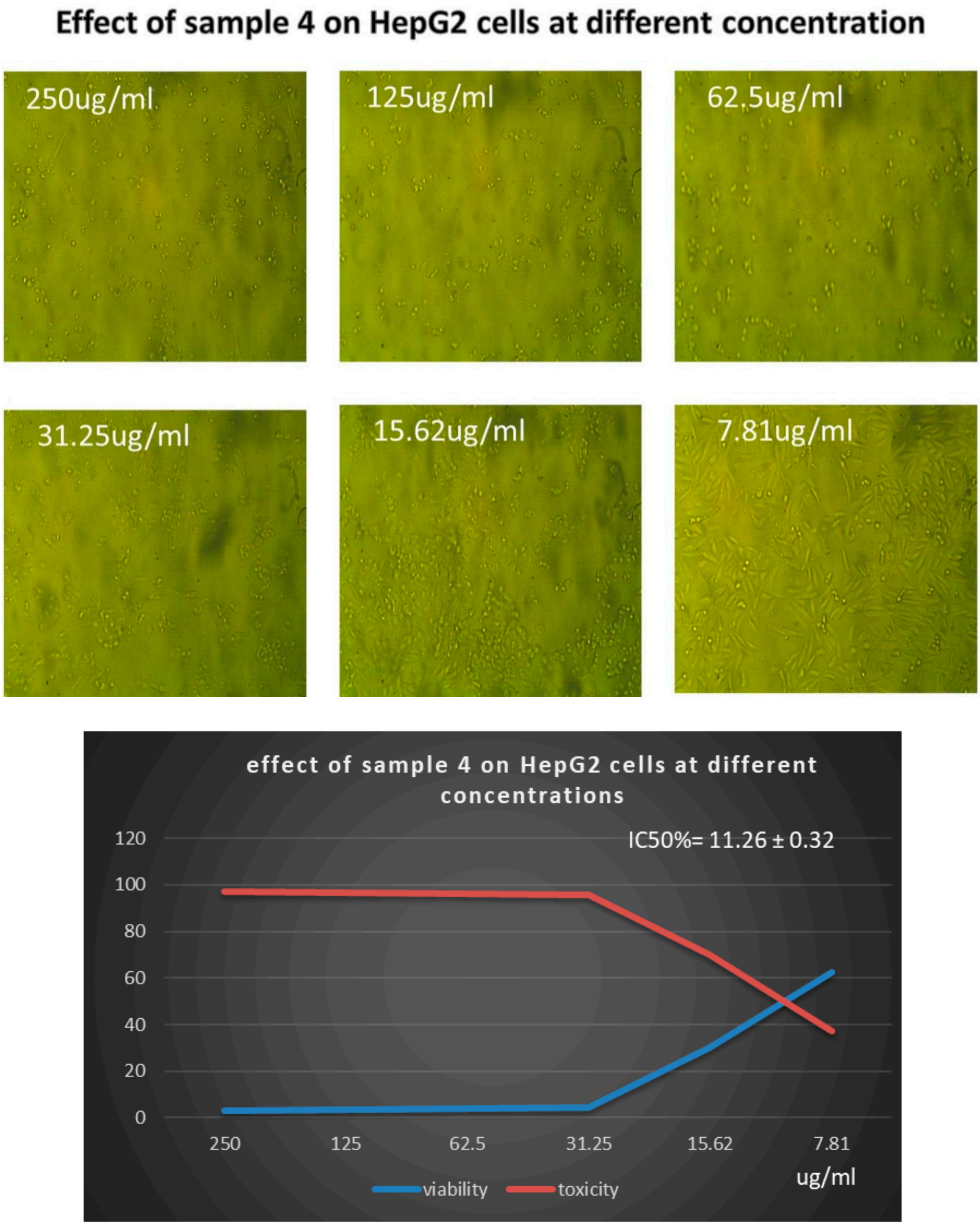

Figure S39. Effect of the sample 4 on HepG2.

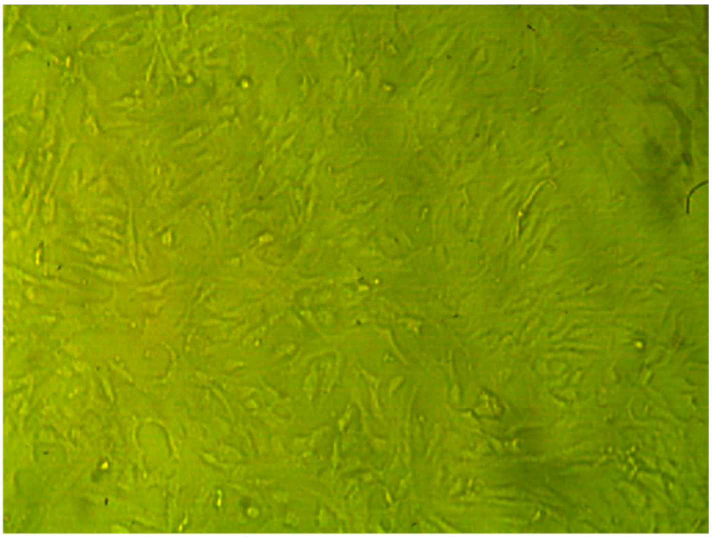

control
A549 cells

Organism : *Homo sapiens*, human
Tissue : lung
Cell Type : epithelial
Culture Properties : adherent
Disease : Carcinoma
ATCC : CCL-185

Figure S40. Control of A549 cells.

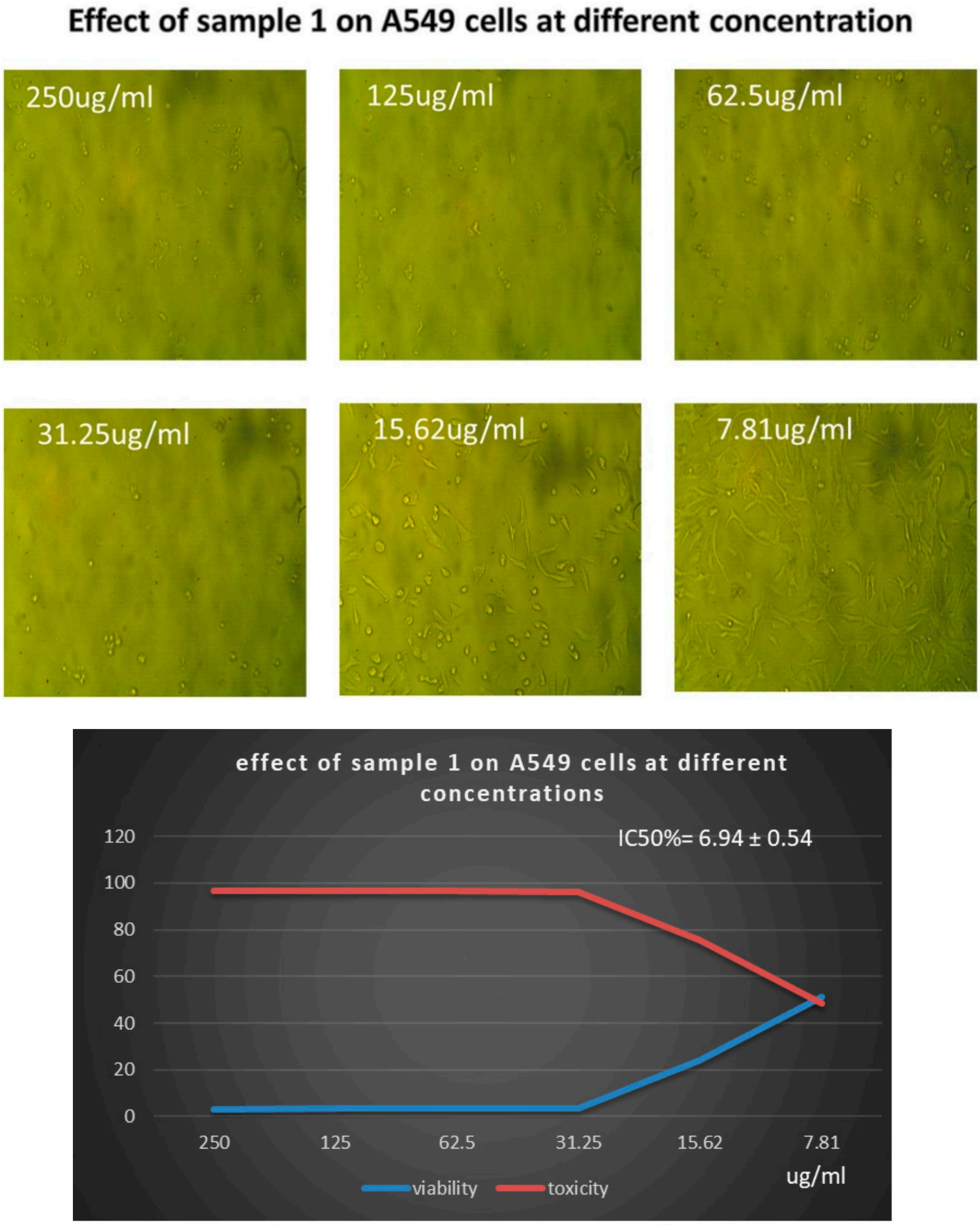

Figure S41. Effect of the sample 1 on A 549 cells.

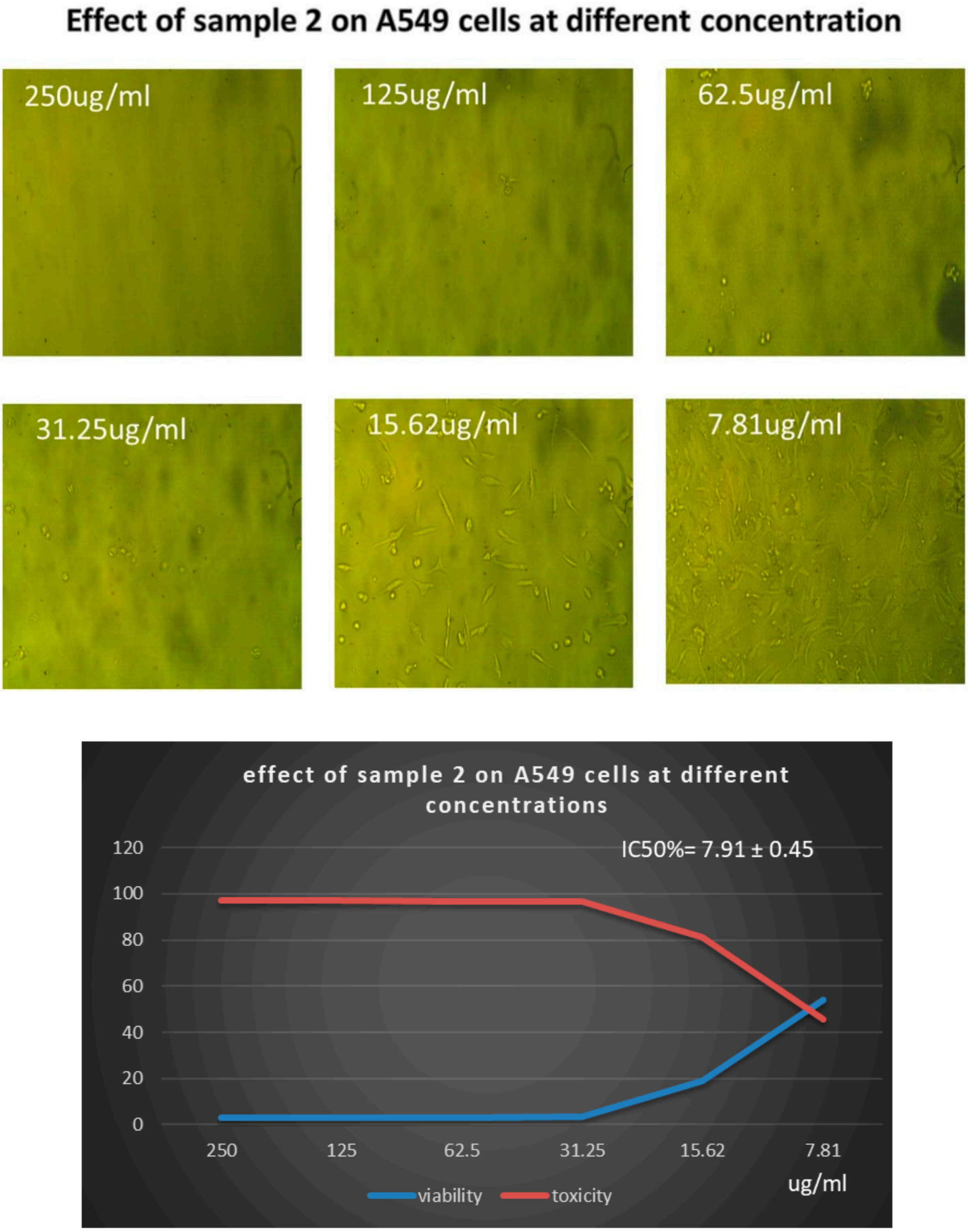

Figure S42. Effect of the sample 2 on A549 cells.

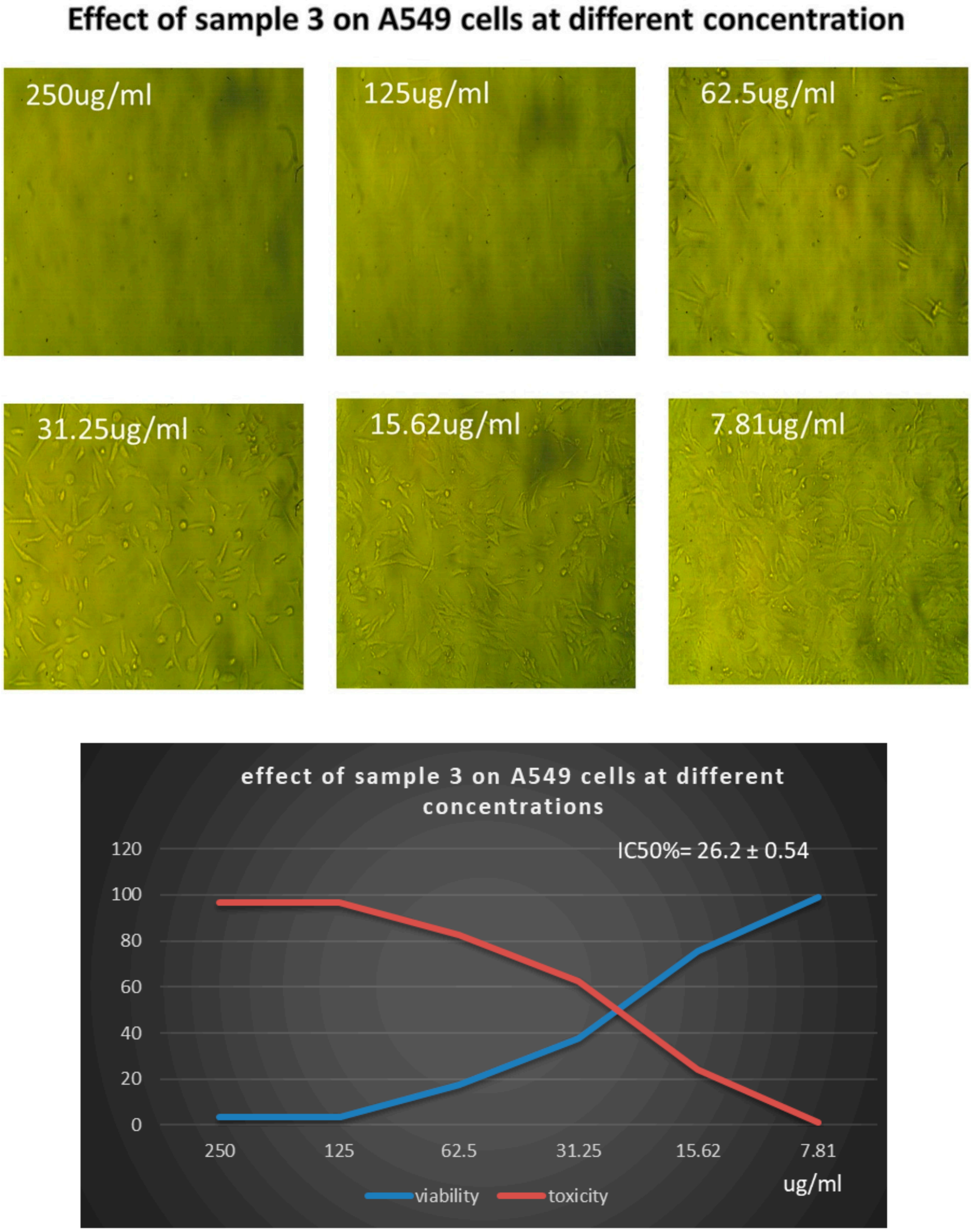

Figure S43. Effect of the sample 3 on A549 cells.

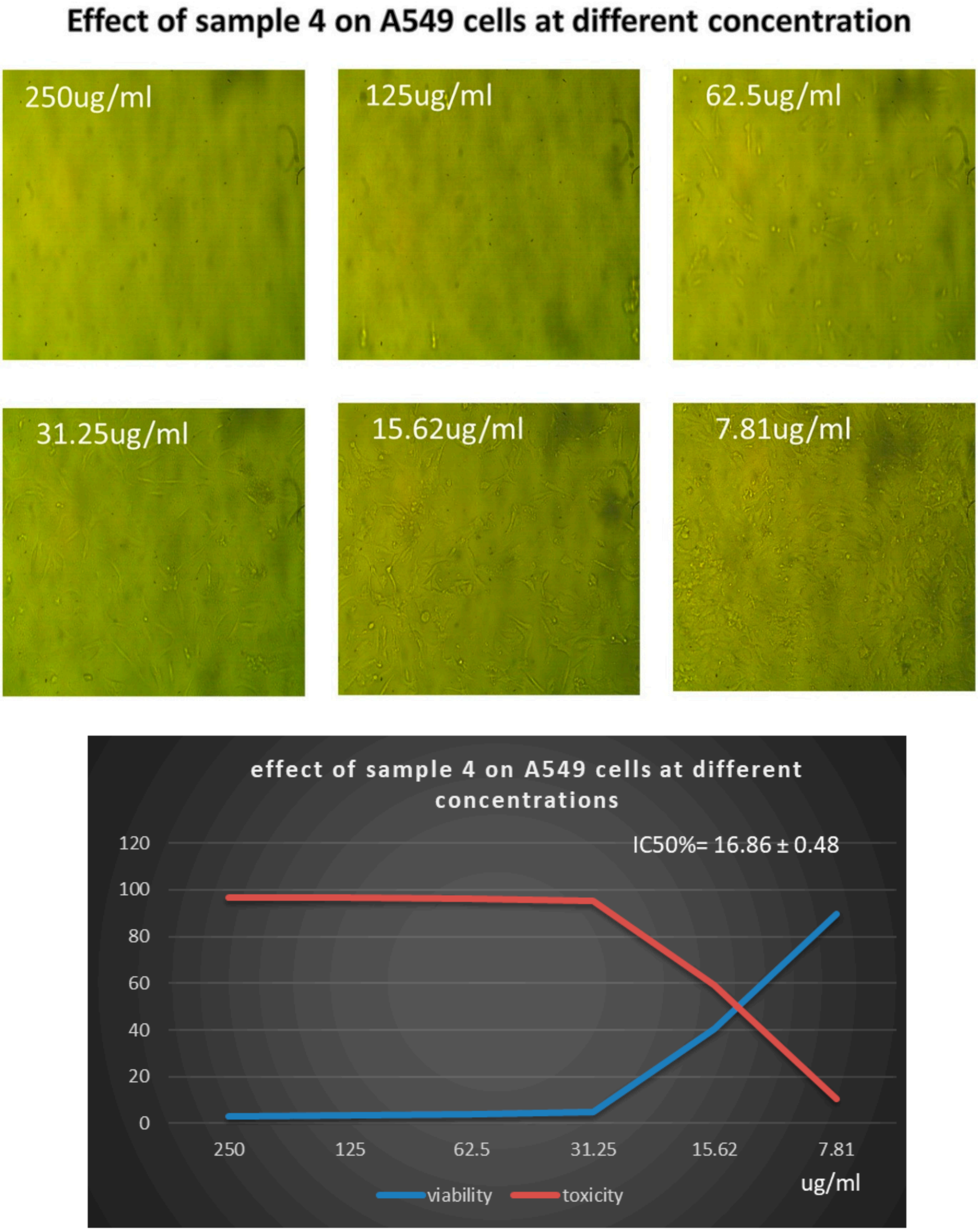

Figure S44. Effect of the sample 4 on A549 cells.

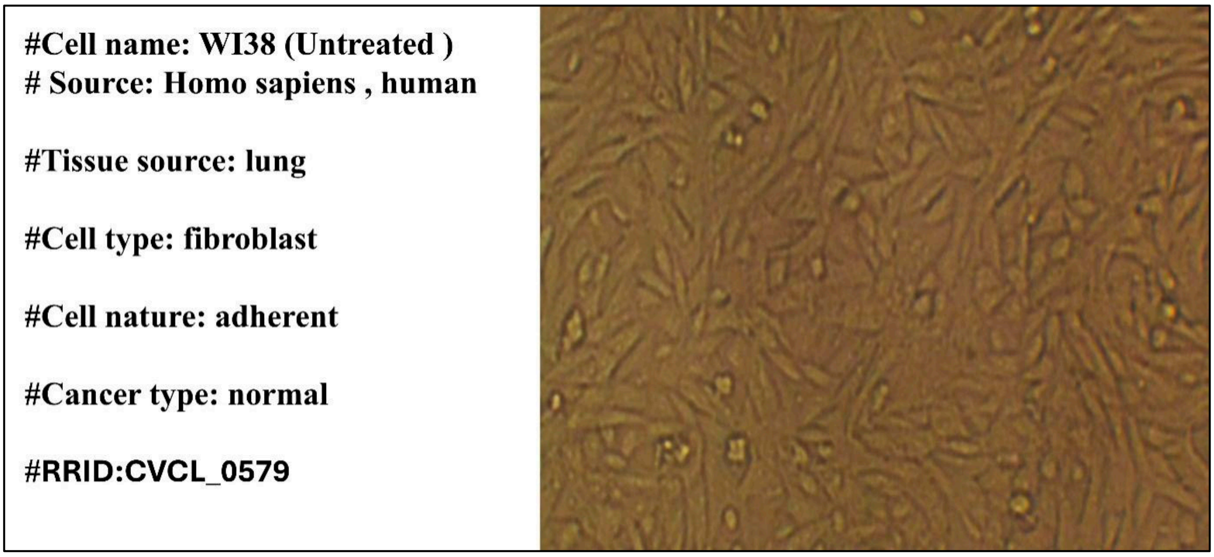

Figure S45. Control of WI38 cells.

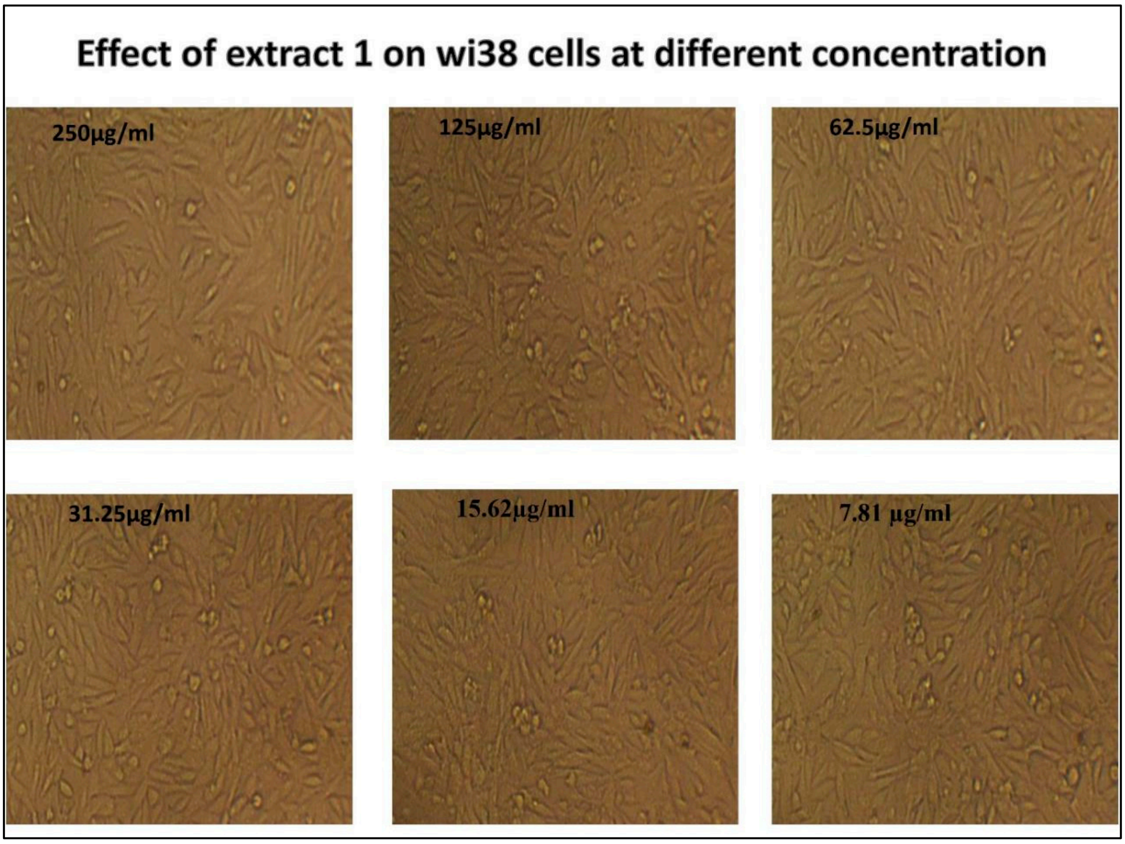

Figure S46. effect of extract 1 on WI38 cells.

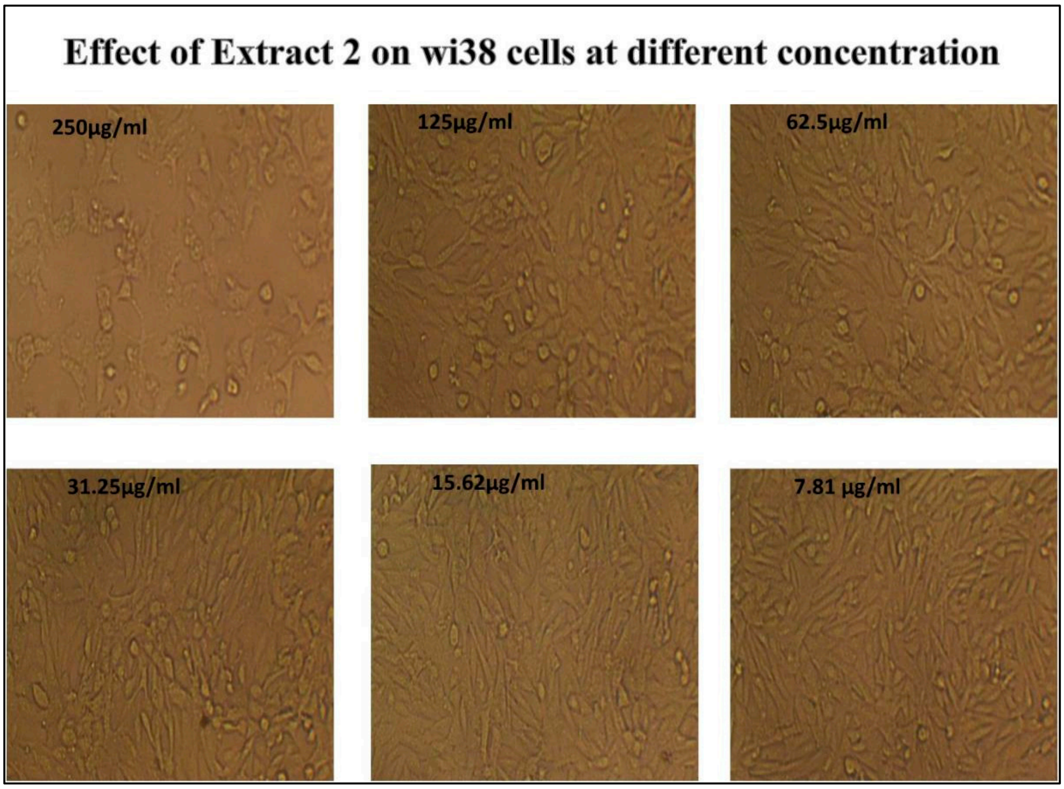

Figure S47. effect of extract 2 on WI38 cells.

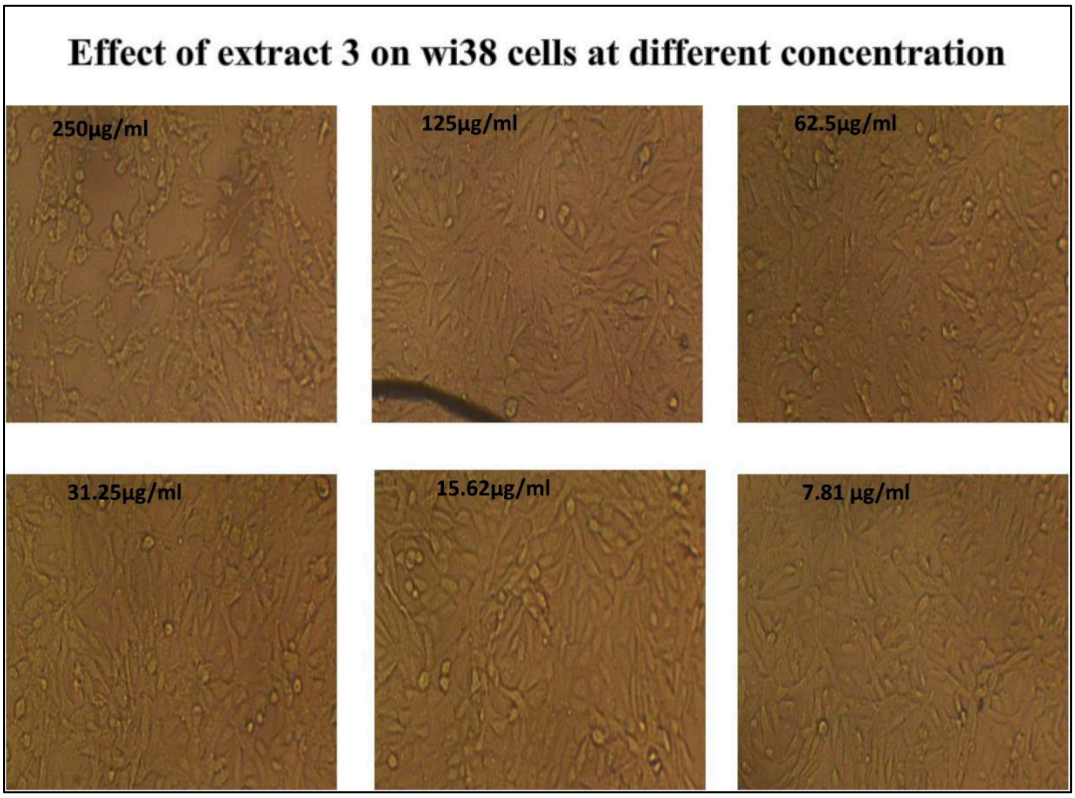

Figure S48. effect of extract 3 on WI38 cells.

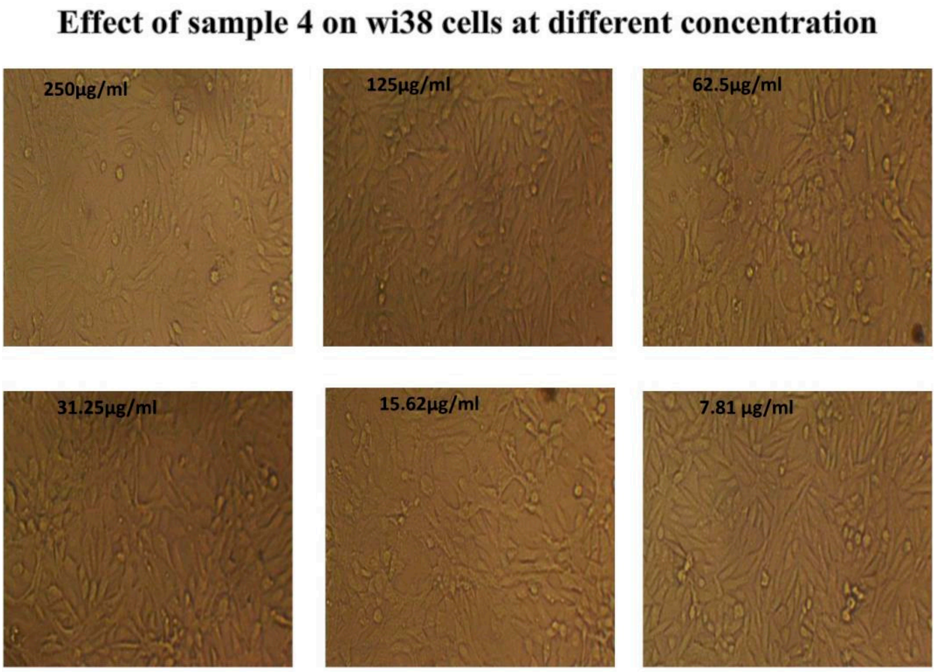

Figure S49. effect of extract 4 on WI38 cells.

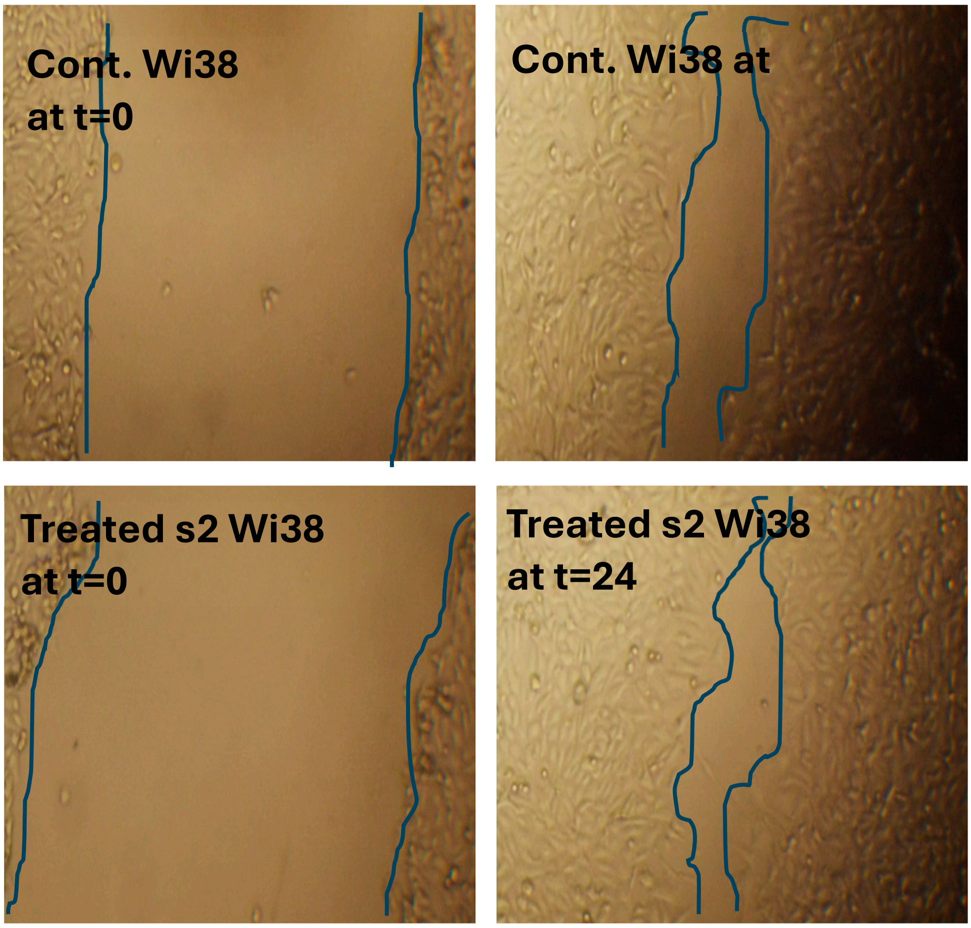

Figure S50. Scratch wound healing assay of Wi-38 fibroblasts treated with Sample 2.

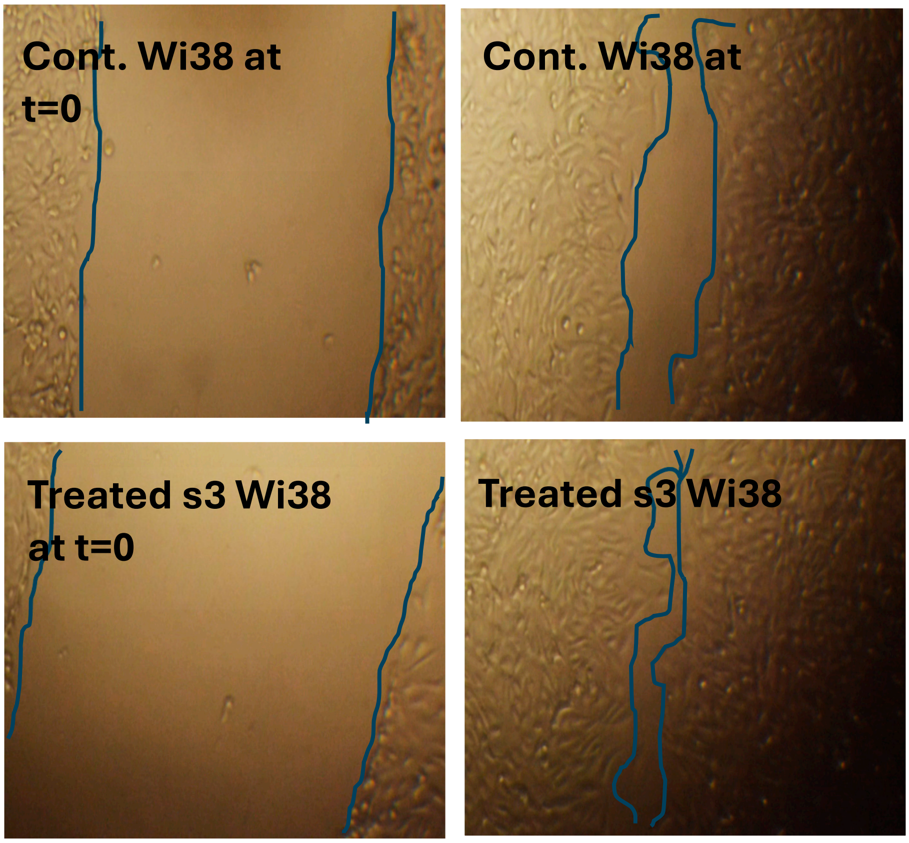

Figure S51. Scratch wound healing assay of Wi-38 fibroblasts treated with Sample 3.

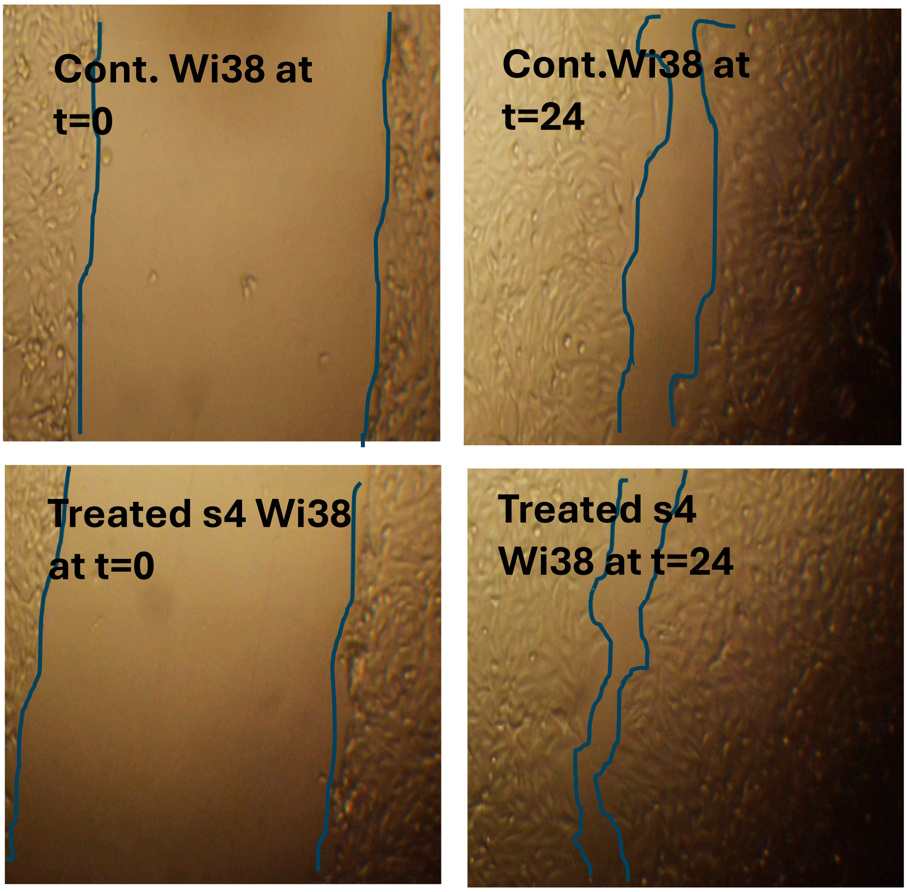

Figure S52. Scratch wound healing assay of Wi-38 fibroblasts treated with Sample 4.

Table S1. The effect of ***Ziziphus*** **Stems** extract before adding nanoparticles.

Concentration (mg mL^{-1})	Inhibition Zone (mm) E. coli	inhibition Zone (mm) Staphylococcus
25	5	3
50	4	8
75	9	9
100	3	8

Table S2. The effect of ***Artemisia*** Stems extract before adding nanoparticles.

Concentration (mg mL^{-1})	Inhibition Zone (mm) E. coli	Inhibition Zone (mm) Staphylococcus
25	7	10
50	5	7
75	4	6
100	3	4

Table S3. Effect of leaf's of *Ziziphus* nanoparticles on the studied Bacteria.

Concentration (mg mL^{-1})	inhibition Zone (mm) E. coli	inhibition Zone (mm) Staphylococcus
25	4	8
50	10	9
75	5	5
100	8	8

Table S4. Effect of stems *Ziziphus* nanoparticles on the studied Bacteria.

Concentration (mg mL^{-1})	inhibition Zone (mm) E. coli	inhibition Zone (mm) Staphylococcus
25	7	5
50	6	10
75	16	13
100	5	11

Table S5. Effect of leaf's ***Artemisia*** nanoparticles on the Bacteria studied.

Concentration (mg mL^{-1})	inhibition Zone (mm) E. coli	inhibition Zone (mm) Staphylococcus
25	9	7
50	11	9
75	12	8
100	13	13

Table S6. Effect of stems ***Artemisia*** nanoparticles on the Bacteria studied.

Concentration (mg mL^{-1})	Inhibition Zone (mm) E. coli	Inhibition Zone (mm) Staphylococcus
25	9	12
50	7	9
75	6	8
100	5	6

Table S7. Detailed results for Minimal inhibitory concentration (MIC) and minimal bactericidal concentration (MBC) for the nanoparticles extract of the four plant samples.

Minimal inhibitory concentration (MIC) and minimal bactericidal concentration (MBC)							
code	conc. (μg mL⁻¹)	OD1	OD2	OD3	Blank	Reading	MIC (μg mL⁻¹)
S1 St. aureus	1000	0.054	0.05	0.05	0.05	0.001	62.5
	500	0.03	0.039	0.04	0.035	0.001	
	250	0.025	0.033	0.031	0.028	0.002	
	125	0.023	0.026	0.03	0.02	0.006	
	62.5	0.025	0.02	0.018	0.014	0.007	
	31.25	0.129	0.153	0.147	0.009	0.134	
	15.62	0.855	0.863	0.921	0.007	0.873	
	7.81	1.94	1.837	1.893	0.002	1.888	
	3.91	2.532	2.492	2.71	0.001	2.577	
	1.95	2.992	3.018	3.103	0	3.038	
	0.98	3.714	3.951	3.644	0	3.77	
	0.49	4.134	4.324	4.245	0	4.234	
code	conc. (μg mL⁻¹)	OD1	OD2	OD3	Blank	Reading	MIC (μg mL⁻¹)
S2 St. aureus	1000	0.048	0.05	0.043	0.047	0	125
	500	0.035	0.031	0.028	0.03	0.001	
	250	0.027	0.02	0.024	0.023	0.001	
	125	0.034	0.03	0.021	0.017	0.011	
	62.5	0.153	0.173	0.18	0.011	0.158	
	31.25	0.533	0.57	0.58	0.008	0.553	
	15.62	1.288	1.319	1.258	0.003	1.285	
	7.81	2.023	2.103	2.187	0.001	2.103	
	3.91	2.991	3.123	3.103	0.001	3.071	
	1.95	3.184	3.116	3.107	0.001	3.135	
	0.98	4.308	4.106	Over	0	--------	
	0.49	Over	Over	Over	0	--------	
code	conc. (μg mL⁻¹)	OD1	OD2	OD3	Blank	Reading	MIC (μg mL⁻¹)
S3 St. aureus	1000	0.044	0.045	0.041	0.043	0	31.25
	500	0.031	0.02	0.03	0.024	0.003	
	250	0.021	0.027	0.025	0.017	0.007	
	125	0.028	0.017	0.029	0.016	0.009	
	62.5	0.026	0.021	0.018	0.014	0.008	
	31.25	0.017	0.018	0.03	0.012	0.01	
	15.62	0.192	0.203	0.201	0.009	0.19	
	7.81	0.619	0.711	0.729	0.05	0.636	
	3.91	0.966	1.006	1.011	0.003	0.991	
	1.95	1.798	1.588	1.535	0.001	1.639	
	0.98	2.129	2.016	2.137	0	2.094	
	0.49	2.926	3.017	2.997	0	2.98	

code	conc. (µg mL⁻¹)	OD1	OD2	OD3	Blank	Reading	MIC (µg mL⁻¹)
S4 St. aureus	1000	0.052	0.044	0.051	0.048	0.001	15.62
	500	0.028	0.032	0.035	0.032	0	
	250	0.029	0.023	0.024	0.025	0	
	125	0.025	0.026	0.02	0.018	0.006	
	62.5	0.021	0.019	0.028	0.013	0.01	
	31.3	0.032	0.033	0.035	0.008	0.025	
	15.6	0.048	0.038	0.032	0.005	0.034	
	7.8	0.172	0.156	0.195	0.002	0.172	
	3.9	0.917	0.955	1.017	0.001	0.962	
	2	1.538	1.461	1.493	0	1.497	
	1	2.135	2.196	2.291	0	2.207	
	0.5	2.854	2.815	2.965	0	2.878	
code	conc. (µg mL⁻¹)	OD1	OD2	OD3	Blank	Reading	MIC (µg mL⁻¹)
S1 for E. coli	1000	0.043	0.055	0.054	0.05	0.001	31.25
	500	0.036	0.033	0.041	0.035	0.002	
	250	0.03	0.027	0.03	0.028	0.001	
	125	0.023	0.025	0.032	0.02	0.007	
	62.5	0.02	0.024	0.02	0.014	0.007	
	31.3	0.031	0.032	0.021	0.009	0.019	
	15.6	0.164	0.194	0.174	0.007	0.17	
	7.8	0.922	0.891	0.924	0.002	0.91	
	3.9	1.653	1.749	1.625	0.001	1.675	
	2	2.542	2.593	2.619	0	2.585	
	1	3.011	2.954	2.985	0	2.983	
	0.5	3.865	3.901	3.871	0	3.879	
code	conc. (µg mL⁻¹)	OD1	OD2	OD3	Blank	Reading	MIC (µg mL⁻¹)
S2 for E. coli	1000	0.043	0.053	0.05	0.047	0.002	125
	500	0.036	0.04	0.024	0.03	0.003	
	250	0.031	0.02	0.027	0.023	0.003	
	125	0.036	0.04	0.034	0.017	0.02	
	62.5	0.129	0.134	0.135	0.011	0.122	
	31.25	0.323	0.311	0.336	0.008	0.315	
	15.62	0.718	0.696	0.731	0.003	0.712	
	7.81	1.271	1.281	1.293	0.001	1.281	
	3.91	2.012	1.965	2.178	0.001	2.051	
	1.95	3.123	3.234	3.193	0.001	3.182	
	0.98	4.07	3.957	4.214	0	4.08	
	0.49	Over	4.134	Over	0	--------	
code	conc. (µg mL⁻¹)	OD1	OD2	OD3	Blank	Reading	MIC (µg mL⁻¹)
S3 for E. coli	1000	0.041	0.049	0.045	0.043	0.002	31.25
	500	0.025	0.03	0.021	0.024	0.001	

code	conc. (µg mL⁻¹)	OD1	OD2	OD3	Blank	Reading	MIC (µg mL⁻¹)
	250	0.022	0.023	0.017	0.017	0.004	
	125	0.02	0.019	0.023	0.016	0.005	
	62.5	0.019	0.021	0.025	0.014	0.008	
	31.25	0.034	0.03	0.026	0.012	0.018	
	15.62	0.143	0.159	0.163	0.009	0.146	
	7.81	0.719	0.917	0.931	0.05	0.806	
	3.91	1.388	1.419	1.526	0.003	1.441	
	1.95	2.138	2.011	1.966	0.001	2.037	
	0.98	3.134	3.188	3.213	0	3.178	
	0.49	3.675	3.754	3.832	0	3.754	
code	conc. (µg mL⁻¹)	OD1	OD2	OD3	Blank	Reading	MIC (µg mL⁻¹)
S4 for E. coli	1000	0.03	0.04	0.038	0.048	-0.012	15.62
	500	0.031	0.02	0.03	0.032	-0.005	
	250	0.021	0.027	0.025	0.025	-0.001	
	125	0.028	0.017	0.029	0.018	0.007	
	62.5	0.026	0.021	0.018	0.013	0.009	
	31.3	0.017	0.018	0.03	0.008	0.014	
	15.6	0.021	0.02	0.023	0.005	0.016	
	7.8	0.192	0.203	0.201	0.002	0.197	
	3.9	0.619	0.711	0.729	0.001	0.685	
	2	0.966	1.006	1.011	0	0.994	
	1	1.798	1.588	1.535	0	1.64	
	0.5	2.129	2.016	2.137	0	2.094	

Table S8. Effect of *Artemisia* Stems on HRBC hemolytic and membrane stabilization.

Artemisia Stem Conc. µg mL⁻¹	OD R1	OD R2	OD R3	Hypotonic Ab. Mean	Sample with Isotonic .solution Ab	Hemolysis % Inhibition	SD	SE
Control	0.984	0.987	0.982	0.984	0.001	0	0.003	0.001
1000	0.065	0.072	0.074	0.070	0.032	96.0	0.005	0.002
500	0.101	0.099	0.096	0.099	0.026	92.4	0.003	0.001
250	0.123	0.117	0.12	0.120	0.022	89.8	0.003	0.001
125	0.227	0.229	0.225	0.227	0.017	78.3	0.002	0.001
62.5	0.313	0.316	0.319	0.316	0.012	68.7	0.003	0.001
31.25	0.412	0.419	0.409	0.413	0.01	58.6	0.005	0.002
15.62	0.523	0.53	0.526	0.526	0.004	46.7	0.004	0.001
7.8	0.634	0.638	0.628	0.633	0.003	35.7	0.005	0.002
3.9	0.737	0.741	0.744	0.741	0.001	24.8	0.004	0.001

Table S9. Effect of *Ziziphus* Stems on HRBC hemolytic and membrane stabilization.

Ziziphus Stem Conc. μg mL^{-1}	OD R1	OD R2	OD R3	Hypotonic Ab. Mean	Sample with Isotonic .solution Ab	Hemolysis % Inhibition	SD	SE
Control	0.984	0.987	0.982	0.984	0.001	0	0.003	0.001
1000	0.044	0.047	0.048	0.046	0.032	98.5	0.002	0.001
500	0.083	0.088	0.085	0.085	0.026	93.8	0.003	0.001
250	0.107	0.106	0.108	0.107	0.022	91.2	0.001	0.000
125	0.201	0.195	0.192	0.196	0.017	81.5	0.005	0.001
62.5	0.301	0.309	0.311	0.307	0.012	69.7	0.005	0.002
31.25	0.387	0.385	0.391	0.388	0.01	61.2	0.003	0.001
15.62	0.467	0.472	0.475	0.471	0.004	52.3	0.004	0.001
7.8	0.59	0.603	0.608	0.600	0.003	39.1	0.009	0.003
3.9	0.718	0.715	0.716	0.716	0.001	27.2	0.002	0.000

Table S10. Cytotoxic activity of the synthesized nanoparticles against HepG2 human liver cancer cells determined by the MTT assay. Cell viability values represent mean ± SD of three independent experiments. IC_{50} values were calculated from dose–response curves using nonlinear regression analysis.

**Sample**	**μg mL⁻¹**		**O.D**		**Mean O.D**	**±SD**	**Cell Viability (%)**	**Cytotoxicity (%)**	**IC50 ± SD**
**Control (untreated cells) HepG2**	--------	0.722	0.725	0.728	0.725	0.0017	100	0.00	**μg**
**1**	250	0.019	0.017	0.018	0.018	0.0005	2.48	97.51	6.67 ± 0.06
	125	0.018	0.019	0.019	0.018	0.0003	2.57	97.42	
	62.51	0.019	0.021	0.020	0.019	0.0003	2.71	97.28	
	31.25	0.022	0.026	0.023	0.023	0.0012	3.26	96.73	
	15.62	0.120	0.142	0.138	0.133	0.0067	18.39	81.60	
	7.81	0.368	0.37	0.374	0.370	0.0017	51.12	48.87	
**2**	250	0.020	0.021	0.018	0.019	0.0008	2.71	97.28	9.13 ± 0.45
	125	0.026	0.022	0.025	0.024	0.0012	3.35	96.64	
	62.5	0.023	0.021	0.027	0.023	0.0017	3.26	96.73	
	31.25	0.053	0.047	0.05	0.050	0.0017	6.89	93.10	
	15.62	0.214	0.227	0.209	0.216	0.0053	29.88	70.11	
	7.81	0.416	0.397	0.401	0.404	0.0057	55.81	44.18	
**3**	250	0.022	0.022	0.021	0.021	0.0003	2.98	97.01	46.54 ± 0.45
	125	0.026	0.03	0.028	0.028	0.0011	3.86	96.13	
	62.50	0.170	0.163	0.147	0.161	0.0068	22.06	77.93	
	31.25	0.568	0.583	0.577	0.576	0.0043	79.44	20.55	
	15.62	0.720	0.718	0.727	0.721	0.0027	99.54	0.45	
	7.81	0.723	0.729	0.722	0.724	0.0021	99.95	0.04	
**4**	250	0.023	0.022	0.020	0.021	0.0008	2.98	97.01	11.26 ± 0.32
	125	0.021	0.027	0.026	0.024	0.0018	3.40	96.59	
	62.51	0.025	0.033	0.029	0.029	0.0023	4	96	
	31.25	0.031	0.028	0.035	0.031	0.0020	4.32	95.67	
	15.62	0.216	0.227	0.213	0.218	0.0042	30.16	69.83	
	7.81	0.453	0.441	0.468	0.454	0.0078	62.62	37.37	

Table S11. Effect of samples studied on A549 lung carcinoma at different concentrations and the toxicity and viability values of anticancer investigations.

ID	μg mL⁻¹	O.D			Mean O.D	±SE	Viability %	Toxicity %	IC50 ± SD
A549	--------	0.589	0.594	0.593	0.592	0.001528	100	0	ug
1	250	0.019	0.02	0.017	0.018667	0.000882	3.153153153	96.84684685	6.94 ± 0.54
	125	0.019	0.018	0.021	0.019333	0.000882	3.265765766	96.73423423	
	62.5	0.02	0.018	0.022	0.02	0.001155	3.378378378	96.62162162	
	31.25	0.023	0.02	0.022	0.021667	0.000882	3.65990991	96.34009009	
	15.62	0.16	0.143	0.128	0.143667	0.009244	24.26801802	75.73198198	
	7.81	0.3	0.297	0.316	0.304333	0.005897	51.40765766	48.59234234	
2	250	0.018	0.017	0.017	0.017333	0.000333	2.927927928	97.07207207	7.91 ± 0.45
	125	0.018	0.018	0.019	0.018333	0.000333	3.096846847	96.90315315	
	62.5	0.019	0.019	0.019	0.019	0	3.209459459	96.79054054	
	31.25	0.022	0.019	0.019	0.02	0.001	3.378378378	96.62162162	
	15.62	0.11	0.107	0.116	0.111	0.002646	18.75	81.25	
	7.81	0.328	0.32	0.314	0.320667	0.004055	54.16666667	45.83333333	
3	250	0.019	0.022	0.018	0.019667	0.001202	3.322072072	96.67792793	26.2 ± 0.54
	125	0.019	0.019	0.022	0.02	0.001	3.378378378	96.62162162	
	62.5	0.11	0.097	0.104	0.103667	0.003756	17.51126126	82.48873874	
	31.25	0.21	0.225	0.231	0.222	0.006245	37.5	62.5	
	15.62	0.436	0.448	0.462	0.448667	0.007513	75.78828829	24.21171171	
	7.81	0.578	0.597	0.583	0.586	0.005686	98.98648649	1.013513514	
4	250	0.019	0.018	0.019	0.018667	0.000333	3.153153153	96.84684685	16.86 ± 0.48
	125	0.019	0.022	0.02	0.020333	0.000882	3.434684685	96.56531532	
	62.5	0.021	0.026	0.024	0.023667	0.001453	3.997747748	96.00225225	
	31.25	0.026	0.033	0.027	0.028667	0.002186	4.842342342	95.15765766	
	15.62	0.213	0.245	0.263	0.240333	0.014621	40.59684685	59.40315315	
	7.81	0.513	0.533	0.542	0.529333	0.00857	89.41441441	10.58558559	

Table S12. Detailed viability, toxicity, and IC_{50} data for biosynthesized nanoparticles in DMSO against the non-cancerous WI-38 cell line.

ID	Conc. (μg mL⁻¹)	O.D (R1)	O.D (R2)	O.D (R3)	Mean O.D	±SE	Viability %	Toxicity %	IC50 (μg mL⁻¹)
Wi38	0 (no inhibitors)	0.698	0.693	0.691	0.694	0.002082	100	0	-----
1	250	0.674	0.679	0.684	0.679	0.002887	97.83861671	2.161383285	>>250
	125	0.689	0.693	0.69	0.690667	0.001202	99.5196926	0.480307397	
	62.5	0.692	0.696	0.69	0.692667	0.001764	99.80787704	0.192122959	
	31.25	0.69	0.697	0.698	0.695	0.002517	100.1440922	0	
	15.62	0.694	0.696	0.693	0.694333	0.000882	100.0480307	0	
	7.81	0.689	0.697	0.693	0.693	0.002309	99.85590778	0.144092219	
1..	500	0.642	0.648	0.648	0.646	0.002	93.08357	6.916427	>>500
	250	0.674	0.679	0.684	0.679	0.002887	97.83861671	2.161383285	
	125	0.689	0.693	0.69	0.690667	0.001202	99.5196926	0.480307397	
	62.5	0.692	0.696	0.69	0.692667	0.001764	99.80787704	0.192122959	
	31.25	0.69	0.697	0.698	0.695	0.002517	100.1440922	0	
	15.62	0.694	0.696	0.693	0.694333	0.000882	100.0480307	0	
2	250	0.22	0.218	0.238	0.225333	0.00636	32.46878002	67.53121998	208.71 ± 2.65
	125	0.664	0.658	0.647	0.656333	0.004978	94.57252642	5.427473583	
	62.5	0.687	0.692	0.69	0.689667	0.001453	99.37560038	0.624399616	
	31.25	0.691	0.687	0.695	0.691	0.002309	99.56772334	0.432276657	
	15.62	0.696	0.692	0.692	0.693333	0.001333	99.90393852	0.096061479	
	7.81	0.694	0.698	0.695	0.695667	0.001202	100.2401537	0	
3	250	0.308	0.311	0.302	0.307	0.002646	44.23631124	55.76368876	233.68 ± 0.9
	125	0.686	0.679	0.676	0.680333	0.002963	98.03073967	1.969260327	
	62.5	0.688	0.679	0.688	0.685	0.003	98.70317003	1.296829971	
	31.25	0.692	0.687	0.695	0.691333	0.002333	99.61575408	0.384245917	
	15.62	0.696	0.693	0.692	0.693667	0.001202	99.95196926	0.04803074	
	7.81	0.697	0.692	0.693	0.694	0.001528	100	0	
4	250	0.599	0.603	0.609	0.603667	0.002906	86.98366955	13.01633045	>>250
	125	0.687	0.69	0.685	0.687333	0.001453	99.03938521	0.960614793	
	62.5	0.687	0.691	0.693	0.690333	0.001764	99.47166186	0.528338136	
	31.25	0.69	0.695	0.691	0.692	0.001528	99.71181556	0.288184438	
	15.62	0.693	0.691	0.695	0.693	0.001155	99.85590778	0.144092219	
	7.81	0.693	0.699	0.695	0.695667	0.001764	100.2401537	0	
4..	500	0.567	0.572	0.573	0.570667	0.001856	82.22863	17.77137	>>500
	250	0.599	0.603	0.609	0.603667	0.002906	86.98366955	13.01633045	
	125	0.687	0.69	0.685	0.687333	0.001453	99.03938521	0.960614793	
	62.5	0.687	0.691	0.693	0.690333	0.001764	99.47166186	0.528338136	
	31.25	0.69	0.695	0.691	0.692	0.001528	99.71181556	0.288184438	
	15.62	0.693	0.691	0.695	0.693	0.001155	99.85590778	0.144092219	

Table S13. Detailed experimental results from the scratch wound healing assay, including raw wound area measurements and microscopic observations for WI-38 cells treated with the four nanoparticle samples.

ID	length	Width initial	Area initial	length	Width final	Area final
**Cont. Wi38**	1076.03	1274.025	1370889.121	1076.03	270	290528.1
	1078.002	1276.025	1375557.502	1078.002	158	170324.3
	1074.002	1316.002	1413388.78	1074.002	346.006	371611.1
	1074.002	1348.024	1447780.472	1074.002	388.021	416735.3
	1074.002	1306.006	1402653.056	1074.002	346.023	371629.4
	1078.067	1304.014	1405814.461	1078.067	244	263048.3
**1 250 µg**	1076.03	1468.001	1579613.116	1076.03	184.011	198001.4
	1078.002	1460.067	1573955.146	1078.002	82.098	88501.81
	1074.002	1478	1587374.956	1074.002	202.01	216959.1
	1074.002	1430.022	1535846.488	1074.002	166	178284.3
	1074.002	1470.022	1578806.568	1074.002	60	64440.12
	1078.067	1536.22	1656148.087	1078.067	208	224237.9
**2 125 µg**	1076.03	1588	1708735.64	1076.03	150.12	161533.6
	1078.002	1502.001	1619160.082	1078.002	264.189	284796.3
	1074.002	1556.032	1671181.48	1074.002	236.136	253610.5
	1074.002	1584.102	1701328.716	1074.002	404.02	433918.3
	1074.002	1580.032	1696957.528	1074.002	264.03	283568.7
	1078.067	1600.08	1724993.445	1078.067	262.069	282527.9
**3 125 µg**	1076.03	1692.011	1820654.596	1076.03	132	142036
	1078.002	1670.005	1800268.73	1078.002	144	155232.3
	1074.002	1668.145	1791591.066	1074.002	24.083	25865.19
	1074.002	1672.097	1795835.522	1074.002	92.087	98901.62
	1074.002	1640.03	1761395.5	1074.002	66.121	71014.09
	1078.067	1714.005	1847812.228	1078.067	206.01	222092.6
**4 250 µg**	1076.03	1328.074	1429047.466	1076.03	292	314200.8
	1078.002	1326.054	1429488.864	1078.002	276.065	297598.6
	1074.002	1296.006	1391913.036	1074.002	142	152508.3
	1074.002	1436.139	1542416.158	1074.002	148.216	159184.3
	1074.002	1384.006	1486425.212	1074.002	120.067	128952.2
	1078.067	1430.006	1541642.278	1078.067	156.461	168675.4

Supplementary Section S1. Summary of MIC and MBC Determination Procedures for Biosynthesized Nanoparticles

Preparation of microbial inoculum

Test microorganisms (Staphylococcus aureus ATCC 6538 and Escherichia coli ATCC 8739) were grown in Mueller–Hinton broth. Fresh cultures were adjusted to a turbidity equivalent to 0.5 McFarland standard to obtain a standardized inoculum.

Preparation of nanoparticle dilutions

Biosynthesized silver nanoparticle preparations were serially diluted two-fold in Mueller–Hinton broth to obtain concentrations of 1000, 500, 250, 125, 62.5, 31.25, 15.625, 7.81, and 3.9 μg/mL. Dilutions were prepared in sterile 96-well microplates (or tubes, as applicable).

MIC assay

Each well containing a defined nanoparticle concentration was inoculated with the standardized microbial suspension. Plates were incubated at 35 °C for 16–24 h under static conditions. Microbial growth was assessed by visual inspection for turbidity and confirmed by spectrophotometric measurement of optical density at 630 nm using a microplate reader (BioTek 800 TS). The MIC was recorded as the lowest nanoparticle concentration that completely inhibited visible growth compared with growth controls. All experiments were performed in technical triplicate and repeated on three independent occasions.

MBC assay

To determine the minimum bactericidal concentration (MBC), aliquots from wells corresponding to the MIC and at least two higher nanoparticle concentrations were subcultured onto drug-free Mueller–Hinton agar plates and incubated at 35 °C for 24 h. Colony-forming units (CFU/mL) were enumerated, and the MBC was defined as the lowest nanoparticle concentration that achieved a ≥99.9% (≥3-$\log_{10}$) reduction in viable CFU/mL relative to the initial inoculum.

Supplementary Section S2. Calculations for Scratch Wound Healing Assay:

Calculations

Rat of migration (RM) = (Wi – Wf) / t

Wi =average of initial wound width (μm); Wf= average of final wound width (μm); T = time span of the assay in hours

Percentage of wound closure % ={ ($A_{t=0}$ – $A_{t=\Delta t}$) / $A_{t=0}$ } 100

$A_{t=0}$ = initial wound area; $A_{t=\Delta t}$ = wound area after n hours

Area difference % = (Ai – Af)

Ai = initial area ; Af = final area

Supplementary Section S3. The sample preparation protocol for DLS and zeta potential measurements

For DLS and zeta potential measurements, nanoparticle suspensions were diluted with 0.22 μm filtered deionized water to achieve optimal scattering intensity (100–300 kcps) and avoid multiple scattering, then filtered through 0.45 μm PVDF syringe filters to remove large aggregates and dust. Samples (1.0 mL) were loaded into disposable polystyrene cuvettes pre-rinsed three times with the filtered suspension and allowed to stand for 5 min to eliminate air bubbles. After a 30 min instrument warm-up, each sample was equilibrated at 25°C for 120 s, and three independent measurements (each comprising 10–15 automatic sub-runs) were performed. Reported hydrodynamic diameters and zeta potential values are expressed as mean ± standard deviation of triplicate measurements, accepted only when the polydispersity index (PDI) was < 0.5, the correlation function showed no baseline drift, the count rate was stable (coefficient of variation < 5%), and the attenuator position remained consistent.

Supplementary Section S4. The sample preparation protocol for XRD measurements

Silver nanoparticle suspensions were centrifuged at 10,000 rpm for 15 min, and the pellets were washed three times with deionized water to remove residual synthesis reagents, then dried in a vacuum oven at 60°C for 12 h. The dried aggregates were gently ground in an agate mortar and pestle to obtain a homogeneous powder, which was pressed onto zero-background silicon sample holders to a thickness of 1–2 mm. Diffractometer calibration was performed using NIST SRM 640e silicon powder prior to measurements, and diffraction patterns were collected in continuous scan mode under controlled ambient conditions (25 ± 2°C, 40–60% relative humidity), with each sample analyzed in triplicate.
